# Unraveling Human Hepatocellular Responses to PFAS and Aqueous Film-Forming Foams (AFFFs) for Molecular Hazard Prioritization and In Vivo Translation

**DOI:** 10.1021/acs.est.4c10595

**Published:** 2025-02-02

**Authors:** Kevin A. Mauge-Lewis, Sreenivasa C. Ramaiahgari, Scott S. Auerbach, Georgia K. Roberts, Suramya Waidyanatha, Suzanne E. Fenton, Dhiral P. Phadke, Michele R. Balik-Meisner, Arpit Tandon, Deepak Mav, Brian Howard, Ruchir Shah, Barney Sparrow, Jenni Gorospe, Stephen S. Ferguson

**Affiliations:** † Division of Translational Toxicology, National Institute for Environmental Sciences, 111 TW Alexander Drive, Durham, North Carolina 27709, United States; ‡ Sciome, 1920 NC-54 Suite 510 & 520, Durham, North Carolina 27713, United States; § Battelle, 505 King Avenue, Columbus, Ohio 43201, United States

**Keywords:** PFAS, AFFF, environmental health, biological-response similarity, human liver injury, transcriptomic response pathways, PPARα, CAR, VDR, hepatomegaly, prioritization, translation

## Abstract

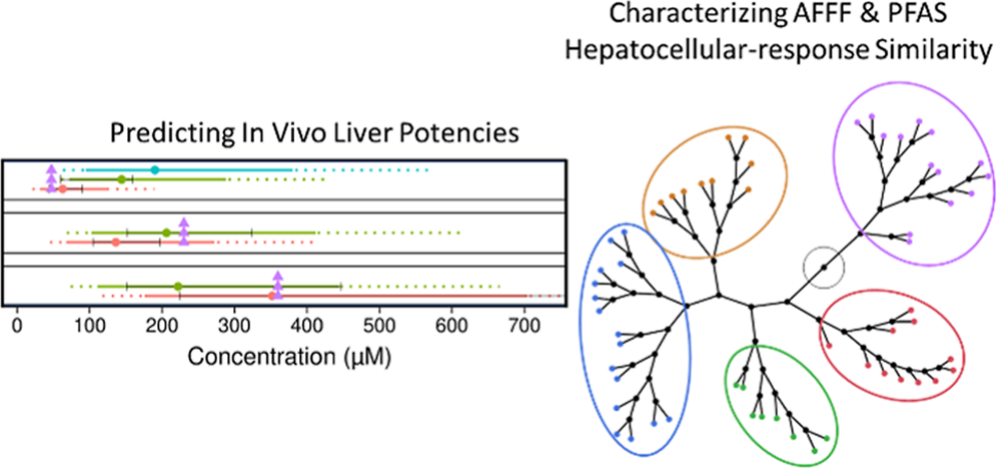

Aqueous film-forming foams (AFFFs) are complex product mixtures that often contain per- and polyfluorinated alkyl substances (PFAS) to enhance fire suppression and protect firefighters. However, PFAS have been associated with a range of adverse health effects (e.g., liver and thyroid disease and cancer), and innovative approach methods to better understand their toxicity potential and identify safer alternatives are needed. In this study, we investigated a set of 30 substances (e.g., AFFF, PFAS, and clinical drugs) using differentiated cultures of human hepatocytes (HepaRG, 2D), high-throughput transcriptomics, deep learning of cell morphology images, and liver enzyme leakage assays with benchmark dose analysis to (1) predict the potency ranges for human liver injury, (2) delineate gene- and pathway-level transcriptomic points-of-departure for molecular hazard characterization and prioritization, (3) characterize human hepatocellular response similarities to inform regulatory read-across efforts, and (4) introduce an innovative approach to translate mechanistic hepatocellular response data to predict the potency ranges for PFAS-induced hepatomegaly in vivo. Collectively, these data fill important mechanistic knowledge gaps with PFAS/AFFF and represent a scalable platform to address the thousands of PFAS in commerce for greener chemistries and next-generation risk assessments.

## Abstract 2

Environmental PFAS and AFFFs investigated to predict potency ranges for human liver injury, unravel hepatocellular-response similarities, and delineate mechanistic pathways that translated into predictions for rodent hepatomegaly.

## Introduction

Aqueous film-forming foams (AFFFs) are used to rapidly extinguish hydrocarbon-based fuel fires and protect fire fighters^1,2^ in both civilian and military applications (e.g., airports, refineries, and ships).^3^ AFFF products often contain per- and poly fluorinated alkyl substances (PFAS), which provide superior fire protection to firefighters but are known to be environmentally persistent, often called “forever chemicals”.^4−6^ Widespread production and use of PFAS, including AFFFs, has led to contamination of rivers, drinking water, surface waters, groundwater, air, house dust, and food.^7^ Virtually, all Americans have detectable levels of multiple PFAS in their blood serum from birth, with higher levels resulting from occupational exposures, foods, and drinking water.^8−10^

Some PFAS are known to bioaccumulate in mammals from environmental exposures, which can increase their potential for adverse health effects.^11−13^ A recent 2023 report to the US Congress noted PFAS associations to numerous adverse health outcomes including cancer, liver injury/metabolic disease, thyroid disease, developmental/reproductive harm, and cardiovascular disease.^14^ Much of the research has mechanistically linked perfluorooctanesulfonate (PFOS) and perfluorooctane carboxylate (PFOA) to altered lipid metabolism (e.g., serum cholesterol), peroxisome proliferation, nuclear receptor activation, and mitochondrial bioenergetics.^12,15^ PFOS and PFOA have been phased out of production, leading to diminished environmental levels. However, alternative forms of PFAS (e.g., fluorotelomers) are emerging with a growing number of similar associations to adverse health effects.^16−19^ Given the broadening array of PFAS being discovered in commercial products (e.g., clothing and personal care products), environmental sampling, and human biomonitoring studies, the complexity of PFAS toxicity continues to increase.

Liver is a primary target organ for many PFAS toxicities with associations to hepatomegaly, endocrine disruption, and fatty liver disease that can progress to inflammation and liver injury.^13,15,20,21^ A growing number of effects are linked to activation of nuclear receptor pathways and accumulation of PFAS in tissues due to chronic environmental exposures.^22−24^ Advances in liver tissue models, assay systems, and computational tools now enable translational toxicology screening to investigate these complex challenges.

The aim of this study was to address critical data gaps in environmental health related to PFAS and AFFF while extending emerging science to scalable solutions for safer product development. Our study investigated 30 substances in differentiated cultures of human hepatocytes using high-throughput transcriptomics, deep learning analysis of cell morphology images, and liver enzyme leakage assays. Our findings revealed potency ranges for human liver injury with PFAS and AFFF products alongside human drug substances, derived mechanistic pathway potencies and prioritized substances by molecular hazard pathways (i.e., PPARα, CAR), delineated biological-response similarity relationships that fill important data gaps and inform regulatory read-across efforts, and introduced a novel approach using internal dose metrics to estimate the potency ranges for PFAS-induced hepatomegaly by hepatic receptor pathway potencies.^25,26^ Overall, the study represents a promising extension of emerging science into toxicological applications for greener chemistries and next-generation risk assessments.

## Materials and Methods

### Test Substances and Exposure Media

Five MIL-SPEC-qualified AFFFs, confirmed on the Department of Defense Qualified Products Database (https://qpldocs.dla.mil/search/parts.aspx?qpl=1910) at the time of study initiation, were obtained from various sources, as summarized in Table S1 for all test substances. An equal-volume pool of 5 AFFF concentrates was prepared (i.e., AFFF-Qpool) to evaluate the aggregate effects of AFFF exposures across products. DMSO and MeOH were purchased from Sigma-Aldrich, St. Louis, MO (product no. 276855, CASRN: 67-68-5, purity: 99.0%). Final exposure media were prepared by dilution of test substance stock solutions to 1× concentrations in William’s E Medium (Thermo Fisher Scientific, catalog no. A1217601), supplemented with pen-strep (Sigma, batch no. 0000109048) and either HepaRG Thawing/Plating Medium Supplement (MHTAP) (Lonza, batch no. ADD411025) for DMSO-solubilized substances or Pre-Induction and Tox Additive Supplement (MHPIT) for water/MeOH-solubilized substances. All final DMSO concentrations were ≤0.3% (v/v).

### Cell Culture

Cryopreserved NoSpin HepaRG cells (MMHPR116, human liver, NSHPRG) were obtained from Lonza (Durham, NC). Collagen(I)-coated cell culture plates (384-well, catalog: 356667) were obtained from Corning (Corning, NY). HepaRG cells were thawed and plated as described previously.^27^ Here, 10 vials of cryo-HepaRG cells were thawed and transferred to MHTAP. Viable cell numbers were measured using a hemacytometer, and cell densities were adjusted to ∼20,000 cells/well. Cell cultures were maintained in a humidified incubator at 5% CO_2_ and 37 °C with culture medium supplemented with diluted MHPIT (i.e., 3 × 100 mL supplements per 500 mL of WEM) that reduce baseline DMSO concentrations to ∼0.3%.

Test substance stock solutions were solubilized/diluted, as summarized in Table 1. Dissolution/solubility was confirmed by visual inspection, and serial dilutions at half-log concentrations were made over 9 exposure levels. Cell morphology photomicrographs (phase contrast) for each well were captured using an Incucyte Zoom (Essen Bioscience, Ann Arbor, MI). All liquid handling was performed via calibrated Integra VIAFLO 384 (Integra Biosciences, Hudson, NH, catalog: 6031).

**Table 1:** Summary of Test Substance Exposure Media Preparation for Transcriptomic Analysis

substance identifier number	test substance abbreviated name	CASRN	formula weight (g/mol)	avehicle solvent	final vehicle concentration (% v/v)	maximum exposure (μM)	maximum exposure (% v/v)
20	2-(2-butoxyethoxy)ethanol	112-34-5	162.23	MHPIT	0.3		0.06
22	2-methyl-2,4-pentanediol	107-41-5	118.17	MHPIT	0.3		0.06
25	4,2-FTS	757124-72-4	328.15	MHPIT	0.3	500	
12	6,2-FTOH	647-42-7	364.1	MeOH	0.1	500	
4	6,2-FTS	27619-97-2	428.16	MHPIT	0.3	500	
8	6,2-methacrylate	2144-53-8	432.18	MHPIT	0.3	500	
13	8,2-FTOH	678-39-7	464.12	MeOH	0.1	500	
26	AFFF1	N/A	N/A	MHPIT	0.3		0.2
27	AFFF2	N/A	N/A	MHPIT	0.3		0.06
28	AFFF3	N/A	N/A	MHPIT	0.3		0.6
29	AFFF4	N/A	N/A	MHPIT	0.3		0.6
30	AFFF5	N/A	N/A	MHPIT	0.3		0.6
17	cyclosporin A	59865-13-3	1202.6	DMSO	0.1	30	
21	laurylamidopropyl betaine	4292-10-8	342.52	MHPIT	0.3		0.02
15	OMP	73590-58-6	345.4	DMSO	0.1	100	
9	PB	57-30-7	254.2	MHPIT	0.3	1000	
19	PEG	25322-68-3	N/A	MHPIT	0.3		0.6
3	PFBS	375-73-5	300.09	MHPIT	0.3	500	
11	PFDA	335-76-2	514.08	MeOH	0.1	500	
7	PFHpA	375-85-9	364.06	MHPIT	0.3	500	
1	PFHpS	375-92-8	450.12	MHPIT	0.3	500	
6	PFHxA	307-24-4	314.05	MHPIT	0.3	500	
2	PFHxS	3871-99-6	438.21	MHPIT	0.3	500	
10	PFNA	375-95-1	464.07	MeOH	0.1	125	
5	PFOA	335-67-1	414.07	MHPIT	0.3	500	
14	PFOS	1763-23-1	500.13	DMSO	0.1	250	
24	AFFF-QPool	N/A	N/A	MHPIT	0.3		0.2
23	S-550	N/A	N/A	MHPIT	0.3		0.02
18	SOS	142-31-4	232.27	MHPIT	0.3		0.06
16	Wyeth-14,643	50892-23-4	323.8	DMSO	0.1	200	

a MHPIT represents standard HepaRG plating and induction medium that was used to solubilize some test articles.

### Assay Exposures

Initial dose range-finding was performed by ATP depletion (CellTiter-Glo, Promega, Madison, WI) as described in Supporting Information (Table S2 and Figure S3) to identify cytotoxic exposure levels excluded from transcriptomic assays. High-throughput transcriptomics were multiplexed with liver enzyme leakage (i.e., lactate dehydrogenase), and cell morphology photomicrographs were captured for each assay well in 2 independent experiments, each spanning 9 half-log exposure levels with quadruplicate technical replicates. Liver enzyme leakage assays (LDH-Glo, Promega, Madison, WI) were performed on spent cell culture media from each transcriptomic assay well after 96 h exposures, as previously described.^27^ Lysates for transcriptomic assays were prepared by washing cell monolayers twice with phosphate-buffered saline, addition of 10 μL of 2× BioSpyder Lysis Buffer (Biospyder, Carlsbad, CA), and 15 min incubation at room temperature prior to freezing of lysate plates at −80 °C. Samples were subsequently shipped frozen to BioSpyder (Carlsbad, CA) for TempO-Seq analysis, as previously described,^27,28^ using the S1500^+^(v.2) transcriptomic probe set (3,368 probes).^29^

### Deep Learning of Cell Morphology Photomicrographs

Cell morphology photomicrographs (phase contrast) for each assay well were captured prior to exposures and following 96 h exposures (∼2400 images) using an Incucyte Zoom (Essen Bioscience, Ann Arbor, MI). The original, high-resolution images were 1392 × 1036 pixels in size and subsequently split into 4 × 4 grids of 348 × 259 tiles. A pretrained ResNet50 convolutional neural network (CNN) model, fine-tuned on differentiated HepaRG cells, published in Tandon et al.,^30^ was used to calculate the probability score that each individual tile was “altered” (i.e., exhibiting visible morphological changes compared to vehicle controls). Chemicals for which none of the tiles were altered were filtered from the downstream analysis. The remaining image-level probability scores were analyzed using the double index beta regression model.^31^ The logit link function associated predicted mean and dosage concentration; the log link function was used to model the precision parameter. Model parameters were estimated with the maximum likelihood method using the “betareg.fit” function of the “betareg” R-package. The effective concentration at the tenth percentile potencies (EC_10_) and corresponding confidence intervals were estimated using the asymptotic Delta method.

### Transcriptomic Data Analysis

Raw instrument FASTQ files were mapped to probe targets and assessed for sequencing quality, as described previously.^27^ Here, FASTQ sequencing data files were securely transferred to initiate data quality review. FastQC version 0.11.3 was run to verify that each sample passed basic sequencing quality metrics (e.g., per-base sequence quality and per-base N content). Transcriptomic data were processed by aligning reads to the probe sequences using Bowtie^32^ version 1.2.2 with the following parameters: -v 3 -k 1 -m 1 --best --strata. This configuration allowed up to three mismatches and reported the single best alignment. The read counts for each probe were obtained using samtools idxstats. Read counts for attenuated probes were adjusted to unattenuated equivalent counts using the attenuation factors provided in the platform manifest. To account for between-sample sequencing depth variation, unattenuated read counts were normalized at the probe level by applying reads-per-million normalization. Finally, a pseudoread-count of 1.0 was added to each normalized expression value, and values were log_2_ transformed. For each sample, the total sequenced reads, the percentage of reads aligning to the platform manifest, the alignment rate, and the percentage of expressed probes (≥5 reads per probe) were calculated. Samples with values below the following thresholds were removed from subsequent analysis: sequencing depth <300 K, total alignment rate <40%, unique alignment rate <30%, number of aligned reads <300 K, or percentage of probes with at least five reads <50%. Filtering on the percentage of expressed probes eliminated biased samples for which the sequenced reads only reflect a small portion of the measured transcriptome. Furthermore, principal component analysis (PCA), hierarchical clustering, and inter-replicate correlation analyses were performed. Pearson correlation was computed for each sample with each of its replicates. Within a treatment group, outlier samples were removed before downstream analysis based on the following criteria: median pairwise inter-replicate correlation for a given sample differed by more than 0.05 from the median correlation from other replicates.

Processed transcriptomic data were analyzed with BMDExpress 2.3 in 2 modes, as previously described,^27^ to derive gene- and pathway-level biological responses. Here, gene expression data were analyzed both in units of percent (%) dilution (volume per volume) and μM concentration. For the 5 AFFF products, AFFF-Qpool, and S-550 product, given our lack of detailed information on their precise constituent compositions, average μM concentrations were estimated using a suppositional average molecular weight of 300 Da to enable molar-based relative potency comparisons to individual chemicals in a semiquantitative approach. The rationale for selection of 300 Da was based on our current understanding of small molecules comprising cell culture medium used to dilute AFFF products (i.e., <0.6% final exposure levels) and the molecular weight ranges of reported AFFF constituents (e.g., PFAS fluorotelomers). Transcriptomic data were prefiltered using the William’s trend test with thresholds of 2.0-fold change and *p* < 0.05. Next, data meeting these thresholds were analyzed using 9 model fits (i.e., Hill, power, linear, poly 2, poly 3, exponential 2, exponential 3, exponential 4, exponential 5) to determine the best-fit benchmark concentration for each probe–substance pair. A confidence level of 0.95 and a defined benchmark response (BMR) of 1.0 SD were applied, except for hierarchical clustering (described below). Derived benchmark concentrations (BMCs) were further analyzed by Functional Classification using the Wikipathways (MSigDB version 7.2) with specific annotations for PFAS-related nuclear receptor pathways and visualize accumulation plots^33^ using the following criteria: BMDs with fitPValue <0.001, BMDU/BMDL >40.0, BMDs more than 10-fold below the lowest positive dose, and fold-change <2.0 were removed.

Publicly available data files can be accessed at 10.22427/NTP-DATA-500-017-001-000-4. A list of downloadable data files with brief descriptions is provided in the Supporting Information for Publication. Data files (e.g., raw, processed, analyzed data files, and cell morphology images) include BMDExpress transcriptomic data analysis files (% and μM concentration units), liver injury rankings, and exported pathway-level BMCs.

Venn diagrams were visualized using online molbiotools (https://molbiotools.com/listcompare.php) to compare derived BMC gene- and pathway-level similarities. For this, hierarchical clustering of a filtered set of the 100 most potent BMCs (i.e., lowest BMCs) and/or the 100 highest maximum fold-change BMCs was performed to further explore biological response similarity. For this, log_2_ normalized mapped read count files were uploaded into BMDExpress 3.0 and prefiltered by Prefilter Curve Fit tool at 3SD BMR Factor, constant variance, NOTEL/LOTEL >2, and *p* < 0.05 (*T*-test). These responses were further analyzed with the EPA BMDS MLE Hill model with a defined BMR = 1SD and confidence interval of 0.95. BMCs were functionally classified to remove BMCs greater than the highest dose or BMD/BMD >20. BMCs were rank-scored and directionally adjusted (±) for each independent experimental run using R. Scores were merged into a matrix, and missing values were assigned a “0”. This matrix was loaded into JMP 16.0 (SAS, Cary, NC) for hierarchical clustering using Ward’s method with default settings resulting in 5 identified clusters.

## Results and Discussion

### Evaluating the Potential for Human Liver Injury

**Figure 1:**
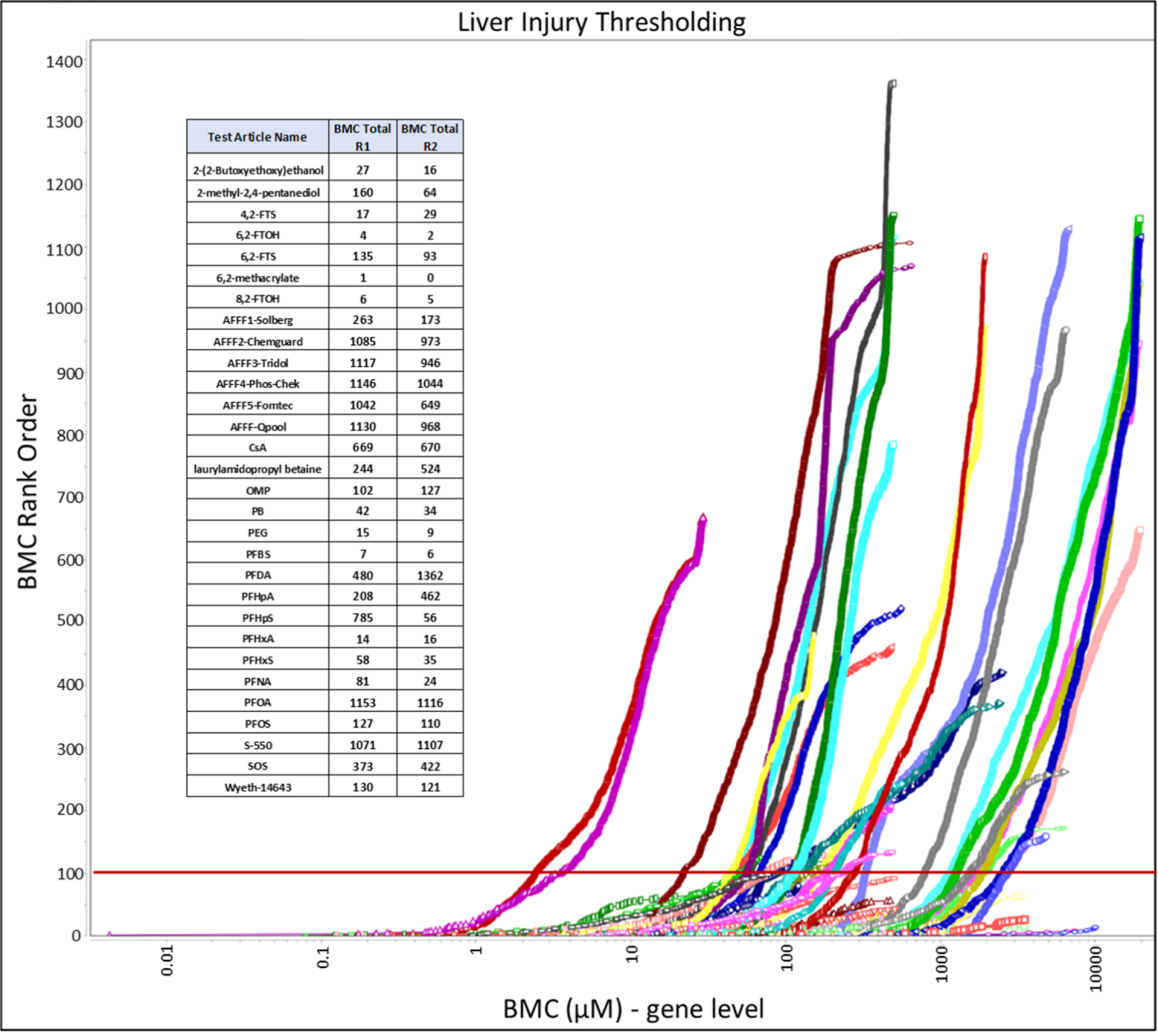
Accumulation plot summarizing the potency-ordered distribution of transcriptomic benchmark concentrations (BMCs) versus their respective BMC rank orders (i.e., independent experiments Run1 and Run2) with tabulated BMC totals. Red line portrays the statistically defined liver injury threshold at the 105^th^ potency-ordered BMC (i.e., BMC105) to estimate potency ranges for human liver injury, as described in Materials and Methods. A suppositional average constituent molecular weight of 300 Da was used for 7 AFFF-related products to enable relative comparisons, as described in Materials and Methods.

Differentiated cultures of human hepatocytes were established, exposed to 30 substances (e.g., AFFF, PFAS, clinical drugs), and assayed with the combination of high-throughput transcriptomics (3,386 probes), liver enzyme leakage, and deep learning of cellular morphologies. Figure 1 summarizes the potency-ordered distribution of 22,856 transcriptomic points-of-departure (gene-level) in the form of benchmark concentrations (BMCs) for two independent experimental runs in μM units. The red line portrays the statistically defined BMC threshold (i.e., 105^th^ BMC, BMC105), based on a previous qualifying study,^27^ that distinguished exposure concentrations with high potential for human liver injury. Transcriptomic data were analyzed in concentration units of percent (%) dilution and micromolar (μM), yielding comparable BMCs with unit conversion (Table S4). Table 2 summarizes derived BMC105 liver injury thresholds sorted by their respective potencies (i.e., low to high), with a complete list for each independent experiment provided in Supporting Information file “BMC105 Rankings-final-3.xlsx”. Twenty substances crossed the threshold for substance-induced liver injury (SILI) classification over the exposure ranges evaluated. Overall, mean transcriptomic BMC105 values were more sensitive than corresponding mean liver enzyme leakage potencies (>2.5-fold) and 10 to 100 times less potent than the lowest derived mean transcriptomic BMC. Cell morphology analysis using a novel deep learning convolutional neural network (CNN) confirmed hepatocyte culture fidelity for each assay well, relative to vehicle controls, using a computationally derived metric that was further analyzed to derive points-of-departure analysis (EC_10_, Table 2). Analogous to liver enzyme leakage, mean potencies for cell morphology change were less sensitive than the transcriptomic BMC105 thresholds. However, in many cases, deep learning of cell morphologies was sufficient to rapidly identify SILI-classifying substances and serves as a cost-effective computational approach for tiered assay evaluations.

**Table 2:** Mean Potencies from 2 Independent Experiments to Estimate the Potencies for Human Liver Injury with Liver Enzyme Leakage Assays, Transcriptomic Thresholds, and Deep Learning Analysis of Cell Morphology^a^^,^^d^

test substance name	liver enzyme leakage BMC (μM^b^)	transcriptomic BMC105 (μM^b^)	morphology EC_10_ (μM^b^)^c^	human liver injury classification^c^
CsA	4.77	3.0	7.0	SILI
S-550^‡^	284	39	equivocal	SILI
PFDA	115	52	equivocal	SILI
OMP	97^c^	77	NC	SILI
PFOA	83	81	102	SILI
Wyeth-14,643	114	85	NC	SILI
SOS	558	115	564	SILI
PFHpA	287	117	23	SILI
PFNA	80^c^	125	NC	SILI
laurylamidopropyl betaine	156	138	equivocal	SILI
PFOS	209^c^	189.5	NC	SILI
AFFF2^‡^	1240	225	469	SILI
PFHpS	192	308	103	SILI
6,2-FTS	419	372	NC	equivocal
AFFF-Qpool^‡^	5162	559	887	SILI
AFFF4^‡^	4082	1228	7530	SILI
AFFF1^‡^	2215	1793	NC	SILI
AFFF3^‡^	6943	2132	6333	SILI
AFFF5^‡^	3401	2298	7480	SILI
2-methyl-2,4-pentanediol	NC	3913	NC	equivocal
PFHxS	188^c^	NC	NC	non-SILI
PB	NC	NC	NC	non-SILI
4,2-FTSA	NC	NC	NC	non-SILI
2(2-butoxyethoxy)ethanol	NC	NC	NC	non-SILI
PFHxA	203^c^	NC	NC	non- SILI
PEG	NC	NC	NC	non-SILI
PFBS	346^c^	NC	NC	non-SILI
8,2-FTOH	NC	NC	NC	non-SILI
6,2-FTOH	NC	NC	NC	non-SILI
6,2-methacrylate	NC	NC	NC	non-SILI

a Substance-induced liver injury (SILI) classifications are also tabulated.

b NC = BMD values that were noncalculable through BMDExpress and deep learning analysis.

c Equivocal designations indicated only 1 of 2 experimental runs produced a discernible BMC for a given end point, which generally was observed near the highest exposure evaluated.

d For AFFF-related product mixtures (‡), a suppositional average constituent molecular weight of 300 Da was used for relative comparisons as described in Materials and Methods.

Cyclosporine A (CsA) was the most potent liver injury response, with an average BMC105 of ∼3 μM (Table 2). This is consistent with therapeutic exposure levels (i.e., *C*_max_: 0.2 to 1.2 μM)^34^ and numerous human clinical associations to cholestatic liver injury. Due to overt hepatocellular toxicity, each of the 5 AFFF products, along with the AFFF-Qpool, was diluted below their recommended 3% application concentrations with maximum exposures at or below 0.6% (v/v) following dose range-findings (Table S2 and Figure S3). For relative potency comparisons, a suppositional average molecular weight of 300 Da was explored to enable molar-based characterization across substances (rationale described in Materials and Methods). AFFF-related products ranked intermediate across test substances and lower among SILI-classifying substances, likely due to their extensively diluted exposure levels (≤0.6%) estimated to have <0.1% PFAS. However, the S-550 product mixture, thought to be composed primarily of 6-carbon fluorotelomers and PEG (not biologically active in our test system), ranked high for human liver injury and suggested constituent PFAS may contribute to hepatocellular injury responses. Given the bioaccumulative nature of some PFAS and proportionally low concentrations in diluted AFFF products, it is important to note that derived potency values from this shorter-term exposure study (i.e., 96 h) should be interpreted with care, and further evaluations that sufficiently model PFAS accumulation over longer-term exposures (e.g., 28 days) are warranted to address subchronic and chronic toxicity potential. Non-PFAS constituents of AFFF included laurylamidopropyl betaine and sodium octyl sulfate (SOS) that were among the more potent substances evaluated. However, lower bioaccessibility may limit potential for liver toxicity potential.^35^ PFAS, PFDA, PFOA, PFHpA, PFNA, PFOS, PFHpS, and 6,2-FTS exposures yielded hepatotoxic responses, while PFHxS, 4,2-FTS, PFHxA, PFBS, 8,2-FTOH, 6,2-FTOH, and 6,2-methacrylate were largely inactive up to 500 μM concentrations. This in vitro screening platform is susceptible to loss of semivolatile PFAS (e.g., fluorotelomer alcohols) by evaporation, which may limit its context of use for some PFAS. A BMC105 of 190 μM was observed for PFOS after 96 h, which was notably less potent than the cytotoxic BMCs derived from our previous collaborative study with Health Canada over longer-term (14 day) repeated exposures in liver microtissues.^36^ This is consistent with a cumulative degree of PFOS accumulation that may be operative in microtissue models but may also under-reflect tissue-to-blood ratios observable with the higher cell numbers used in this study (i.e., ∼20,000 vs ∼2,000).

### Molecular Hazard Identification and Prioritization

PFAS and AFFF have been linked to multiple mechanisms of toxicity. In this study, we explored a broad range of transcriptomic response pathways with PFAS-containing substances, alongside clinical drugs, to unravel their relative molecular hazard potential using relative potencies to develop prioritization methods in support of green chemistry research. A total of 23,484 gene-level BMCs were derived and mapped to 12,041 biological response pathways (i.e., Wikipathways, 579 annotated gene sets, MSigDB7.2) using BMDExpress 2.3 (“Final export of identified pathways (3).xlsx”). To demonstrate the fidelity of the cell system and its proficiency to model human health effects, three reference drugs (i.e., cyclosporin A, omeprazole, and phenobarbital) with established clinical associations to PFAS-associated hepatic receptor pathways were included as translational anchors, as further described below. Given the breadth of pathways identified in these research data, we focused our analysis on pathways with high-frequency BMC identification along with previous associations with PFAS toxicity mechanisms.

Vitamin D receptor (VDR) was most frequently identified pathway (54 out of possible 60 exposure profiles across 2 independent experiments for 30 substances) with a proportionally low mean BMC rank order of 54.6 out of 579 possible Wikipathway associations. Among PFAS, only PFNA was more potent than PFOA for VDR interactions with a mean BMC rank order of 57. Consistent with these findings, a recent study linked PFOA and other PFAS to competitive binding of VDR.^37^ Each of the 5 AFFF products resulted in VDR Wikipathway interactions, with AFFF2 producing the most potent response followed by AFFF1, AFFF4, AFFF5, and AFFF3. The AFFF-Qpool product had intermediate potency, while the S-550 product had a median BMC that was ∼4-fold lower than that of AFFF2. These data indicate that PFAS and AFFF exposures may have the potential to disrupt hepatic VDR pathways, and further investigation to mechanistically understand the potential health effects is warranted.

**Figure 2:**
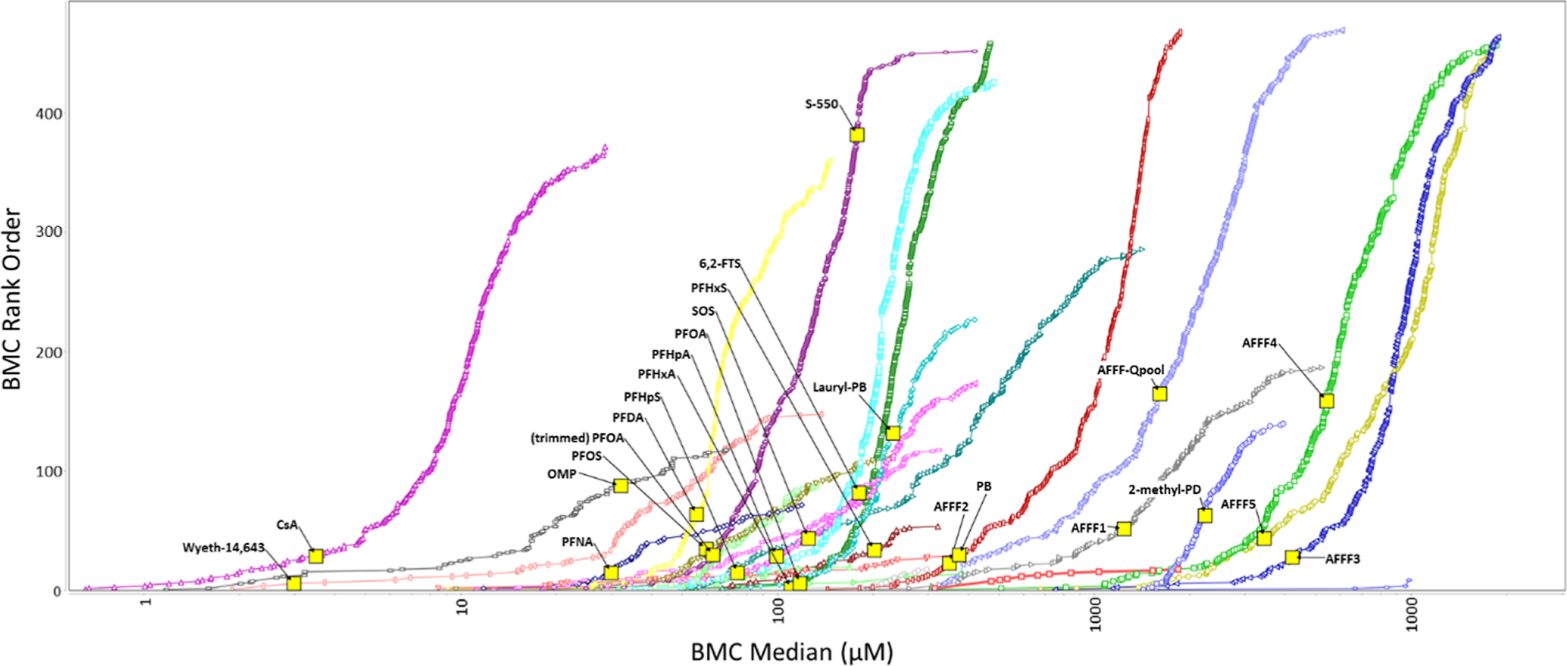
Accumulation plots for experimental Run1 highlighting PPARα pathway potencies (median gene in pathway) using Wikipathway (MSigDB7.2) gene sets. Comparable responses were observed in experimental Run2 (Supporting Information).

Numerous PFAS toxicity studies have suspected peroxisome proliferator-activated receptors (PPARs) as key signaling pathways for toxicity due to their role in lipid metabolism, and PPARα ranked among the top-20 most frequently identified pathways. Prototype agonist Wyeth-14,643 was chosen as a translational anchor for PPARα, which was both potent (∼8 μM) and effective (∼6× fold-change) to alter 14 PPARα target genes, as summarized in Figure 2 and Table 3. Cyclosporin A (CsA) was chosen as a reference chemical for fatty liver end points including PPARα and also potently altered PPARα target genes consistent with its extensive clinical associations to fatty liver disease. Overall, comparatively low BMC rank orders were observed for 23 substances (median BMC rank order of 34). Interestingly, terminally carboxylated PFAS (i.e., PFOA, PFDA, PFNA, PFHxA), as a structurally related subclass, were among the most biologically active for PPARα (i.e., proportionally lower BMCs, higher maximum fold-change), while perfluorinated sulfonates PFOS, PFHxS, and PFBS were markedly less effective (i.e., higher BMCs, lower maximum fold-change). This suggests a more prominent role for PPARα with perfluorinated carboxylate hepatocellular effects at more nascent stages of biological response. Each of the 7 AFFF-related products gave rise to marked maximum fold-change responses for PPARα (i.e., >10-fold, ranging from 11.5 to 339, Table 3). Most PPARα pathway responses appeared nonmonotonic with initial increases at lower exposure levels that gave rise to more profound decreases at higher AFFF exposures that appear consistent with cellular stress responses. However, AFFF2 and S-550 showed largely monotonic suppression responses across PPARα target genes. Diluted AFFF products were less potent than some individual PFAS (i.e., higher BMCs) using a suppositional average constituent molecular weight of 300 Da, which was consistent with their lower PFAS proportions. Among MIL-SPEC-qualified AFFFs, AFFF2 was most potent for PPARα target gene responses (∼290 μM) but less potent than the manufacturer-related S-550 PFAS mixture (∼116 μM). Laurylamidopropyl betaine, as well as detergent SOS, was a relatively potent non-PFAS AFFF constituent (∼250 μM). PFOS and 6,2-FTS led to modest rankings for PPARα response, while shorter chain PFBS and 4,2-FTS did not produce discernible PPARα target gene responses. In general, shorter chain PFAS ranked lower for PPARα, while carboxylates ranked higher, consistent with previous studies.^25,26,38^

**Table 3:** Prioritization of Test Substances for PPARα Interactions with Averaged BMC (μM), Fold-change, and Fold-change Divided by BMC Metrics across 2 Independent Experiments^a^^,^^c^

test substance name	mean BMC_Median_ (PPARα, μM)	max fold-change (PPARα, μM)	FOC/BMC × 100 (PPARα, μM)
CsA	2.92	23.6	808
Wyeth-14,643^b^	8.11	5.86	72.3
OMP	31.9	2.35	7.37
PFNA	49.2	6.59	13.4
PFOA-trimmed-monotonic	53.5	9.43	17.6
PFHpA	59.2	17.5	29.6
PFHpS	64.0	8.65	13.5
PFOA	97.1	17.3	17.8
PFHxA	98.9	5.08	5.14
S-550	116	260	224
PFOS	144	4.44	3.08
SOS	145	11.7	8.07
laurylamidopropyl betaine	159	6.34	3.99
6,2-FTS	170	5.52	3.25
PFDA	204	39.2	19.2
PFHxS	206	4.81	2.33
AFFF2	293	44.3	15.1
PB	320	2.21	0.691
AFFF1	1089	11.5	1.06
AFFF-Qpool	1143	106	9.27
2-methyl-2,4-pentanediol	1713	3.84	0.224
AFFF4	3581	339	9.47
AFFF5	3977	14.9	0.375
AFFF3	4583	82.2	1.79

a Substances sorted by mean BMC_Median,PPARα_.

b Postfilter addition of CYP4A11 to the gene set from Hill Model fit BMC in lieu of Poly2.

c FOC/BMC × 100 represents the calculated maximum fold-overcontrol response for each respective chemical divided by its mean BMC_Median_ value that is multiplied by 100 to enable intuitive value comparisons.

We next explored transcriptomic responses for the CAR pathway (Figure S5, Table S6). Phenobarbital (PB) served as a clinical reference drug for CAR activation and produced a robust induction for 13 out of 23 CAR target genes (Wikipathways, MSigDB7.2) at clinically relevant concentrations (*C*_max_ ∼ 120 μM). Analogous to PPARα, the relative potencies for the CAR pathway had comparatively low BMC rank orders across Wikipathways for a given test substance with a median across all substances of 16 (BMC ∼ 107 μM) out of 579 possible Wikipathway associations. PFOS had higher maximum fold change for CAR target genes (i.e., up to ∼212-fold) than the modest 4.5-fold maximum change for PPARα target genes and were also more potently sensitive (∼63 vs ∼144 μM). By comparison, PFOA was notably less effective for CAR relative to positive control responses. In general, perfluorinated sulfonates more closely resembled CAR-type activators than classical PPAR-type agonists, apart from PFHpS that appeared effective to modulate both pathways. PFOS showed distinctively higher mean fold-change for CAR modulation than topologically similar nonfluorinated detergent SOS (212 vs 17.8). All AFFF-related product mixtures, analogous to PPARα responses, were bioactive for CAR activation, with most revealing nonmonotonic initial increases in gene expression followed by suppression at higher exposures.

Lipid accumulation and fatty acid metabolism and transport have been extensively linked to PFAS toxicity. The Wikipathway for fatty acid omega oxidation was observed in 43 of 60 possible instances with comparatively low BMC rank orders (48.5 of 579 Wikipathways). The lowest BMC observed was 3.7 μM with CsA having an overall upward consistent adaptive response to impaired hepatic functionality. Wyeth-14,643 exposures produced a BMC = 20.4 μM with an overall down directionality. By contrast, PFOS exposures yielded an overall up directionality similar to CsA with a BMC = 46.7 μM and mean maximum fold-change of 211 that ranked second-highest among substances. PFOA was less potent with lower maximum fold-change averaging BMC = 170 μM (32-fold). Fluorotelomer 6,2-FTS yielded an intermediate BMC = 127 μM (∼25-fold-overcontrol). AFFF2, AFFF3, AFFF4, and AFFF5 exposures also resulted in a change for this pathway. Interestingly, AFFF1 was less responsive for this pathway and yielded a reversed directionality compared to other foam products (i.e., up vs down). This was consistent with the role of CAR in lipid metabolism that may reflect a more selective state of hepatocellular perturbation. Taken together, the totality of these molecular pathway response analyses supports emerging hypotheses linking PFAS to disruptions of fatty acid metabolism in humans, fills important PFAS data gaps, and introduces novel metrics for molecular hazard prioritization.

### Biological-Response Similarities

**Figure 3:**
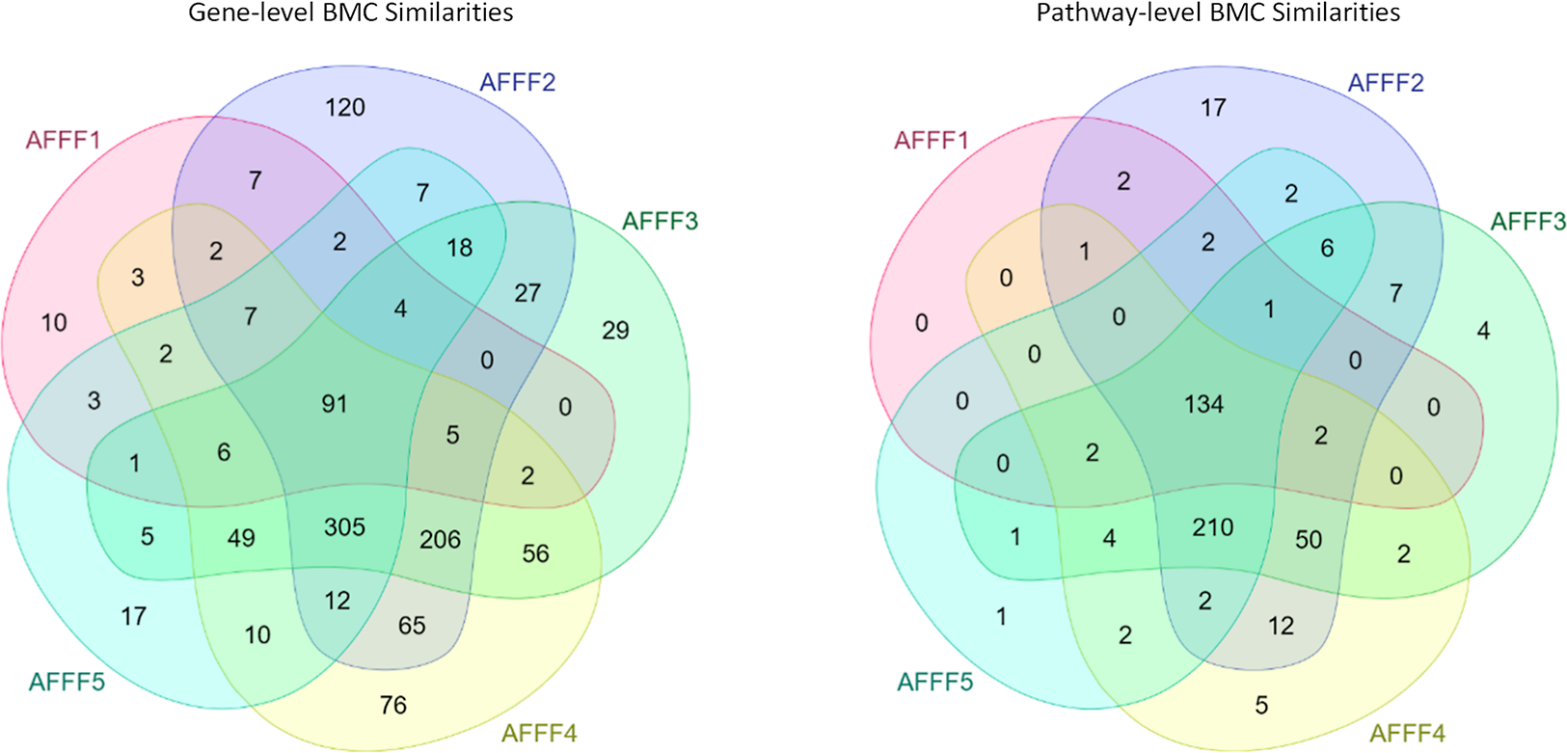
Venn diagram comparing the intersecting gene- and pathway-level transcriptomic response similarities for 5 MIL-SPEC-qualified AFFFs in human hepatocytes across 2 independent experiments.

**Figure 4:**
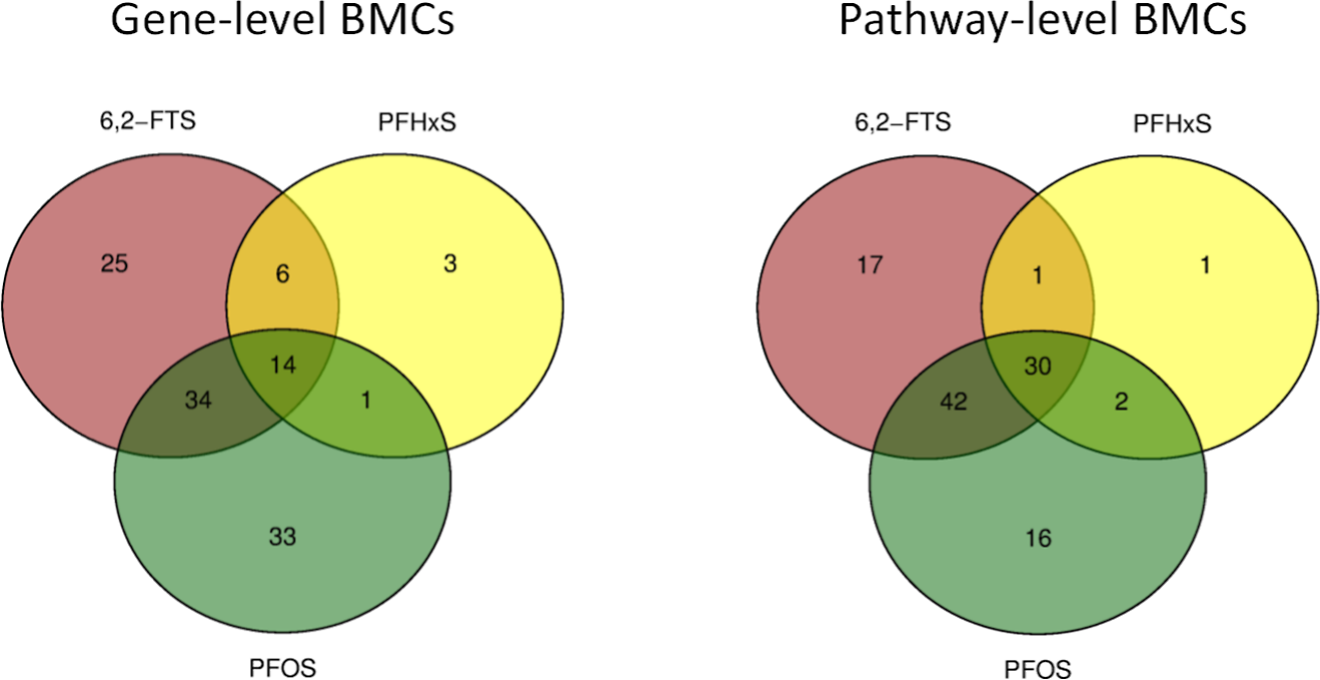
Venn diagram comparing the overlap of identified transcriptomic BMCs for PFOS, PFHxS, and 6,2-FTS at the gene- and pathway-level by their consensus (intersecting) responses across 2 independent experiments.

Thousands of PFAS are being identified and categorized into structural subclasses as the scientific community works to understand the emerging landscape of PFAS in our environment.^39^ Toxicological read-across relies upon these classifications to predict toxicity with data-poor substances. In this study, we explored approaches to characterize and understand biological response similarity with PFAS and AFFF mixtures, human hepatocyte transcriptomic data, and benchmark dose modeling. Initially, we tabulated the intersections of identified gene- and pathway-level BMCs (AFFF HTT uM BMCs-final.bm2) summarized in Figures 3 and 4 and Tables S7–S9. AFFF product mixtures shared 91 out of possible 145 gene-level BMCs (63%) and 134 of 144 pathway-level BMCs (93%), indicating a high degree of hepatocellular response similarity. AFFF2 appeared to be most distinct with 120 unique gene-level BMCs and 17 unique pathway-level BMCs, while AFFF1 had the most overlap with the other AFFFs. As expected, the AFFF-Qpool had a high degree of overlap with AFFF products (i.e., 64%–76%, gene; 91%–97%, pathway). The S-550 product (PFAS constituents + PEG) yielded 83% gene-level and 97% pathway-level similarity to manufacturer-related AFFF2, suggesting that similar types of PFAS constituents may drive the majority of hepatocellular response activity given the negligible responses observed with PEG. AFFF2 constituent laurylamidopropyl betaine yielded BMC overlaps of 59% (gene) and 99% (pathway) with AFFF2. However, this comprised only 15% of the AFFF2 gene-level BMCs. It was notable that fluorotelomer AFFF constituent 6,2-FTS had a distinctively high similarity to AFFF1 (49%, pathway-level), given that the highest measured hepatic levels of 6,2-FTS were with AFFF1 in a recent in vivo study of these same 5 AFFF products (manuscript in preparation). These molecular response data with complex AFFF product mixtures alongside constituents provide useful context for unraveling the hepatocellular effects of PFAS/AFFF in human hepatocytes. Further investigation to better understand mixture effects and constituent drivers of hepatocellular responses is warranted.

Extensive BMC overlap was observed among perfluorinated sulfonates PFOS, PFHxS, and the understudied fluorotelomer 6,2-FTS (Figure 4). These hepatocellular response similarities strengthen opportunities for near-term read across for liver effects. Furthermore, BMCs with shorter-chain PFBS, though fewer, overlapped 100% with this intersection. Detergent SOS produced BMCs with high overlap to structurally similar PFOS, which is generally considered safe. This highlights the importance of accounting for bioaccessibility in translation of in vitro response data that generally enable direct exposure to liver tissue compartments sans gut filtration.^35^

**Figure 5:**
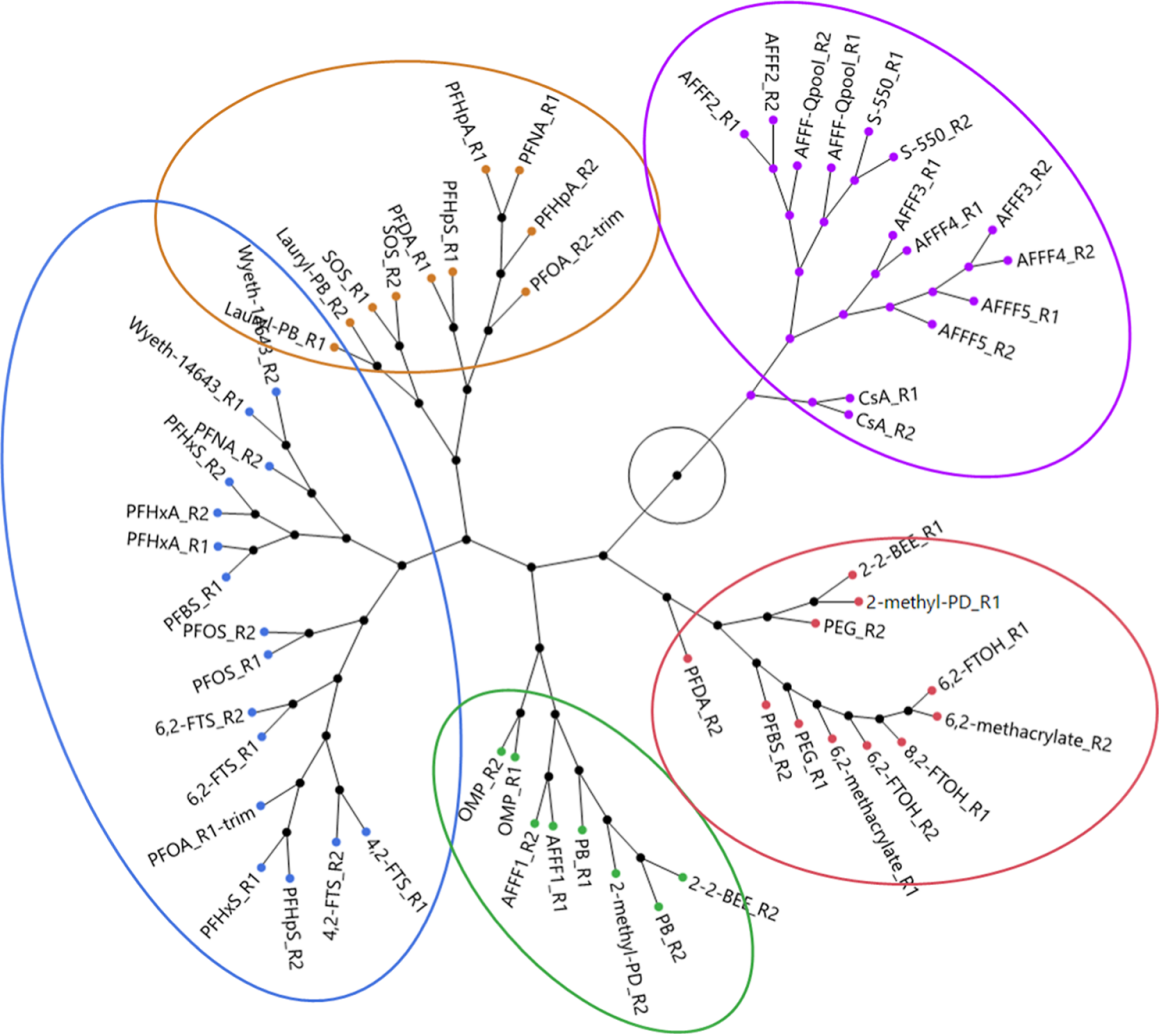
Constellation plot of hierarchical clustering of rank order scores for the top 100 most potent BMCs and top 100 BMCs with highest maximum fold-change gene-level BMCs, as described in Materials and Methods.

While BMC profile comparisons represent an intuitive approach to survey biological response similarities, chronic toxicity and reference dose effects are often driven by lower-dose effects that may be masked by higher-exposure effects (e.g., cellular stress). Thus, we developed a more global signature-based approach focusing on the 100 most potent BMCs and/or the top 100 maximum fold-change response BMCs, as described in Materials and Methods. As shown in Figure 5, there was generally good agreement in BMC signature profiles between independent experiments (i.e., R1 and R2). AFFF formulations largely clustered together (violet cluster) and were further subdivided by an AFFF2-containing branch characterized by more potently cytotoxic foams in human hepatocytes. Consistent with this observation, common low-dose gene sets for this cluster were enriched with Wikipathways related to cell cycle, cell proliferation, and cellular stress responses. AFFF1 subclustered distinctly from other AFFFs in closer proximity to the phenobarbital-anchored cluster (green). We hypothesize this could be the consequence of substantially higher measured levels of 6,2-FTS than other foams (manuscript in preparation), given its effective modulation of CAR target gene expression. In general, biological responses to AFFF were most similar to CsA, suggesting a potential for fatty liver disease and perhaps effects related to its therapeutic effects on immune suppression. We also investigated potency-only clustering (Figure S10) and observed detergents SOS and laurylamidopropyl betaine (lauryl-PB) clustered with AFFFs. Applying fold-change only clustering, these detergents shifted closer to the brown and green/blue clusters (Figures 5 and S11). Genes comprising substances in the brown cluster were enriched for PPAR, CAR, and lipid metabolism target genes and somewhat distinct from those in the blue/green clusters anchored by Wyeth-14,643 and PB. The red cluster was largely composed of weakly bioactive substances (e.g., PEG and 6,2-methacrylate, 6,2-FTOH). Initial clustering with PFOA revealed surprisingly low similarity to Wyeth-14,643 and closer proximity to the AFFF/CsA cluster (violet). However, closer inspection of PFOA data revealed a high proportion of nonmonotonic PFOA responses at the highest exposure level approaching cellular stress/death concentrations. Thus, we also compared a trimmed PFOA to remove the highest exposure level and better reflect transcriptomic points of departure at more nascent stages of hepatocellular response. This markedly shifted PFOA to be more aligned with Wyeth-14,643 and other carboxylates. Overall, this integrated approach represents a data-driven framework to characterize and unravel biological response similarities and support read-across efforts with PFAS.

#### In Vivo Translation of Transcriptomic Responses to PFAS Exposures

**Figure 6:**
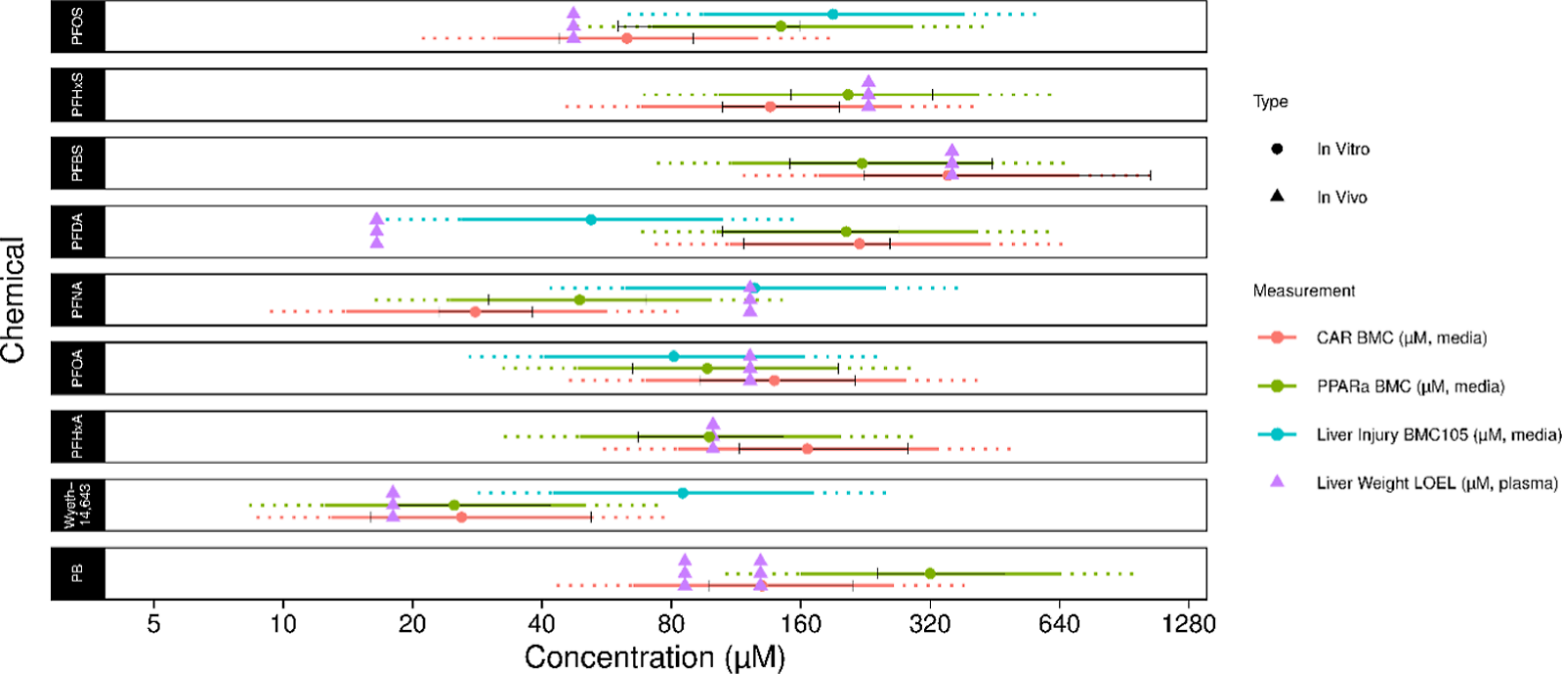
Comparison of in vivo plasma concentrations at the liver weight LOEL (violet triangles) from National Toxicology Program 28 day studies to in vitro human hepatocyte transcriptomic pathway potencies (nominal) with established causal associations to liver weight change. Here, red circles indicate mean in vitro CAR BMC_Median_ values with solid red lines reflecting the 2-fold potency range, dotted red lines reflecting the 3-fold potency range, and vertical line markers reflecting the statistically derived BMCL and BMCU boundary values for the CAR and PPARα pathways over independent experiments (summarized BMC ranges for each experiment provided in Table S13).

The National Toxicology Program (NTP) has previously reported 28 day in vivo toxicology studies with Harland Sprague-Dawley rats exposed to perfluorinated sulfonates (PFOS, PFHxS, and PFBS)^25^ and carboxylates (PFDA, PFNA, PFOA, PFHxA),^26^ revealing a range of biological effects that included liver weight change and corresponding blood plasma PFAS concentrations. Given the established role for PPARα and CAR as molecular initiating events for hepatomegaly, along with liver injury, we explored the hypothesis that pathway-level BMCs from these in vitro hepatocyte transcriptomic data could effectively estimate the corresponding in vivo liver weight change potency ranges using an internal dose (i.e., blood plasma concentrations). As summarized in Figure 6, mean interexperiment potency ranges for PPARα, CAR, and BMC105 reasonably approximated the potency ranges for hepatomegaly observed in 28 day in vivo rat studies (i.e., within 2- to 3-fold). Here, solid lines reflect 2-fold potency windows around each pathway BMC, dotted lines reflect 3-fold potency windows, and vertical lines for transcriptomic potency ranges reflect statistically derived lower bound BMC (BMCL) and upper bound BMC (BMCU) across independent experiments. Tables S12 and S13 and downloadable export “Final export of identified pathways (3).xlsx” provide detailed summaries of these data. Prototype PPARα agonist Wyeth-14,643, established to serve a causal role in rodent hepatomegaly, gave rise to a mean in vitro PPARα BMC (i.e., BMC_PPARα_) of ∼25 μM, which was comparable to reported in vivo plasma concentration ranges (violet triangles) at the liver weight LOEL (i.e., LOEL_PLASMA_) using linear extrapolation from NTP-TR-096^25^ (i.e., 6.25 mg/kg-day using low-dose toxicokinetics reported in TOX-62^40^). A similar potency range concordance was observed for phenobarbital (PB), a prototype CAR activator, and reported human therapeutic peak plasma concentration ranges.^41,42^ Human ranges were used for PB due to known species differences for PB activation of CAR.^43^ These responses with drug substances in combination with proven regulatory utility to predict the potential for clinical drug–drug interactions with differentiated human hepatocytes and CYP450 gene expression support extension of this approach with PFAS substances.

PFOS markedly activated CAR target gene expression in both human hepatocytes and rat liver. Comparison of potency ranges for BMC_CAR_ (∼63 μM) to PFOS-induced rat liver weight LOEL_PLASMA_ (∼47.4 μM) revealed closer alignment with BMC_CAR_ than BMC_PPARα_ (Figure 6), which was consistent with fold-change response similarities. A similar potency overlap was observed for shorter-chain sulfonate PFHxS (BMC_CAR_ = 136 μM vs LOEL_PLASMA_ ∼ 230 μM), which also overlapped closely with BMC_PPARα_ ∼ 206 μM. For short-chain sulfonate PFBS, although fewer transcriptomic responses were observed, it was also bioactive for CAR. Initial comparison of BMC_CAR_ = 360 μM to LOEL_PLASMA_ ∼ 17.9 μM revealed a marked 18-fold difference. However, on day 28, rat plasma was collected 24 h post exposure. Accounting for PFBS shorter half-life (hours), using linear scaling from previous US-EPA study that reported 95% of PFBS was cleared from rat plasma after 24 h,^23^ 2-fold concordance was again observed (BMC_CAR_ and BMC_PPARα_).

For carboxylates, the LOEL_PLASMA_ ∼ 122 μM for PFOA was within 2-fold of BMC_PPARα_ = 97 μM and consistent with the reported in vivo induction of Cyp4a1.^26^ A similar potency overlap was observed with PFNA that was within 3-fold of BMC_PPARα_ = 49 μM. However, longer-chain carboxylates PFNA and PFDA appeared more closely aligned with their respective BMC105 liver injury thresholds than either CAR or PPARα and consistent with their comparatively high rankings for liver injury. PFHxA, similar to PFBS, has a shorter half-life (2.5 h), and linear extrapolation of LOEL_PLASMA_ revealed ∼100 μM that was best approximated by BMC_PPARα_ = 98 μM and also overlapped within 2-fold of BMC_CAR_ = 166 μM.

Finally, we applied this novel translational approach to fill a present-day data gap for 6,2-FTS. Based on human hepatocyte transcriptomic data, 6,2-FTS most closely resembled a CAR-type activator with a more potent and maximal fold-change response for the CAR pathway (∼80.1 μM, ∼25-fold) than PPARα (∼170 μM, ∼5-fold). Thus, we predict that future studies will measure the internal plasma concentrations of 6,2-FTS at the rat liver weight LOEL to be within 2–3-fold of ∼80 μM.

Overall, this focused and intuitive extension of hepatocyte transcriptomics data to predict in vivo potency ranges represents one of many potential methods for creating translational “threads” that weave together into a more complete integration of mechanistic data to predict human health effects. We acknowledge that the defined 2–3-fold potency window concordance may be limiting, but represents a fit-for-purpose approach to initially approximate in vivo toxicological potency ranges with prequalified NAMs and has been successfully applied to predict drug–drug interactions in humans. Ultimately, predictive utility will be predicted on sufficient in vitro modeling of in vivo pathophysiology and interspecies similarity, which appears to be largely conserved for the 7 perfluorinated PFAS in this study and strengthens the extension of this approach to 6,2-FTS and other substances going forward.

In conclusion, this study addresses important mechanistic and human health data gaps with AFFF and PFAS while introducing scalable strategies to address the global challenges that PFAS pose to our environment. The impact of this work builds confidence with physiologically relevant human liver models for translational toxicology screening, molecular hazard prioritization, and biological-response similarities that proactively inform read across and represent a continued evolution of tools for greener chemistries and next-generation risk assessments.

## Data Availability

Publicly available data files and folders (e.g., raw, processed, analyzed) can be accessed at 10.22427/NTP-DATA-500-017-001-000-4.

## Supporting Information Available

Test substance procurement summary; summary of ATP depletion potencies; AFFF ATP depletion comparison; percent (%) dilution to μM unit conversions applied; accumulation plots for experimental Run1 highlighting CAR pathway potencies (median gene within pathway) for each test substance using Wikipathway annotations; prioritization of test substances for CAR interactions with averaged μM BMC fold-change and fold-change divided by BMC metrics across 2 independent experiments; gene-level BMC similarities for the intersection of independent experiments; pathway-level BMC similarities for the intersection of independent experiments; Jaccard index comparisons for biological response similarities for the intersecting gene- and pathway-level transcriptomic BMCs across independent experiments from AFFF product exposures to human hepatocytes; potency-only constellation plot of hierarchical clustering of rank scores for the top 100 most potent gene-level BMCs from each of the 60 experimental runs (30 test substances x 2 runs); fold-change-only constellation plot of hierarchical clustering of rank scores for the top 100 most efficacious (fold-change) gene-level BMCs from each of the 60 experimental runs (30 test substances x 2 runs); comparison of in vivo LOEL values to in vitro potencies for CAR, PPARα, and liver injury; and summary of CAR and PPARα BMC values including statistically derived upper and lower bounds (PDF)
Benchmark concentration analysis in units of % dilution (v/v): “AFFF HTT % BMCs-final.bm2” (ZIP)
Benchmark concentration analysis in units of μM: “AFFF HTT uM BMCs-final.bm2” (ZIP)
Liver injury classifications gene-level BMC modeling in units of μM: “AFFF HTT uM BMCs-final-shorter labels-Gene Level SILI.bm2” (ZIP)
Liver injury classifications gene-level BMC modeling in units of % dilution (v/v): “AFFF-BMCs-%-R1&2-Unfiltered cytotox-stress for liver injury thresholds.bm2” (ZIP)
Summary of liver injury thresholds (i.e., BMC105 values): “BMC105 Rankings-final-3.xlsx” (XLSX)
Complete list of filtered pathway-level BMCs in units of μM “Final export of identified pathways (3).xlsx” (XLSX)
ATP depletion analysis: “ATP Depletion Analyzed Data.zip” (ZIP)
Folder of deep learning analysis of cell morphologies: “Cell Morphology Image Analysis-Sciome.zip” (ZIP)
Liver enzyme leakage (LDH) analysis: “BMD_Analysis_AFFF_LDH_Run1.bm2”; “BMD_Analysis_AFFF_LDH_Run2.bm2” (ZIP)

## Abbreviations

HTT: high-throughput transcriptomics
PPAR: peroxisome proliferator-activated receptor
CAR: constitutive androstane receptor
BMC: benchmark concentration
LOEL: lowest observable effect level
PFAS: per- and poly fluorinated alkyl substance
AFFF: aqueous film-forming foam
