# Supplementary material for: Unraveling Human Hepatocellular Responses to PFAS and Aqueous Film-Forming Foams (AFFFs) for Molecular Hazard Prioritization and In Vivo Translation
Source: Environ Sci Technol. 2025 Feb 2;59(5):2423–35. doi: 10.1021/acs.est.4c10595 (PMC11823446; doi:10.1021/acs.est.4c10595)
Supplement: Supplementary file 1 — es4c10595_si_001.pdf [file es4c10595_si_001.pdf]

Unraveling human hepatocellular responses to PFAS and aqueous film-forming foams (AFFFs) for molecular hazard prioritization and in vivo translation

Kevin A. Mauge-Lewis[a,‡], Sreenivasa C. Ramaiahgari[a,‡,#], Scott S. Auerbach[a], Georgia K. Roberts[a], Suramya Waidyanatha[a], Suzanne E Fenton[a,†], Dhiral P. Phadke[b], Michele R. Balik-Meisner[b], Arpit Tandon[b], Deepak Mav[b], Brian Howard[b], Ruchir Shah[b], Barney Sparrow[c], Jenni Gorospe[c], Stephen S. Ferguson[a,*]

[a]Division of Translational Toxicology, National Institute for Environmental Sciences, 111 TW Alexander Drive, Research Triangle Park, NC 27709, United States
[b]Sciome, 1920 NC-54 Suite 510 & 520, Durham, NC 27713, United States
[c]Battelle, 505 King Ave, Columbus, OH 43201, United States
*Email: stephen.ferguson@nih.gov; (984) 287 3128
‡K.A.M. and S.C.R. contributed equally to this work
Present Addresses:

#S.C.R.: United States Environmental Protection Agency, Research Triangle Park, NC 27709, United States

†S.E.F.: NC State University, Raleigh, NC 27606, United States

Corresponding Author:
Stephen Ferguson
Mechanistic Toxicology Branch
Division of Translational Toxicology
National Institute of Environmental Health Sciences
111 TW Alexander Dr.
Building 101, Room E140B
P.O. Box 12233
Research Triangle Park, NC 27709
Email: stephen.ferguson@nih.gov; (984) 287 3128

Number of Pages: 15

Number of Figures: 5

Number of Tables: 8

Supplemental Data

Supplemental Table S1: Test Substance Procurement Summary

Chemical/Product Name	CASRN	Manufacturer	Product/Item Number	Lot/Batch Number	Substance Abbreviation
Angus Fire Tridol® Mc6 3% AFFF	AFFF	Angus Fire (Angier NC)	3133-8340-4	L17081AN	AFFF3
Solberg ArcticTM 3% MIL-SPEC AFFF	AFFF	The Solberg Company (Green Bay WI)	20770	AA1600110	AFFF1
Chemguard 3% AFFF (C306-MS-C)	AFFF	Tyco Fire Protection Products (Lansdale PA)	770809	MS31716	AFFF2
Phos-Chek 3% AFFF MILSPEC	AFFF	Perimeter Solutions (Rancho Cucamonga CA)	10004164	249190702	AFFF4
Fomtec AFFF 3% M	AFFF	Dafo Fomtec AB (Tyresö Sweden)	10-3040-01	190802	AFFF5
AFFF-Qpool	AFFF Mixture	Equal volume mixture of AFFF product numbers: 3133-8340-4, 20770, 770809, 10004164, 10-3040-01	Equal volume mixture of products	Equal volume mixture of products	AFFF-Qpool
CHEMGUARD S-550 (PFAS Substances+PEG)	N/A	Tyco Fire Protection Products (Lansdale PA)	11W550	780076120419	S-550
perfluorooctane sulfonate	1763-23-1	Matrix Scientific	3767	T20G	PFOS
perfluoroheptane sulfonate	375-92-8	SynQuest Laboratories	6164-3-2S	341800	PFHpS
perfluorohexane sulfonate (potassium salt)	3871-99-6	Interchim (France)	18236	230002	PFHxS
perfluorobutane sulfonate	375-73-5	Sigma-Aldrich	562629	15414TE	PFBS
perfluorodecanoic acid	335-76-2	Sigma-Aldrich	177741	01820LE	PFDA
perfluorononanoic acid	375-95-1	Oakwood Products	2263	D11G	PFNA
perfluorooctanoic acid	335-67-1	Sigma-Aldrich	17146-8	WXBD1156V	PFOA
perfluoroheptanoic acid	375-85-9	Apollo Scientific	PC6034	AS480225	PFHpA
perfluorohexanoic acid	307-24-4	Matrix Scientific	3945	Q02G	PFHxA
6,2-fluorotelomer sulfonate	27619-97-2	SynQuest Laboratories	6164-3-06	512700	6,2-FTS
4,2-fluorotelomer sulfonate	757124-72-4	SynQuest Laboratories	6164-3-38	553600	4,2-FTS
6,2-methacrylate	2144-53-8	Sigma-Aldrich	474215	MKCG7038	6,2-methacrylate
6,2-fluorotelomer octanol	647-42-7	Sigma-Aldrich	370533	MKCL9941	6,2-FTOH
8,2-fluorotelomer decanol	678-39-7	Sigma-Aldrich	09115HE	532789	8,2-FTOH
sodium octyl sulfate	142-31-4	Sigma-Aldrich	O4003	SLBZ9589	SOS
poly(ethylene glycol)	25322-68-3	Sigma-Aldrich	202401	BCCC3149	PEG
diethylene glycol butyl ether	112-34-5	Sigma-Aldrich	579963	WXBD1763V	2-(2-Butoxyethoxy)ethanol
lauroylamide propylbetaine	4292-10-8	Santa Cruz Biotechnology	sc-488785	I2816	laurylamidopropyl betaine
hexylene glycol	107-41-5	Sigma-Aldrich	112100	BCCC4178	2-methyl-2,4-pentanediol
omeprazole	73590-58-6	Sigma-Aldrich	O104	BCCB5788	OMP
phenobarbital (sodium salt)	57-30-7	Sigma-Aldrich	P5178	SLCD9096	PB
wyeth-14643	50892-23-4	A.G. Scientific, Inc.	W-1026	C1204	WY-14643
cyclosporin A	59865-13-3	TCI America	C2408	4244F	CsA

Exposure Range-finding

Initial exposure range-finding was conducted using ATP depletion assays to infer loss of cell viability (i.e., CellTiter-Glo, Promega, Madison WI) after 96-hour exposures as described previously [1]. Exposures were initiated ~72 hours after cell seeding/differentiation, and exposure media were exchanged after 48 hours. Cell cultures were washed twice with phosphate-buffered saline (PBS) and CellTiter-Glo® assays were performed according to the manufacturer's protocol. Cell culture plates were assayed in a CLARIOstar Microplate Reader (BMG Labtech, Cary, NC, SN: 430-0277, Software Version 5.61). Raw luminescence data were analyzed in BMD Express 2.3 to derive potencies for hepatocellular viability loss. Here, raw luminescence data were log(2) transformed and imported. William's trend test filtering using a P-Value of 0.05 and fold-change filter of 1.2 were applied. Benchmark concentration values were derived using the Hill and Poly2 models with confidence level of 0.95 (constant variance), BMR = 1 SD, and P-Value Cutoff of 0.05. The resulting data analysis is provided in "ATP Depletion AFFF study-corrected.bm2" with supplemental data files.

LDH Leakage Data Analysis

Luminescence intensity was measured using a CLARIOstar microplate reader (BMG Labtech, Cary, NC). Raw luminescence data were normalized to plate-matched vehicle controls and analyzed in BMDExpress 2.3. Here, normalized LDH leakage data were log2 transformed and filtered with a William's trend test at $P<0.05$ and 1.2-fold over control. Finally, BMCs were derived using a BMR of 2 SD. BMC values were tabulated for comparisons to cell morphology and transcriptomic thresholds. Aflatoxin B1 exposures (10 µM) were included as a positive control.

Supplemental Table S2: Summary of ATP Depletion Potencies

Test Substance	Exposure Range (%)	Exposure Range (µM)	ATP Depletion BMC (% v/v)	Fold Change ATP Depletion
S-550	0.00006 - 0.6		0.00687	207
AFFF-Qpool	0.00006 - 0.6		0.0789	147
AFFF2	0.00006 - 0.6		0.0230	107
Laurylamidopropyl betaine	0.00002 - 0.2		0.0132	103
SOS	0.00002 - 0.2		0.0289	20.5
PFOA		0.05 - 500	0.00555	3.09
AFFF3	0.00006 - 0.6		0.0725	2.33
PFHpS		0.05 - 500	0.00605	2.23
AFFF4	0.00006 - 0.6		0.0515	1.79
2-methyl-2,4-pentanediol	0.00002 - 0.2		0.00339	1.73
AFFF5	0.00006 - 0.6		0.115	1.67
AFFF1	0.00006 - 0.6		0.00960	1.58
PFNA		0.0125 -125	0.00173	1.51
6,2-FTS (6,2-FTSA)		0.05 - 500	0.00614	1.20
2-(2-Butoxyethoxy)ethanol	0.000006 - 0.06		N/A	
4,2-FTS (4,2-FTSA)		0.05 - 500	N/A	
6,2-FTOH		0.05 - 500	N/A	
6,2-methacrylate		0.05 - 500	N/A	
8,2-FTOH		0.05 - 500	N/A	
CsA		0.003 - 30	N/A	
OMP		0.01 - 100	N/A	
PB		0.1 - 1000	N/A	
PEG	0.00002 - 0.2		N/A	
PFBS		0.05 - 500	N/A	
PFDA		0.0125 - 125	N/A	
PFHpA		0.05 - 500	N/A	
PFHxS		0.05 - 500	N/A	
PFHxA		0.05 - 500	N/A	
PFOS		0.025 - 250	N/A	
Wyeth-14,643		0.02 - 200	N/A	

PFOS estimated BMC ~250µM based on orthogonal datasets.
N/A: Not applicable, no loss of viability observed.
*Average molecular weight of 300Da used for mixtures.

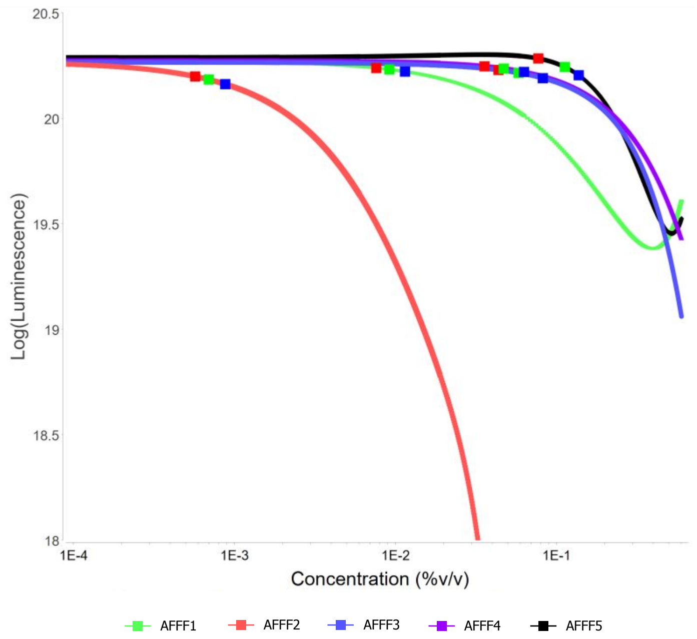

Supplemental Figure S3: AFFF ATP depletion comparison.

Supplemental Table S4: Percent (%) Dilution to µM Unit Conversions Applied

New Name	Units	Dose Group A	Dose Group B	Dose Group C	Dose Group D	Dose Group E	Dose Group F	Dose Group G	Dose Group H	Dose Group I	Description	Conversion factor
S-550	µM	6.666E+02	2.109E+02	6.676E+01	2.113E+01	6.685E+00	2.116E+00	6.695E-01	2.119E-01	6.705E-02	Average molecular weight of 300 Da	33330
	% (v/v)	2.000E-02	6.329E-03	2.003E-03	6.338E-04	2.006E-04	6.347E-05	2.009E-05	6.357E-06	2.012E-06	Nominal concentration	
AFFF-Qpool	µM	6.666E+03	2.109E+03	6.676E+02	2.113E+02	6.685E+01	2.116E+01	6.695E+00	2.119E+00	6.705E-01	Average molecular weight of 300 Da	33330
	% (v/v)	2.000E-01	6.329E-02	2.003E-02	6.338E-03	2.006E-03	6.347E-04	2.009E-04	6.357E-05	2.012E-05	Nominal concentration	
AFFF1	µM	6.666E+03	2.109E+03	6.676E+02	2.113E+02	6.685E+01	2.116E+01	6.695E+00	2.119E+00	6.705E-01	Average molecular weight of 300 Da	33330
	% (v/v)	2.000E-01	6.329E-02	2.003E-02	6.338E-03	2.006E-03	6.347E-04	2.009E-04	6.357E-05	2.012E-05	Nominal concentration	
AFFF2	µM	2.000E+03	6.329E+02	2.003E+02	6.338E+01	2.006E+01	6.347E+00	2.009E+00	6.357E-01	2.012E-01	Average molecular weight of 300 Da	33333.33333
	% (v/v)	6.000E-02	1.899E-02	6.009E-03	1.901E-03	6.017E-04	1.904E-04	6.026E-05	1.907E-05	6.035E-06	Nominal concentration	
AFFF3	µM	2.000E+04	6.329E+03	2.003E+03	6.338E+02	2.006E+02	6.347E+01	2.009E+01	6.357E+00	2.012E+00	Average molecular weight of 300 Da	33333.33333
	% (v/v)	6.000E-01	1.899E-01	6.009E-02	1.901E-02	6.017E-03	1.904E-03	6.026E-04	1.907E-04	6.035E-05	Nominal concentration	
AFFF4	µM	2.000E+04	6.329E+03	2.003E+03	6.338E+02	2.006E+02	6.347E+01	2.009E+01	6.357E+00	2.012E+00	Average molecular weight of 300 Da	33333.33333
	% (v/v)	6.000E-01	1.899E-01	6.009E-02	1.901E-02	6.017E-03	1.904E-03	6.026E-04	1.907E-04	6.035E-05	Nominal concentration	
AFFF5	µM	2.000E+04	6.329E+03	2.003E+03	6.338E+02	2.006E+02	6.347E+01	2.009E+01	6.357E+00	2.012E+00	Average molecular weight of 300 Da	33333.33333
	% (v/v)	6.000E-01	1.899E-01	6.009E-02	1.901E-02	6.017E-03	1.904E-03	6.026E-04	1.907E-04	6.035E-05	Nominal concentration	

*Solution densities assumed to be 1 g/mL for exposure media largely comprised of water.

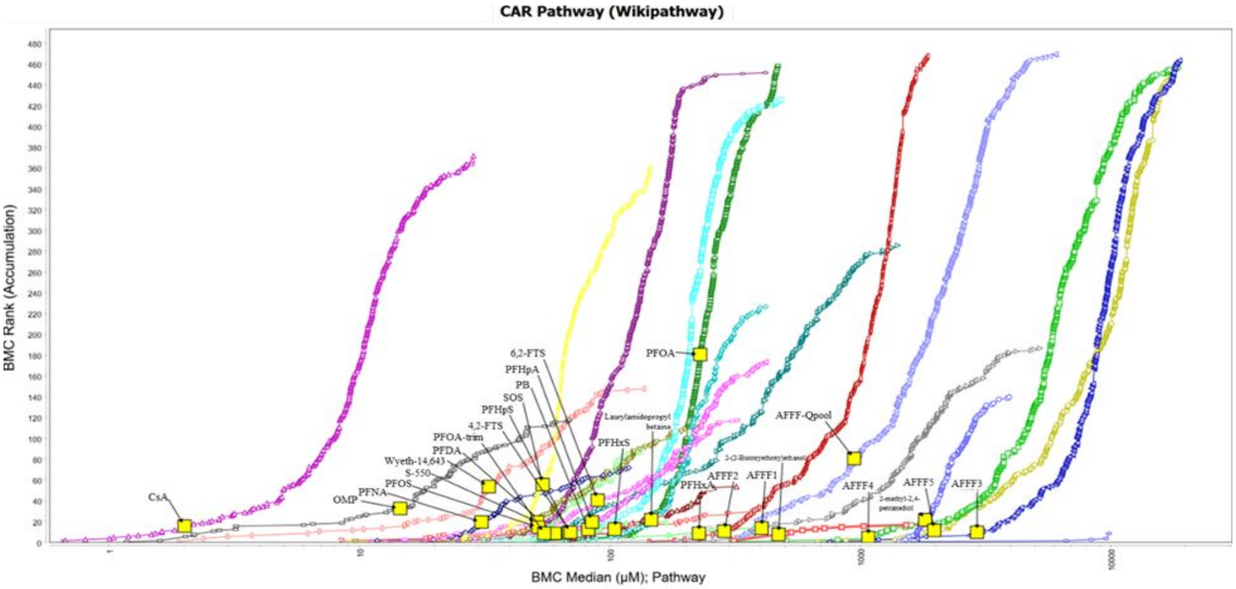

Supplemental Figure S5: Accumulation plots for experimental Run 1 highlighting CAR pathway potencies (median gene within pathway) for each test substance using Wikipathway annotations. Comparable responses were observed in experimental Run 2, as detailed in Supplemental Data files and information.

Supplemental Table S6: Prioritization of test substances for CAR interactions with averaged µM BMC, fold-change, and fold-change divided by BMC metrics across 2 independent experiments.

Test Substance Name	Mean BMC_{Median} (CAR, µM)	Max Fold Change (CAR, µM)	FOC/BMC*100 (CAR, µM)
OMP	13.1	248.2	1889
CsA	2.26	23.6	1043
PFOS	62.8	212	337
Wyeth-14,643	26.1	73.6	282
PB	130	327	253
PFHpA	70.6	148	210
S-550	37.3	46.3	124
PFNA	28.5	34.8	122
PFHpS	50.9	46.7	91.6
Laurylamidopropyl betaine	127	79.9	63.0
PFOA-trimmed-monotonic	52.0	32.4	62.2
PFHxS	136	51.0	37.5
6,2-FTS	80.1	25.5	31.8
PFOA	139	27.3	19.6
PFDA	219	30.9	14.1
SOS	128	17.8	13.9
AFFF2	266	32.3	12.2
AFFF1	495	52.9	10.7
AFFF-Qpool	709	37.6	5.3
AFFF4	1046	44.9	4.3
PFHxA	167	5.6	3.4
2-methyl-2,4-pentanediol	1496	26.1	1.7
AFFF3	1963	27.6	1.4
AFFF5	2323	29.3	1.3

Note: FOC/BMC*100 represents the calculated maximum fold-over-control response for each respective chemical divided by it Mean BMC_{Median} value that is multiplied by 100 to enable intuitive value comparisons.

Biological Response Similarities

The intersection of BMCs observed for experimental Run 1 and Run2 were tabulated for gene- and pathway-level BMCs as shown in Supplemental Tables 5 and 6, respectively.

Supplemental Table S7: Gene-level BMC similarities for the intersection of independent experiments.

Row	2-(2-Butoxyethoxy)ethanol_INTERSECTION-%-gene (14)	2-methyl-2,4-pentanediol_INTERSECTION-%-gene (23)	4,2-FTS_INTERSECTION-%-gene (10)	6,2-FTOH_INTERSECTION-%-gene (0)	6,2-FTS_INTERSECTION-%-gene (79)	8,2-FTOH_INTERSECTION-%-gene (0)	AFFF-Qpool_INTERSECTION-%-gene (727)	AFFF1-Solberg_INTERSECTION-%-gene (145)	AFFF2-Chemguard_INTERSECTION-%-gene (878)	AFFF3-Tridol_INTERSECTION-%-gene (804)	AFFF4-Phos-Chek_INTERSECTION-%-gene (897)	AFFF5-Fomtec_INTERSECTION-%-gene (539)	CsA_INTERSECTION-%-gene (518)	Laurylamidopropyl betaine_INTERSECTION-%-gene (226)	OMP_INTERSECTION-%-gene (78)	PB_INTERSECTION-%-gene (24)	PEG_INTERSECTION-%-gene (1)	PFBS_INTERSECTION-%-gene (3)	PFDA_INTERSECTION-%-gene (386)	PFHpA_INTERSECTION-%-gene (168)	PFHpS_INTERSECTION-%-gene (45)	PFHxA_INTERSECTION-%-gene (8)	PFHxS_INTERSECTION-%-gene (24)	PFNA_INTERSECTION-%-gene (20)	PFOA_INTERSECTION-%-gene (872)	PFOS_INTERSECTION-%-gene (82)	S-550_INTERSECTION-%-gene (976)	SOS_INTERSECTION-%-gene (246)	Wyeth-14643_INTERSECTION-%-gene (66)
2-(2-Butoxyethoxy)ethanol_INTERSECTION-%-gene (14)	14	5	8	0	11	0	6	14	14	7	7	9	8	14	11	11	0	1	11	14	11	5	11	7	13	10	14	14	12
2-methyl-2,4-pentanediol_INTERSECTION-%-gene (23)	5	23	3	0	12	0	20	17	21	20	21	21	20	18	13	7	1	1	17	21	12	2	6	6	15	12	22	16	10
4,2-FTS_INTERSECTION-%-gene (10)	8	3	10	0	8	0	4	10	10	6	5	6	5	8	8	9	0	2	6	10	10	4	9	6	10	6	10	9	9
6,2-FTOH_INTERSECTION-%-gene (0)	0	0	0	0	0	0	0	0	0	0	0	0	0	0	0	0	0	0	0	0	0	0	0	0	0	0	0	0	0
6,2-FTS_INTERSECTION-%-gene (79)	11	12	8	0	79	0	45	40	60	50	53	48	49	50	27	15	1	3	44	54	34	8	20	17	49	48	62	54	32
8,2-FTOH_INTERSECTION-%-gene (0)	0	0	0	0	0	0	0	0	0	0	0	0	0	0	0	0	0	0	0	0	0	0	0	0	0	0	0	0	0
AFFF-Qpool_INTERSECTION-%-gene (727)	6	20	4	0	45	0	727	93	628	595	632	409	340	111	45	13	1	0	282	111	27	2	11	10	513	46	663	161	38
AFFF1-Solberg_INTERSECTION-%-gene (145)	14	17	10	0	40	0	93	145	118	109	118	116	99	95	44	20	1	3	62	62	34	7	21	15	98	47	125	92	44
AFFF2-Chemguard_INTERSECTION-%-gene (878)	14	21	10	0	60	0	628	118	878	656	693	446	391	134	60	19	1	3	338	141	39	8	23	20	603	63	731	184	54
AFFF3-Tridol_INTERSECTION-%-gene (804)	7	20	6	0	50	0	595	109	656	804	720	479	395	137	48	13	1	3	314	123	30	4	15	14	540	57	705	184	44
AFFF4-Phos-Chek_INTERSECTION-%-gene (897)	7	21	5	0	53	0	632	118	693	720	897	482	412	149	53	15	1	2	318	129	31	3	12	12	601	62	762	197	52
AFFF5-Fomtec_INTERSECTION-%-gene (539)	9	21	6	0	48	0	409	116	446	479	482	539	323	138	54	17	1	2	246	125	30	5	16	14	341	56	485	180	47
CsA_INTERSECTION-%-gene (518)	8	20	5	0	49	0	340	99	391	395	412	323	518	127	48	11	1	2	214	116	29	5	14	15	331	48	425	161	42
Laurylamidopropyl betaine_INTERSECTION-%-gene (226)	14	18	8	0	50	0	111	95	134	137	149	138	127	226	40	15	1	1	89	83	33	6	17	16	115	51	168	106	40
OMP_INTERSECTION-%-gene (78)	11	13	8	0	27	0	45	44	60	48	53	54	48	40	78	18	1	1	45	50	25	3	13	10	49	34	64	43	29
PB_INTERSECTION-%-gene (24)	11	7	9	0	15	0	13	20	19	13	15	17	11	15	18	24	0	1	16	22	15	4	11	6	17	13	20	18	15
PEG_INTERSECTION-%-gene (1)	0	1	0	0	1	0	1	1	1	1	1	1	1	1	1	0	1	0	0	1	0	0	0	0	0	1	1	1	1
PFBS_INTERSECTION-%-gene (3)	1	1	2	0	3	0	0	3	3	3	2	2	2	1	1	1	0	3	2	3	3	2	3	3	3	3	2	3	3
PFDA_INTERSECTION-%-gene (386)	11	17	6	0	44	0	282	62	338	314	318	246	214	89	45	16	0	2	386	122	29	4	15	14	313	57	347	114	40
PFHpA_INTERSECTION-%-gene (168)	14	21	10	0	54	0	111	62	141	123	129	125	116	83	50	22	1	3	122	168	36	8	20	20	115	57	142	96	47
PFHpS_INTERSECTION-%-gene (45)	11	12	10	0	34	0	27	34	39	30	31	30	29	33	25	15	0	3	29	36	45	8	22	15	33	32	40	34	32
PFHxA_INTERSECTION-%-gene (8)	5	2	4	0	8	0	2	7	8	4	3	5	5	6	3	4	0	2	4	8	8	8	7	7	8	6	8	8	8
PFHxS_INTERSECTION-%-gene (24)	11	6	9	0	20	0	11	21	23	15	12	16	14	17	13	11	0	3	15	20	22	7	24	13	20	15	21	19	19
PFNA_INTERSECTION-%-gene (20)	7	6	6	0	17	0	10	15	20	14	12	14	15	16	10	6	0	3	14	20	15	7	13	20	16	16	18	17	17
PFOA_INTERSECTION-%-gene (872)	13	15	10	0	49	0	513	98	603	540	601	341	331	115	49	17	0	3	313	115	33	8	20	16	872	54	628	152	46
PFOS_INTERSECTION-%-gene (82)	10	12	6	0	48	0	46	47	63	57	62	56	48	51	34	13	1	3	57	57	32	6	15	16	54	82	70	58	37
S-550_INTERSECTION-%-gene (976)	14	22	10	0	62	0	663	125	731	705	762	485	425	168	64	20	1	2	347	142	40	8	21	18	628	70	976	199	54
SOS_INTERSECTION-%-gene (246)	14	16	9	0	54	0	161	92	184	184	197	180	161	106	43	18	1	3	114	96	34	8	19	17	152	58	199	246	43
Wyeth-14643_INTERSECTION-%-gene (66)	12	10	9	0	32	0	38	44	54	44	52	47	42	40	29	15	1	3	40	47	32	8	19	17	46	37	54	43	66

Supplemental Table S8: Pathway-level BMC similarities for the intersection of independent experiments.

Row	2-(2-Butoxyethoxy)ethanol_INTERSECTION-%-WP (17)	2-methyl-2,4-pentanediol_INTERSECTION-%-WP (51)	4,2-FTS_INTERSECTION-%-WP (13)	6,2-FTOH_INTERSECTION-%-WP (0)	6,2-FTS_INTERSECTION-%-WP (90)	8,2-FTOH_INTERSECTION-%-WP (0)	AFFF-Qpool_INTERSECTION-%-WP (416)	AFFF1-Solberg_INTERSECTION-%-WP (144)	AFFF2-Chemguard_INTERSECTION-%-WP (448)	AFFF3-Tridol_INTERSECTION-%-WP (423)	AFFF4-Phos-Chek_INTERSECTION-%-WP (426)	AFFF5-Fomtec_INTERSECTION-%-WP (367)	Laurylamidopropyl betaine_INTERSECTION-%-WP (222)	OMP_INTERSECTION-%-WP (112)	PB_INTERSECTION-%-WP (28)	PEG_INTERSECTION-%-WP (2)	PFBS_INTERSECTION-%-WP (8)	PFDA_INTERSECTION-%-WP (356)	PFHpA_INTERSECTION-%-WP (166)	PFHpS_INTERSECTION-%-WP (56)	PFHxA_INTERSECTION-%-WP (16)	PFHxS_INTERSECTION-%-WP (34)	PFNA_INTERSECTION-%-WP (26)	PFOA_INTERSECTION-%-WP (442)	PFOS_INTERSECTION-%-WP (90)	S-550_INTERSECTION-%-WP (442)	SOS_INTERSECTION-%-WP (257)	Wyeth-14643_INTERSECTION-%-WP (78)
2-(2-Butoxyethoxy)ethanol_INTERSECTION-%-WP (17)	17	13	10	0	16	0	15	17	17	14	15	15	16	15	17	1	7	17	17	17	12	17	12	17	17	17	17	17
2-methyl-2,4-pentanediol_INTERSECTION-%-WP (51)	13	51	13	0	44	0	51	46	50	51	51	51	49	46	23	2	7	50	50	38	12	25	20	51	45	51	51	40
4,2-FTS_INTERSECTION-%-WP (13)	10	13	13	0	13	0	13	13	13	13	13	13	13	13	11	2	4	13	13	13	9	12	12	13	13	13	13	13
6,2-FTOH_INTERSECTION-%-WP (0)	0	0	0	0	0	0	0	0	0	0	0	0	0	0	0	0	0	0	0	0	0	0	0	0	0	0	0	0
6,2-FTS_INTERSECTION-%-WP (90)	16	44	13	0	90	0	88	75	88	87	87	86	81	64	26	2	8	87	76	51	16	31	25	88	72	89	87	59
8,2-FTOH_INTERSECTION-%-WP (0)	0	0	0	0	0	0	0	0	0	0	0	0	0	0	0	0	0	0	0	0	0	0	0	0	0	0	0	0
AFFF-Qpool_INTERSECTION-%-WP (416)	15	51	13	0	88	0	416	140	408	395	399	351	219	110	26	2	8	338	161	54	15	32	26	399	88	407	248	75
AFFF1-Solberg_INTERSECTION-%-WP (144)	17	46	13	0	75	0	140	144	142	139	139	139	123	86	28	2	8	136	104	52	15	31	23	143	71	143	138	68
AFFF2-Chemguard_INTERSECTION-%-WP (448)	17	50	13	0	88	0	408	142	448	410	411	357	219	109	27	2	8	351	163	56	16	34	26	421	90	428	252	77
AFFF3-Tridol_INTERSECTION-%-WP (423)	14	51	13	0	87	0	395	139	410	423	404	358	218	107	25	2	7	344	160	52	15	30	26	404	86	410	249	75
AFFF4-Phos-Chek_INTERSECTION-%-WP (426)	15	51	13	0	87	0	399	139	411	404	426	354	219	108	26	2	8	341	159	53	15	31	26	408	87	407	251	76
AFFF5-Fomtec_INTERSECTION-%-WP (367)	15	51	13	0	86	0	351	139	357	358	354	367	215	108	26	2	7	320	162	54	15	32	26	359	88	357	251	75
Laurylamidopropyl betaine_INTERSECTION-%-WP (222)	16	49	13	0	81	0	219	123	219	218	219	215	222	88	25	2	8	206	133	51	16	31	25	218	80	219	191	70
OMP_INTERSECTION-%-WP (112)	15	46	13	0	64	0	110	86	109	107	108	108	88	112	25	2	8	108	94	46	13	27	21	109	60	109	107	57
PB_INTERSECTION-%-WP (28)	17	23	11	0	26	0	26	28	27	25	26	26	25	25	28	1	7	27	28	27	13	21	15	28	27	28	28	27
PEG_INTERSECTION-%-WP (2)	1	2	2	0	2	0	2	2	2	2	2	2	2	2	1	2	1	2	2	2	1	2	2	2	2	2	2	2
PFBS_INTERSECTION-%-WP (8)	7	7	4	0	8	0	8	8	8	7	8	7	8	8	7	1	8	8	8	8	6	8	6	8	8	8	8	8
PFDA_INTERSECTION-%-WP (356)	17	50	13	0	87	0	338	136	351	344	341	320	206	108	27	2	8	356	161	55	16	33	25	348	89	350	235	74
PFHpA_INTERSECTION-%-WP (166)	17	50	13	0	76	0	161	104	163	160	159	162	133	94	28	2	8	161	166	53	16	34	26	162	83	163	152	74
PFHpS_INTERSECTION-%-WP (56)	17	38	13	0	51	0	54	52	56	52	53	54	51	46	27	2	8	55	53	56	16	34	23	56	51	56	55	47
PFHxA_INTERSECTION-%-WP (16)	12	12	9	0	16	0	15	15	16	15	15	15	16	13	13	1	6	16	16	16	16	16	14	16	16	16	16	16
PFHxS_INTERSECTION-%-WP (34)	17	25	12	0	31	0	32	31	34	30	31	32	31	27	21	2	8	33	34	34	16	34	21	34	32	34	33	32
PFNA_INTERSECTION-%-WP (26)	12	20	12	0	25	0	26	23	26	26	26	26	25	21	15	2	6	25	26	23	14	21	26	26	24	26	26	25
PFOA_INTERSECTION-%-WP (442)	17	51	13	0	88	0	399	143	421	404	408	359	218	109	28	2	8	348	162	56	16	34	26	442	89	415	255	77
PFOS_INTERSECTION-%-WP (90)	17	45	13	0	72	0	88	71	90	86	87	88	80	60	27	2	8	89	83	51	16	32	24	89	90	90	88	60
S-550_INTERSECTION-%-WP (442)	17	51	13	0	89	0	407	143	428	410	407	357	219	109	28	2	8	350	163	56	16	34	26	415	90	442	250	77
SOS_INTERSECTION-%-WP (257)	17	51	13	0	87	0	248	138	252	249	251	251	191	107	28	2	8	235	152	55	16	33	26	255	88	250	257	76
Wyeth-14643_INTERSECTION-%-WP (78)	17	40	13	0	59	0	75	68	77	75	76	75	70	57	27	2	8	74	74	47	16	32	25	77	60	77	76	78

Supplemental Table S9: Jaccard Index comparisons for biological response similarities for the intersecting gene- and pathway-level transcriptomic BMCs across independent experiments from AFFF product exposures to human hepatocytes.

	AFFF1 (145)	AFFF2 (878)	AFFF3 (804)	AFFF4 (897)	AFFF5 (539)
**AFFF1** (145)		118<br>0.1304	109<br>0.1298	118<br>0.1277	116<br>0.2042
**AFFF2** (878)	118<br>0.1304		656<br>0.6394	693<br>0.6405	446<br>0.4593
**AFFF3** (804)	109<br>0.1298	656<br>0.6394		720<br>0.7339	479<br>0.5544
**AFFF4** (897)	118<br>0.1277	693<br>0.6405	720<br>0.7339		482<br>0.5052
**AFFF5** (539)	116<br>0.2042	446<br>0.4593	479<br>0.5544	482<br>0.5052	

0 Jaccard index 1

	AFFF1 (144)	AFFF2 (448)	AFFF3 (423)	AFFF4 (426)	AFFF5 (367)
**AFFF1** (144)		142<br>0.3156	139<br>0.3248	139<br>0.3225	139<br>0.3737
**AFFF2** (448)	142<br>0.3156		410<br>0.8894	411<br>0.8877	357<br>0.7795
**AFFF3** (423)	139<br>0.3248	410<br>0.8894		404<br>0.9079	358<br>0.8287
**AFFF4** (426)	139<br>0.3225	411<br>0.8877	404<br>0.9079		354<br>0.8064
**AFFF5** (367)	139<br>0.3737	357<br>0.7795	358<br>0.8287	354<br>0.8064	

0 Jaccard index 1

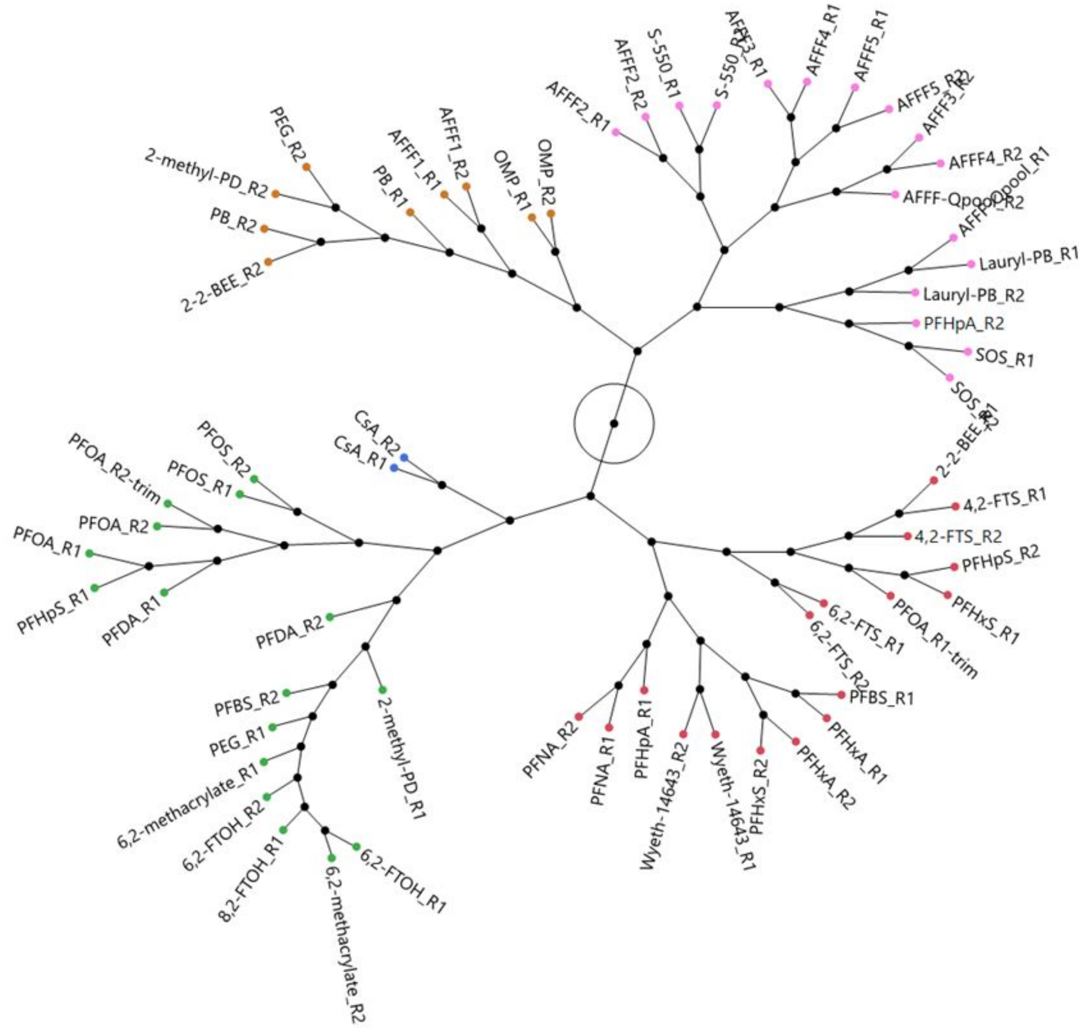

Supplemental Figure S10: Potency-only constellation plot of hierarchical clustering of rank scores for the top 100 most potent gene-level BMCs from each of the 60 experimental runs (30 test substances x 2 runs).

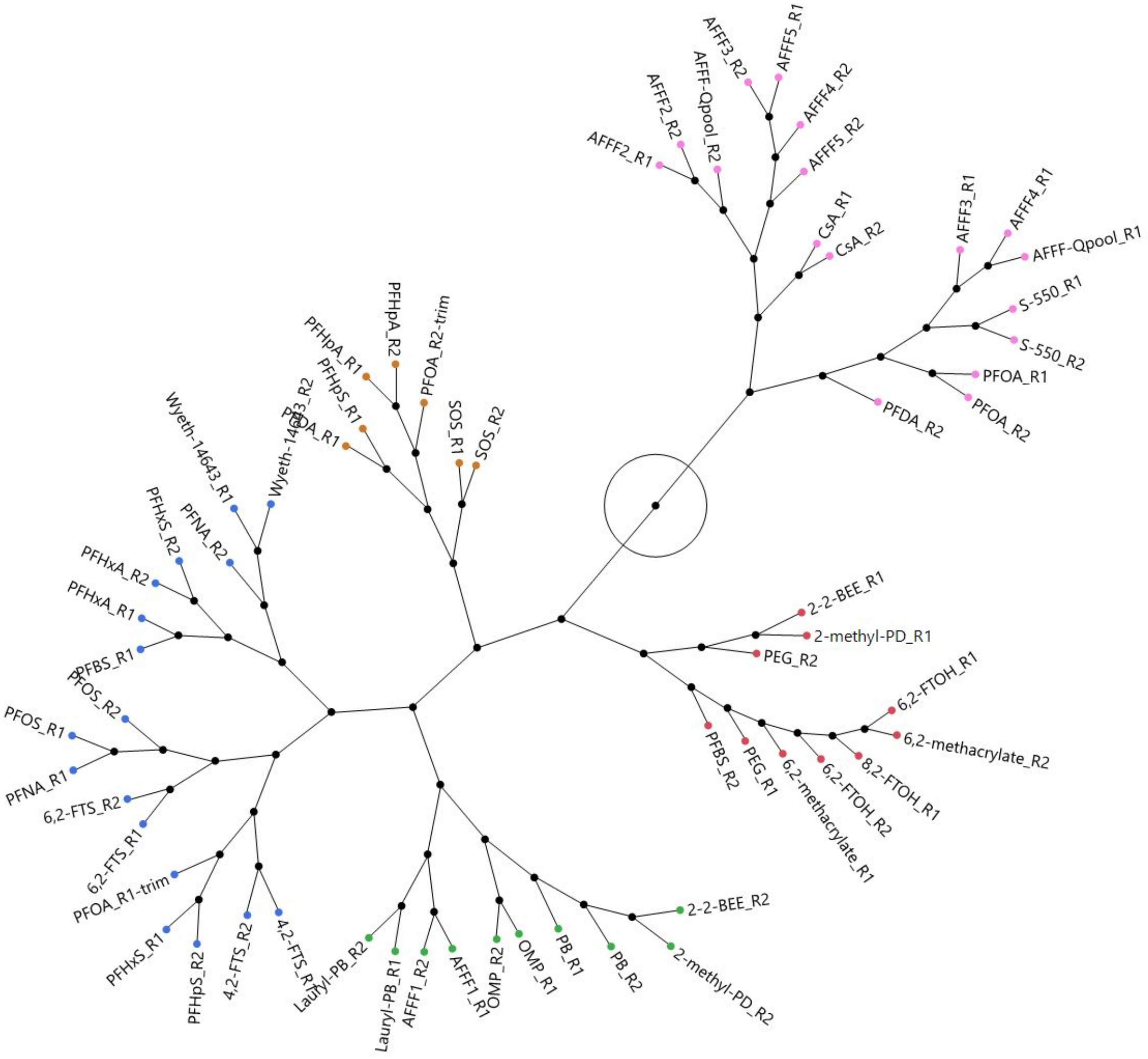

Supplemental Figure S11: Fold-change-only constellation plot of hierarchical clustering of rank scores for the top 100 most efficacious (fold-change) gene-level BMCs from each of the 60 experimental runs (30 test substances x 2 runs).

Supplemental Table S12: Comparison of in vivo LOEL values to in vitro potencies for CAR, PPARα, and liver injury.

Test Chemical Name	In Vivo Liver Weight LOEL (μM, plasma)	In Vitro CAR BMC (μM, media)	In Vitro PPARα BMC (μM, media)	In Vitro Liver Injury BMC105 (μM, media)
PFOS	47.4	63	144	190
PFHxS	230	136	206	N/A
PFBS	360* (17.9)	352†	222†	N/A
PFDA	16.5	219	204	52
PFNA	122	28	49	125
PFOA	122	139	97	81
PFHxA	100* (10.6)	167	99	N/A
Wyeth-14,643	18	26	25	85
PB	86-172	130	320	N/A
6,2-FTS	80-170‡	80	170	372

†Data not filtered to enable more sensitive pathway detection.
*Extrapolated peak concentrations to account for 24-hour post-dose sampling of plasma with short half-life PFAS.
N/A: Not applicable at the exposure ranges examined.
‡Predicted in vivo liver weight LOEL range from observed in vitro potencies (internal dose).

Supplemental Table S13: Summary of CAR and PPARα BMC values including statistically-derived upper and lower bounds.

Analysis	GO/Pathway/Gene Set/Gene ID	GO/Pathway/Gene Set/Gene Name	BMD Median	BMDL Median	BMDU Median	Max Fold Change	Genes That Passed All Filters	Overall Direction	Percentage
6,2-FTS_Run1-uM-WP	M39547	WP_PPAR_ALPHA_PATHWAY	180.3	130.2	288.1	6.3	6	UP	42.86
6,2-FTS_Run2-uM-WP	M39547	WP_PPAR_ALPHA_PATHWAY	159.8	120.4	240.3	4.7	5	UP	35.71
PFBS_Run1-uM-WP	M39547	WP_PPAR_ALPHA_PATHWAY	126.3	96.5	179.1	2.1	1	DOWN	7.14
PFBS_Run2-uM-WP	M39547	WP_PPAR_ALPHA_PATHWAY	318.7	205.1	713.2	2.2	1	UP	7.14
PFDA_Run1-uM-WP	M39547	WP_PPAR_ALPHA_PATHWAY	55.3	38.2	94.4	8.8	5	UP	35.71
PFDA_Run2-uM-WP	M39547	WP_PPAR_ALPHA_PATHWAY	353.1	171.7	445.1	69.6	7	DOWN	50
PFHxA_Run1-uM-WP	M39547	WP_PPAR_ALPHA_PATHWAY	109.9	82.2	164.9	5.1	2	UP	14.29
PFHxA_Run2-uM-WP	M39547	WP_PPAR_ALPHA_PATHWAY	88.0	51.5	127.3	5.1	2	UP	14.29
PFHxS_Run1-uM-WP	M39547	WP_PPAR_ALPHA_PATHWAY	201.3	147.0	318.4	4.9	3	UP	21.43
PFHxS_Run2-uM-WP	M39547	WP_PPAR_ALPHA_PATHWAY	211.8	157.6	328.9	4.7	4	UP	28.57
PFNA_Run1-uM-WP	M39547	WP_PPAR_ALPHA_PATHWAY	29.8	21.8	38.4	8.9	4	UP	28.57
PFNA_Run2-uM-WP	M39547	WP_PPAR_ALPHA_PATHWAY	68.6	38.1	101.5	4.3	2	UP	14.29
PFOA_Run1-uM-WP	M39547	WP_PPAR_ALPHA_PATHWAY	117.2	77.8	244.5	8.3	4	UP	28.57
PFOA_Run2-uM-WP	M39547	WP_PPAR_ALPHA_PATHWAY	77.0	52.2	146.9	26.3	8	UP	57.14
PFOS_Run1-uM-WP	M39547	WP_PPAR_ALPHA_PATHWAY	59.4	47.9	77.8	5.7	5	UP	35.71
PFOS_Run2-uM-WP	M39547	WP_PPAR_ALPHA_PATHWAY	229.2	71.6	240.1	3.2	3	UP	21.43
6,2-FTS_Run1-uM-WP	M39476	WP_CONSTITUTIVE_ANDROSTANE_RECEPTOR_PATHWAY	89.3	73.6	113.1	28.2	11	UP	47.83
6,2-FTS_Run2-uM-WP	M39476	WP_CONSTITUTIVE_ANDROSTANE_RECEPTOR_PATHWAY	70.9	50.2	116.0	22.8	10	UP	43.48
PFBS_Run1-uM-WP	M39476	WP_CONSTITUTIVE_ANDROSTANE_RECEPTOR_PATHWAY	386.3	251.1	1015.1	2.4	1	UP	4.35
PFBS_Run2-uM-WP	M39476	WP_CONSTITUTIVE_ANDROSTANE_RECEPTOR_PATHWAY	317.9	197.0	1071.2	3.2	2	UP	8.7
PFDA_Run1-uM-WP	M39476	WP_CONSTITUTIVE_ANDROSTANE_RECEPTOR_PATHWAY	53.8	34.3	87.0	39.4	14	UP	60.87
PFDA_Run2-uM-WP	M39476	WP_CONSTITUTIVE_ANDROSTANE_RECEPTOR_PATHWAY	383.4	202.0	428.9	22.4	18	DOWN	78.26
PFHxA_Run1-uM-WP	M39476	WP_CONSTITUTIVE_ANDROSTANE_RECEPTOR_PATHWAY	226.5	162.1	386.2	5.1	4	UP	17.39
PFHxA_Run2-uM-WP	M39476	WP_CONSTITUTIVE_ANDROSTANE_RECEPTOR_PATHWAY	107.5	67.1	182.4	6.2	6	UP	26.09
PFHxS_Run1-uM-WP	M39476	WP_CONSTITUTIVE_ANDROSTANE_RECEPTOR_PATHWAY	104.4	84.8	150.7	66.3	12	UP	52.17
PFHxS_Run2-uM-WP	M39476	WP_CONSTITUTIVE_ANDROSTANE_RECEPTOR_PATHWAY	167.1	125.6	243.0	35.6	8	UP	34.78
PFNA_Run1-uM-WP	M39476	WP_CONSTITUTIVE_ANDROSTANE_RECEPTOR_PATHWAY	30.7	24.1	41.5	59.1	11	UP	47.83
PFNA_Run2-uM-WP	M39476	WP_CONSTITUTIVE_ANDROSTANE_RECEPTOR_PATHWAY	26.3	21.5	33.8	10.4	4	UP	17.39
PFOA_Run1-uM-WP	M39476	WP_CONSTITUTIVE_ANDROSTANE_RECEPTOR_PATHWAY	229.3	151.1	360.3	17.3	11	UP	47.83
PFOA_Run2-uM-WP	M39476	WP_CONSTITUTIVE_ANDROSTANE_RECEPTOR_PATHWAY	49.3	34.3	68.2	37.3	12	CONFLICT	52.17
PFOS_Run1-uM-WP	M39476	WP_CONSTITUTIVE_ANDROSTANE_RECEPTOR_PATHWAY	51.3	37.2	79.8	306.7	14	UP	60.87
PFOS_Run2-uM-WP	M39476	WP_CONSTITUTIVE_ANDROSTANE_RECEPTOR_PATHWAY	74.4	50.3	101.0	116.5	9	UP	39.13

Supplemental References

1. Ramaiahgari, S.C., et al., *The power of resolution: contextualized understanding of biological responses to liver injury chemicals using high-throughput transcriptomics and benchmark concentration modeling.* Toxicological Sciences, 2019. **169**(2): p. 553-566.
