# Supplementary material for: Unraveling Human Hepatocellular Responses to PFAS and Aqueous Film-Forming Foams (AFFFs) for Molecular Hazard Prioritization and In Vivo Translation
Source: Environ Sci Technol. 2025 Feb 2;59(5):2423–35. doi: 10.1021/acs.est.4c10595 (PMC11823446; doi:10.1021/acs.est.4c10595)
Supplement: Supplementary file 8 — es4c10595_si_008.zip [file es4c10595_si_008.zip › Analyzed Data Files/BMDExpress Analysis/BMC modeling input files/BMD Graphs/BMD Graphs uM.pptx]

## Slide 1
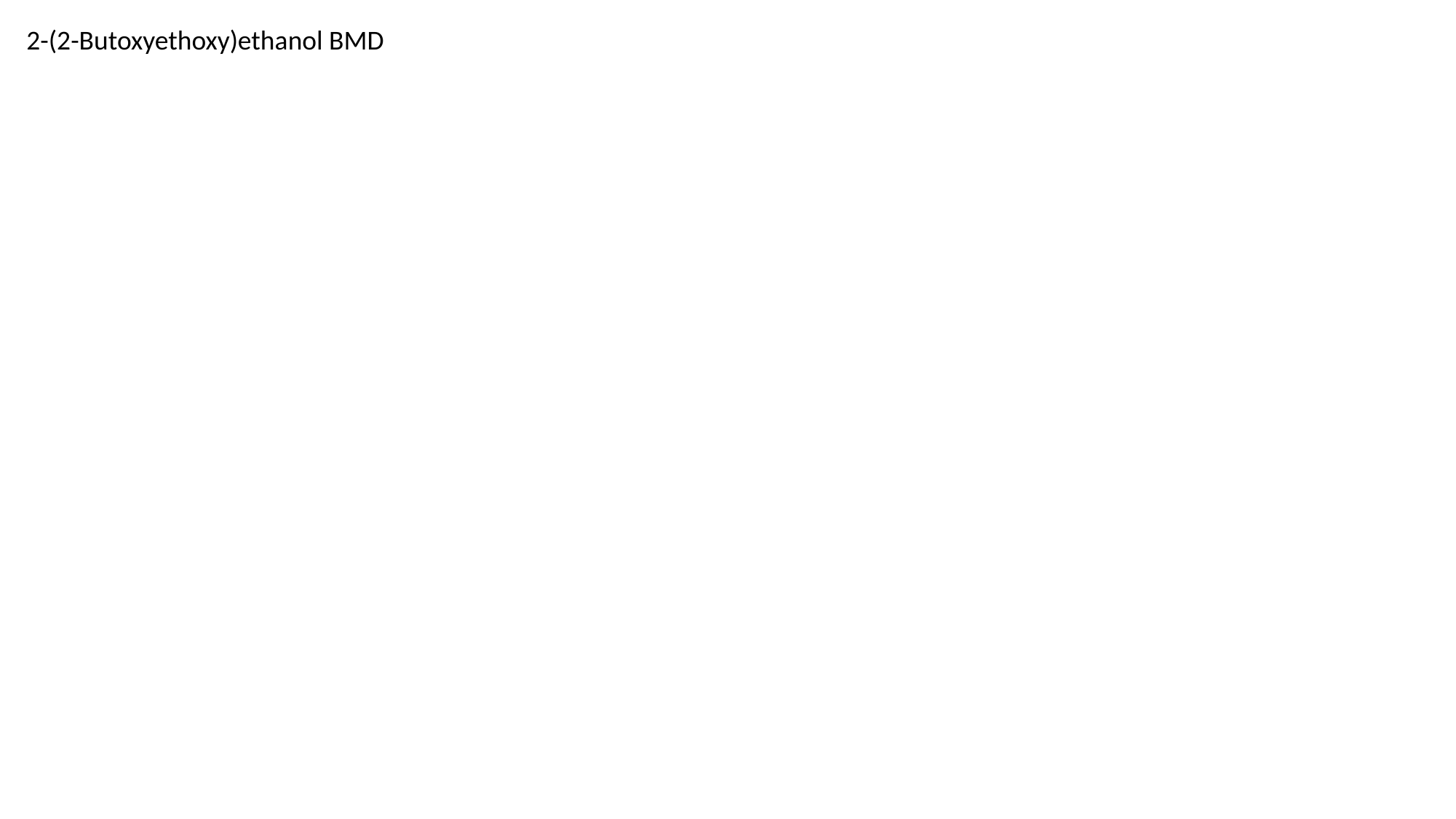

2-(2-Butoxyethoxy)ethanol BMD

## Slide 2
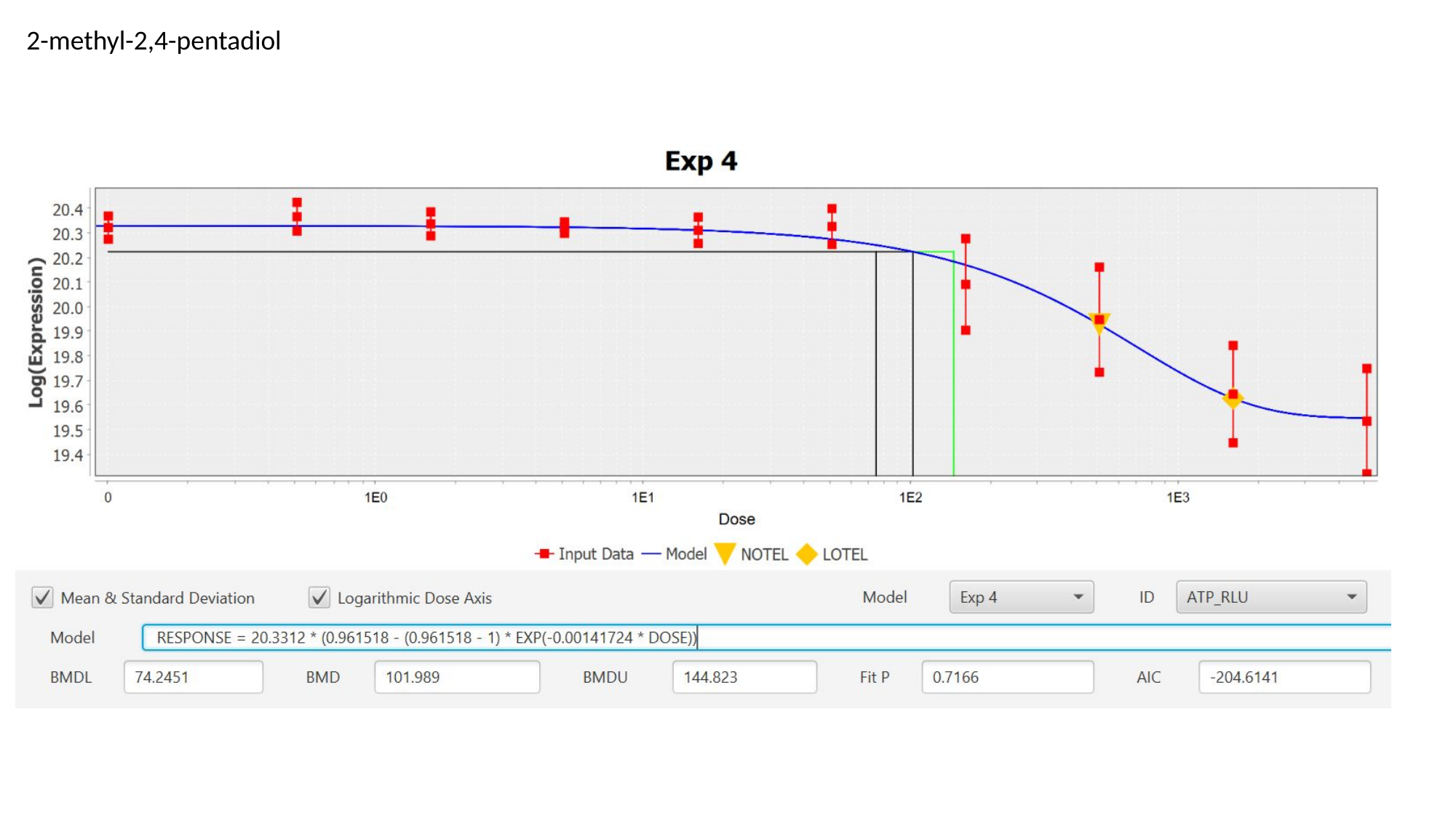

2-methyl-2,4-pentadiol

## Slide 3
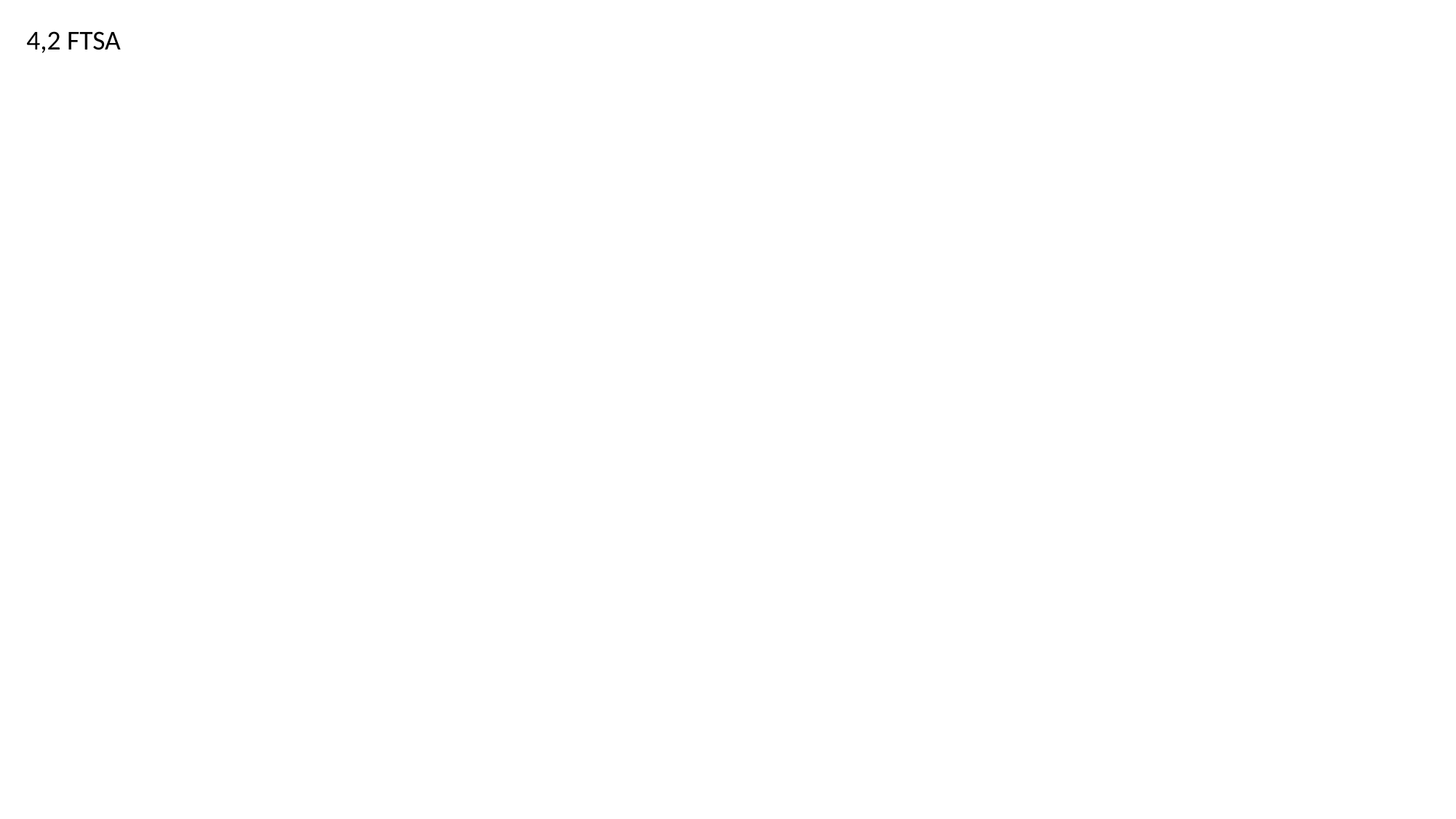

4,2 FTSA

## Slide 4
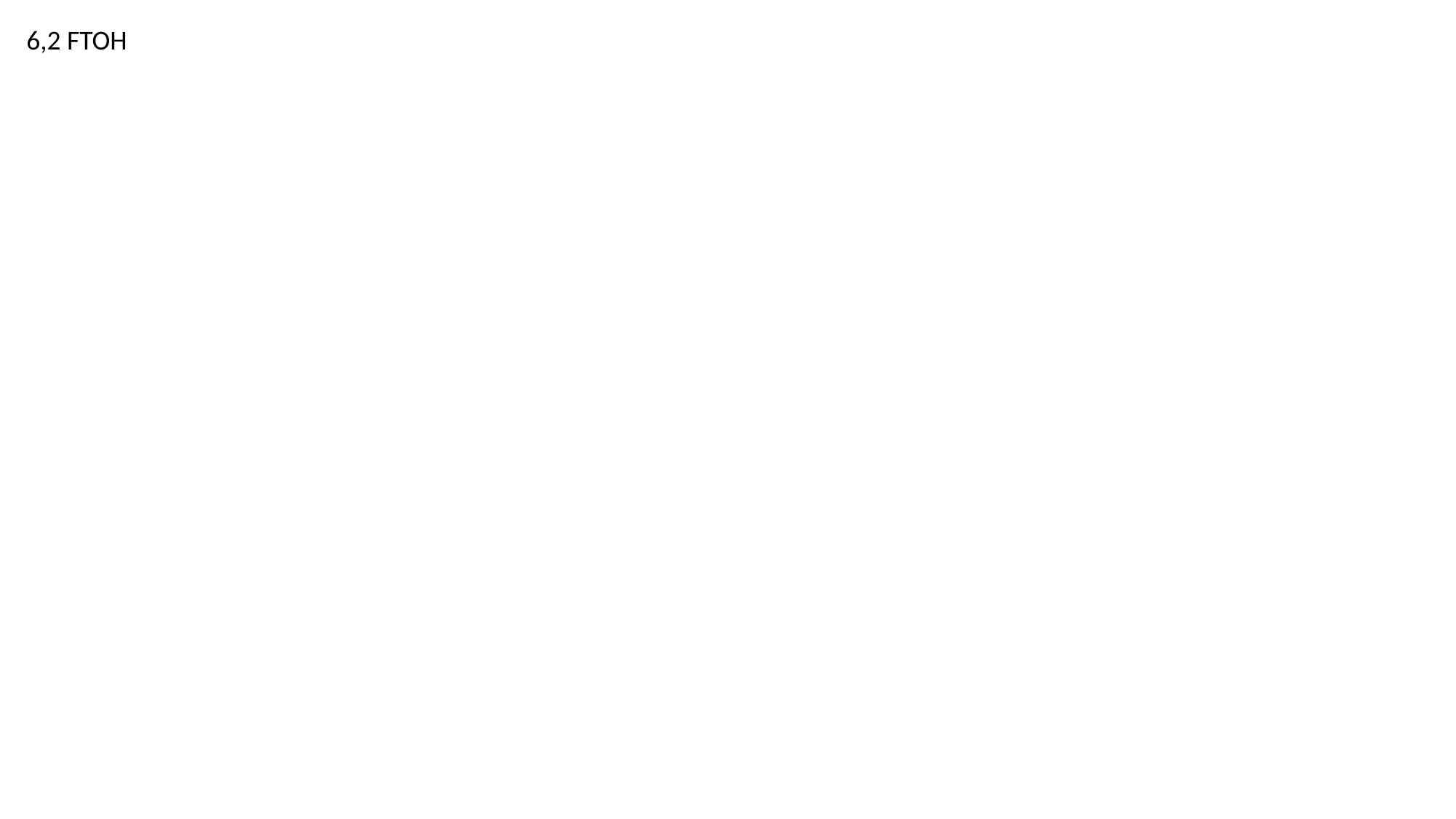

6,2 FTOH

## Slide 5
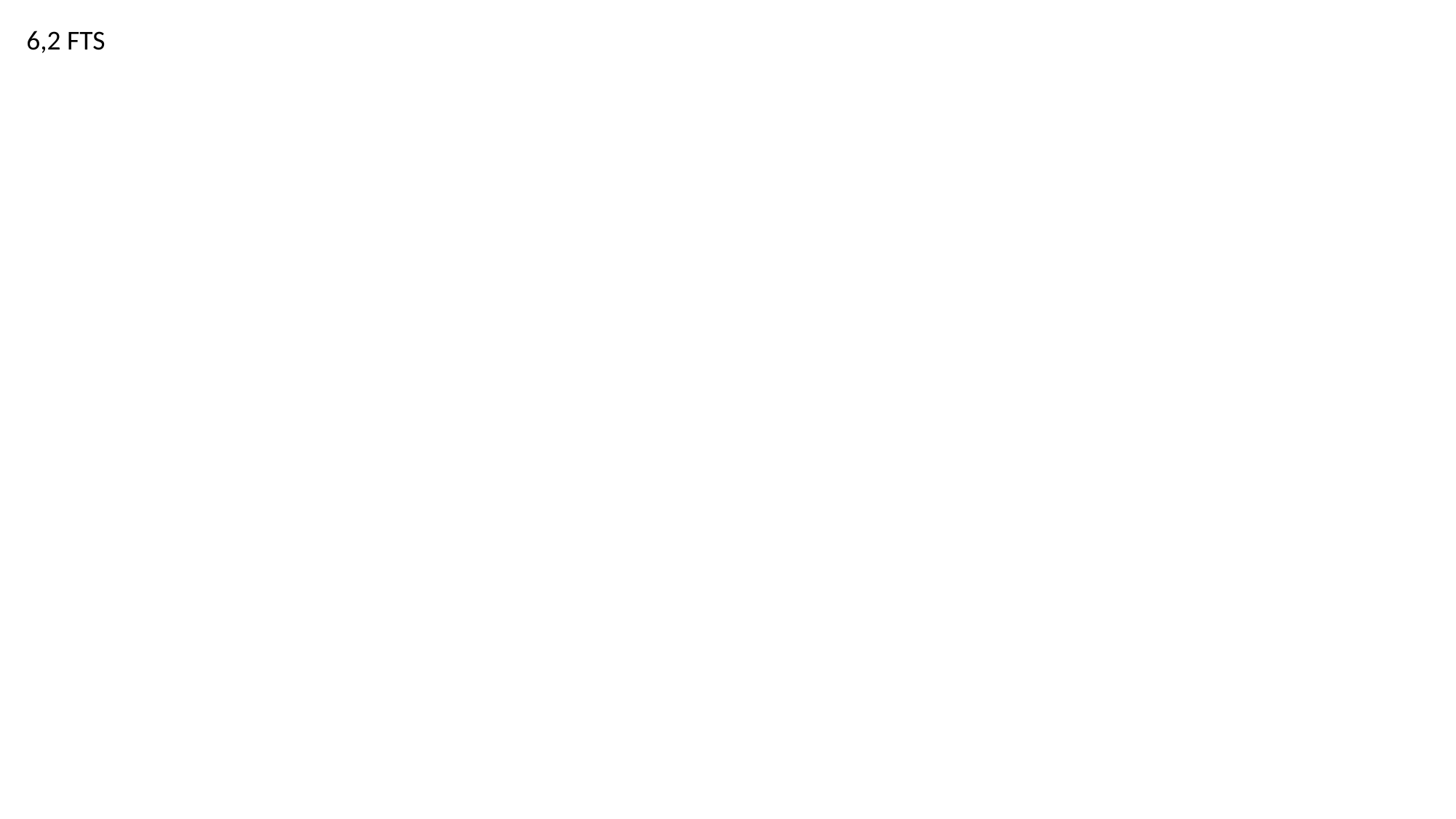

6,2 FTS

## Slide 6
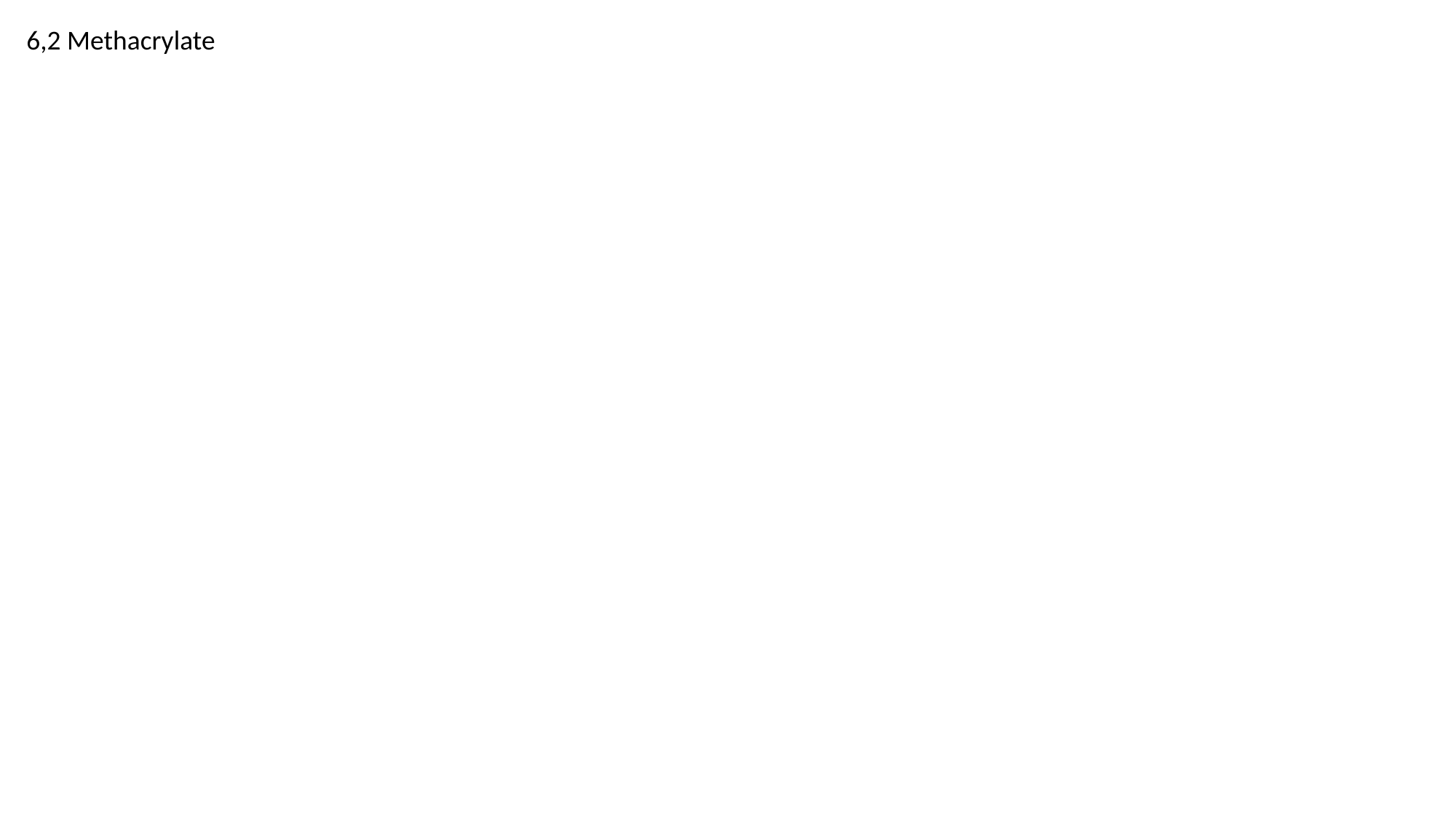

6,2 Methacrylate

## Slide 7
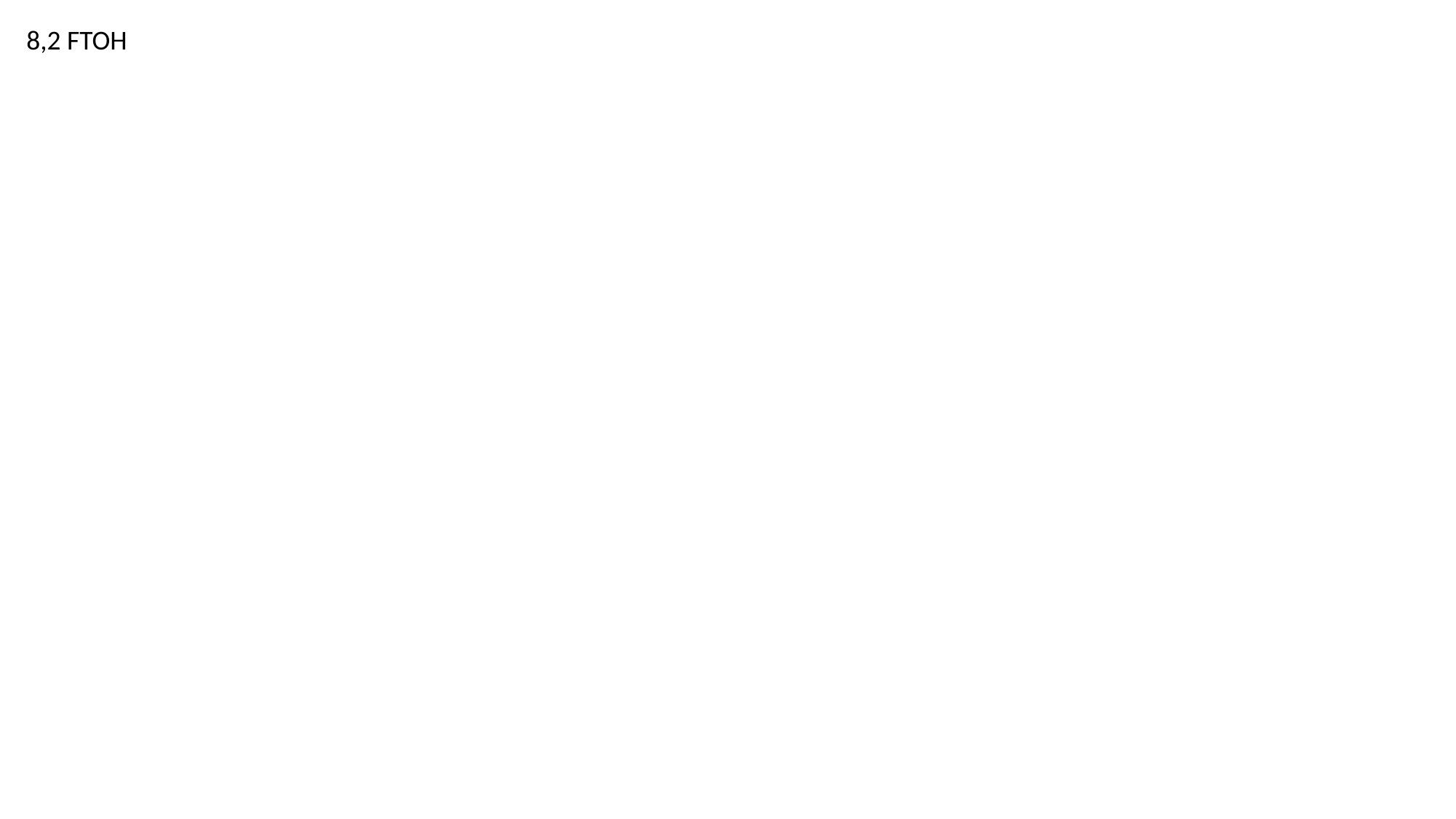

8,2 FTOH

## Slide 8
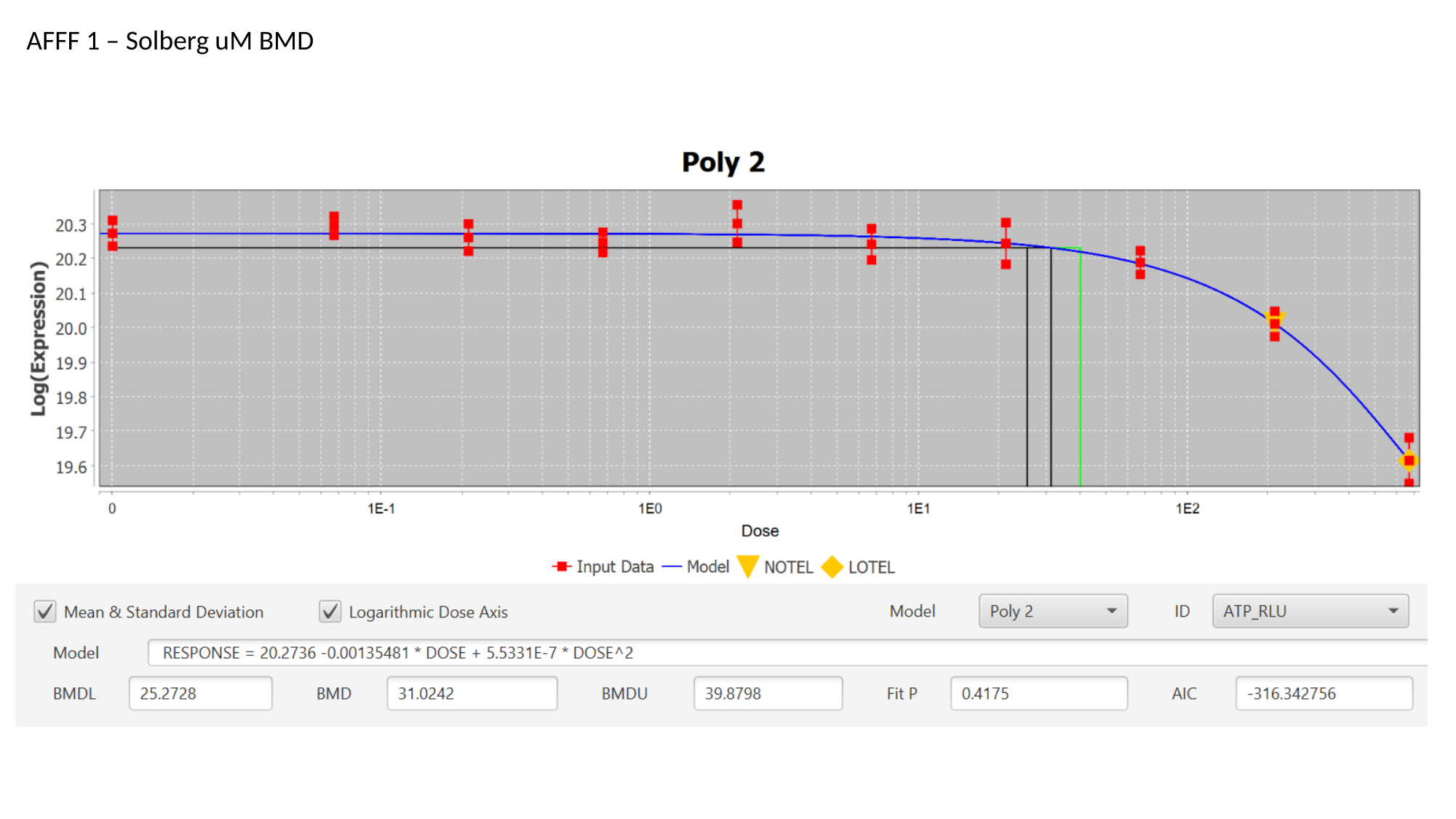

AFFF 1 – Solberg uM BMD

## Slide 9
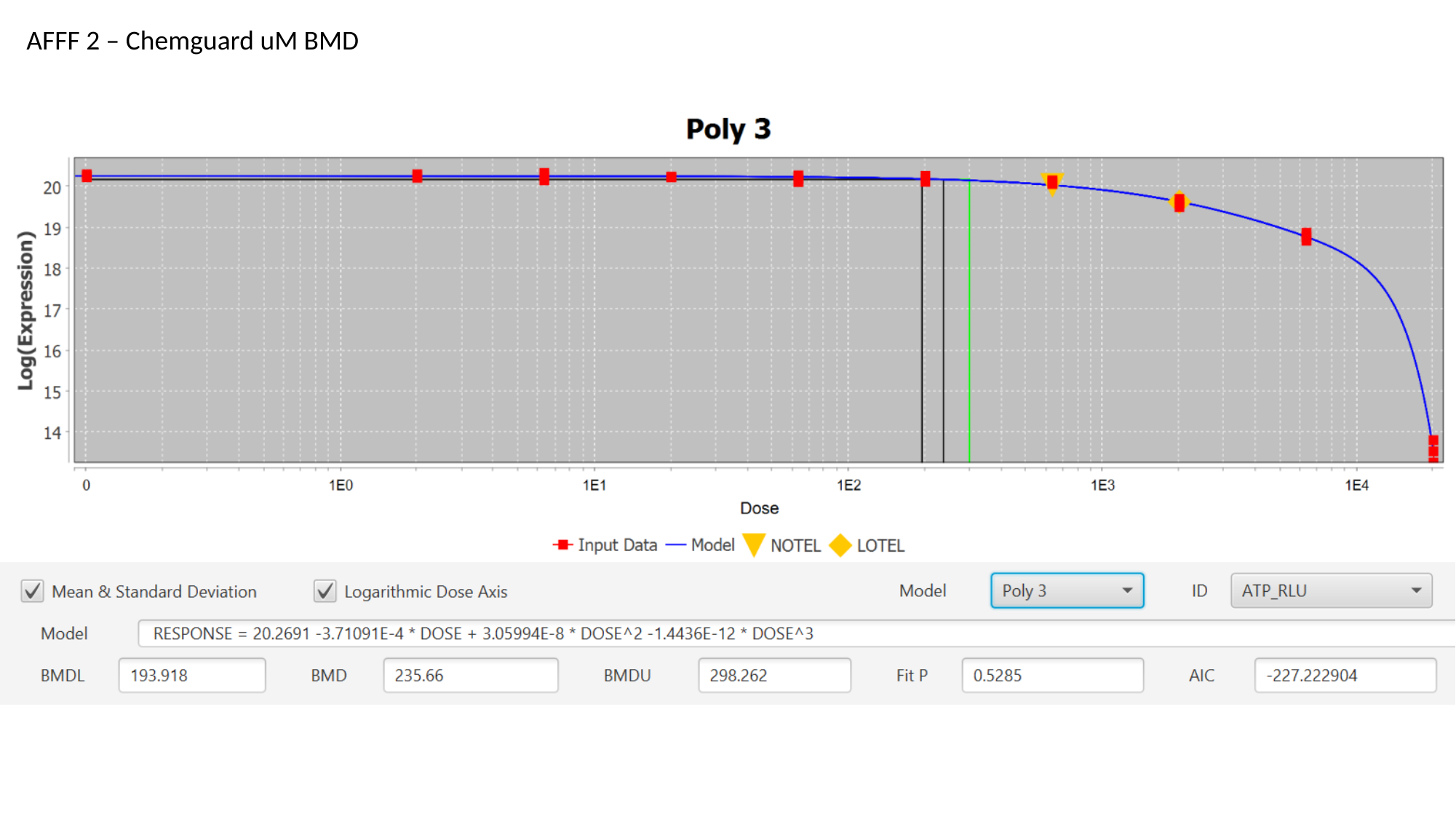

AFFF 2 – Chemguard uM BMD

## Slide 10
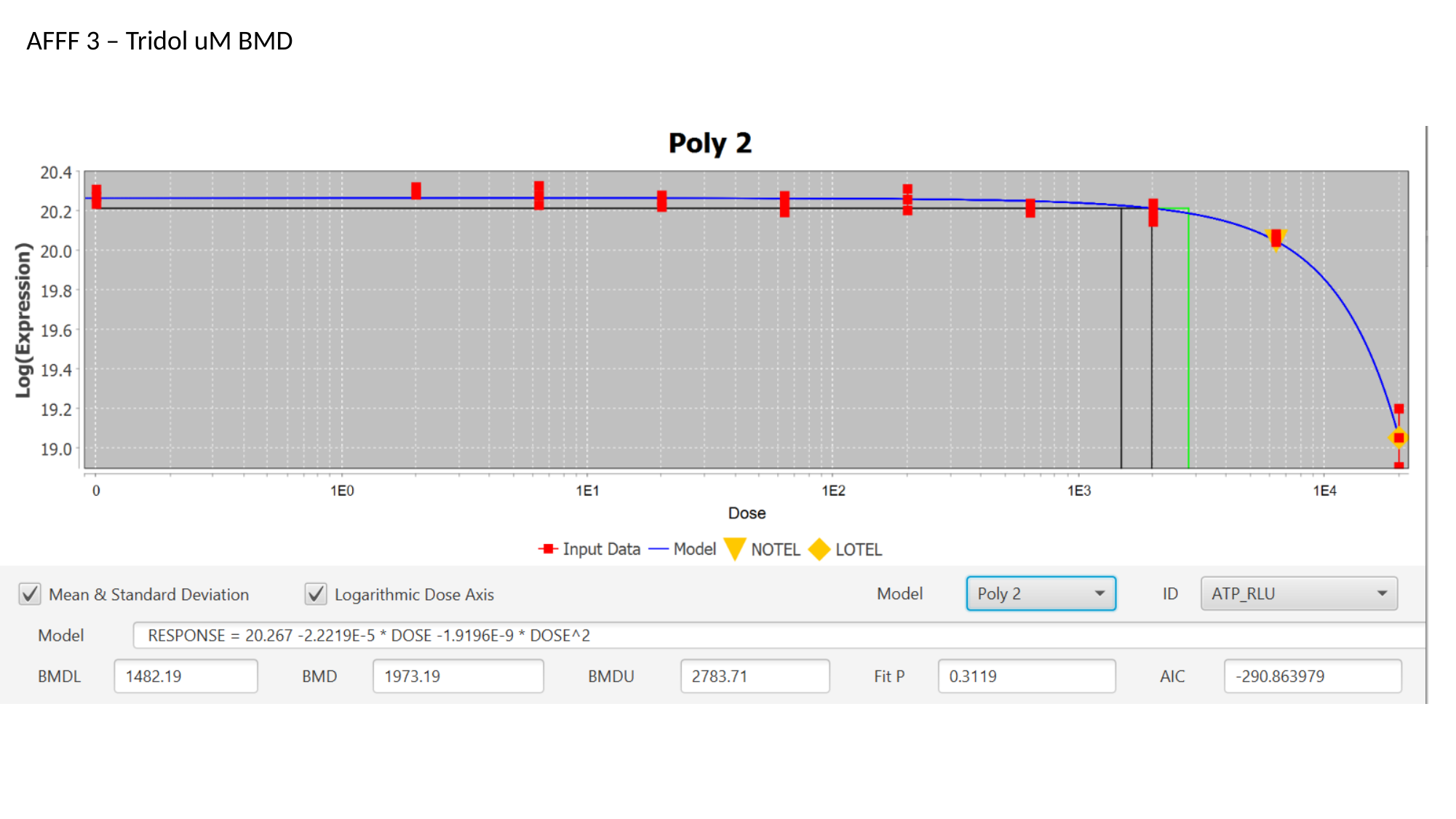

AFFF 3 – Tridol uM BMD

## Slide 11
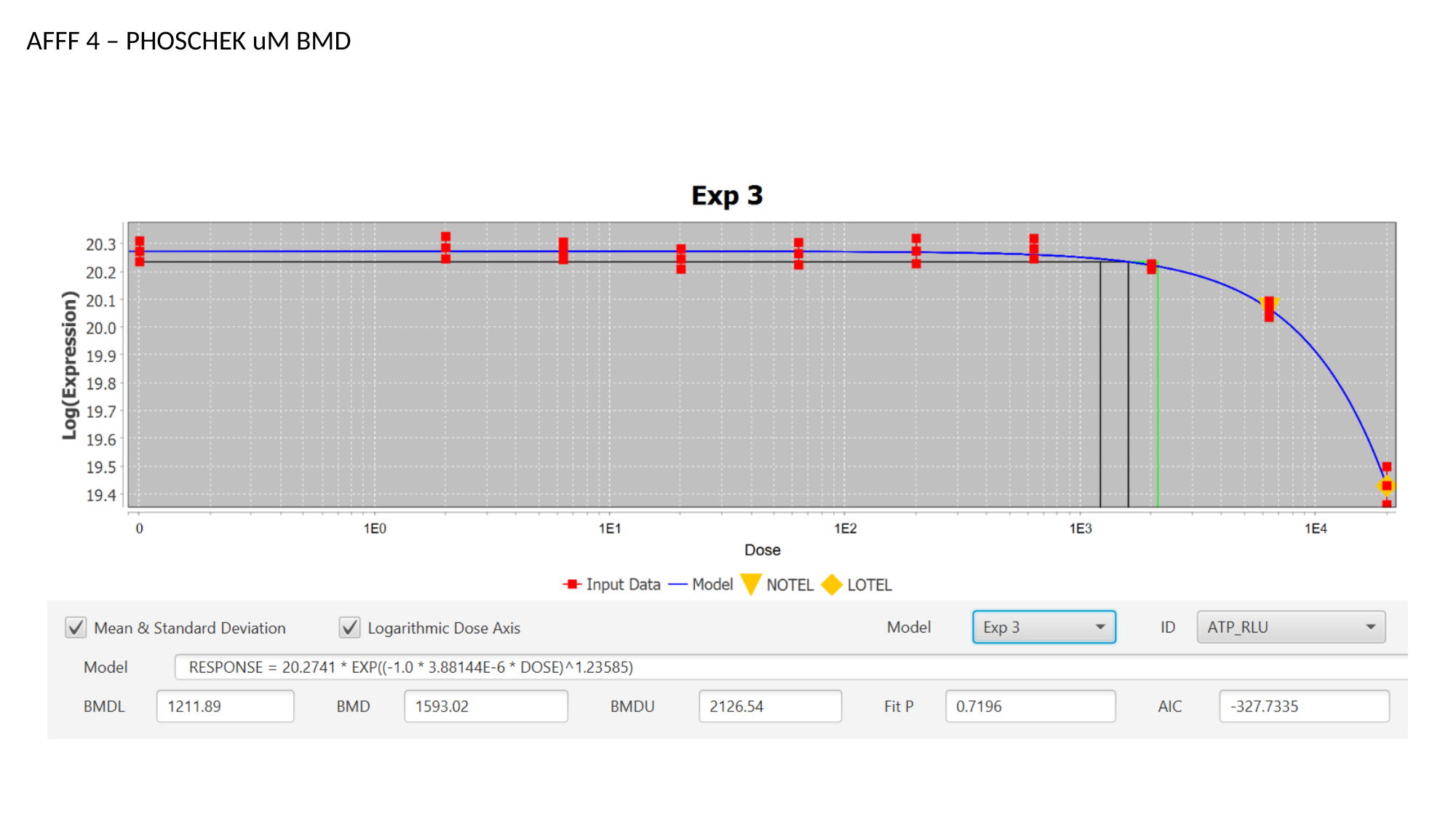

AFFF 4 – PHOSCHEK uM BMD

## Slide 12
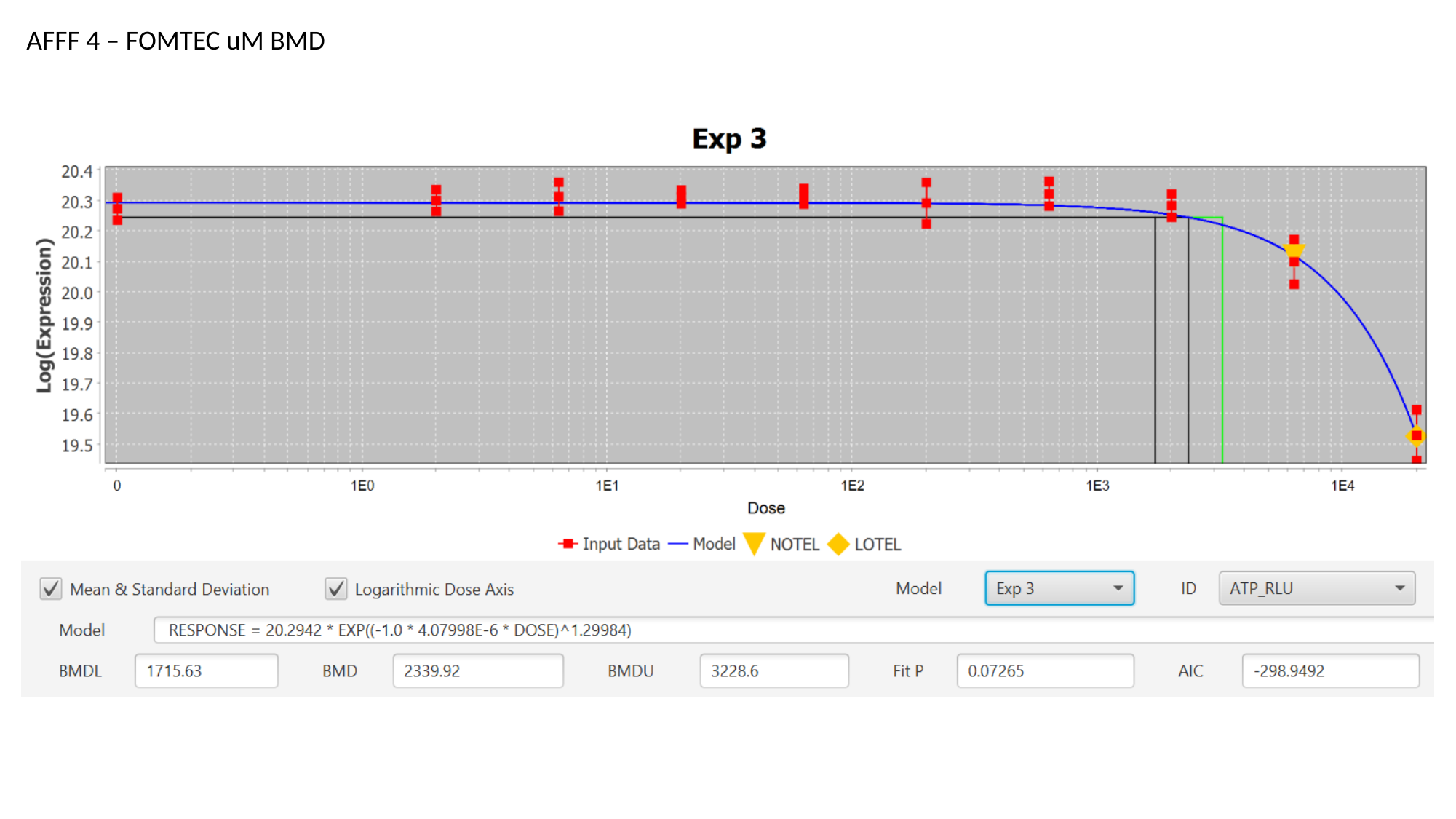

AFFF 4 – FOMTEC uM BMD

## Slide 13
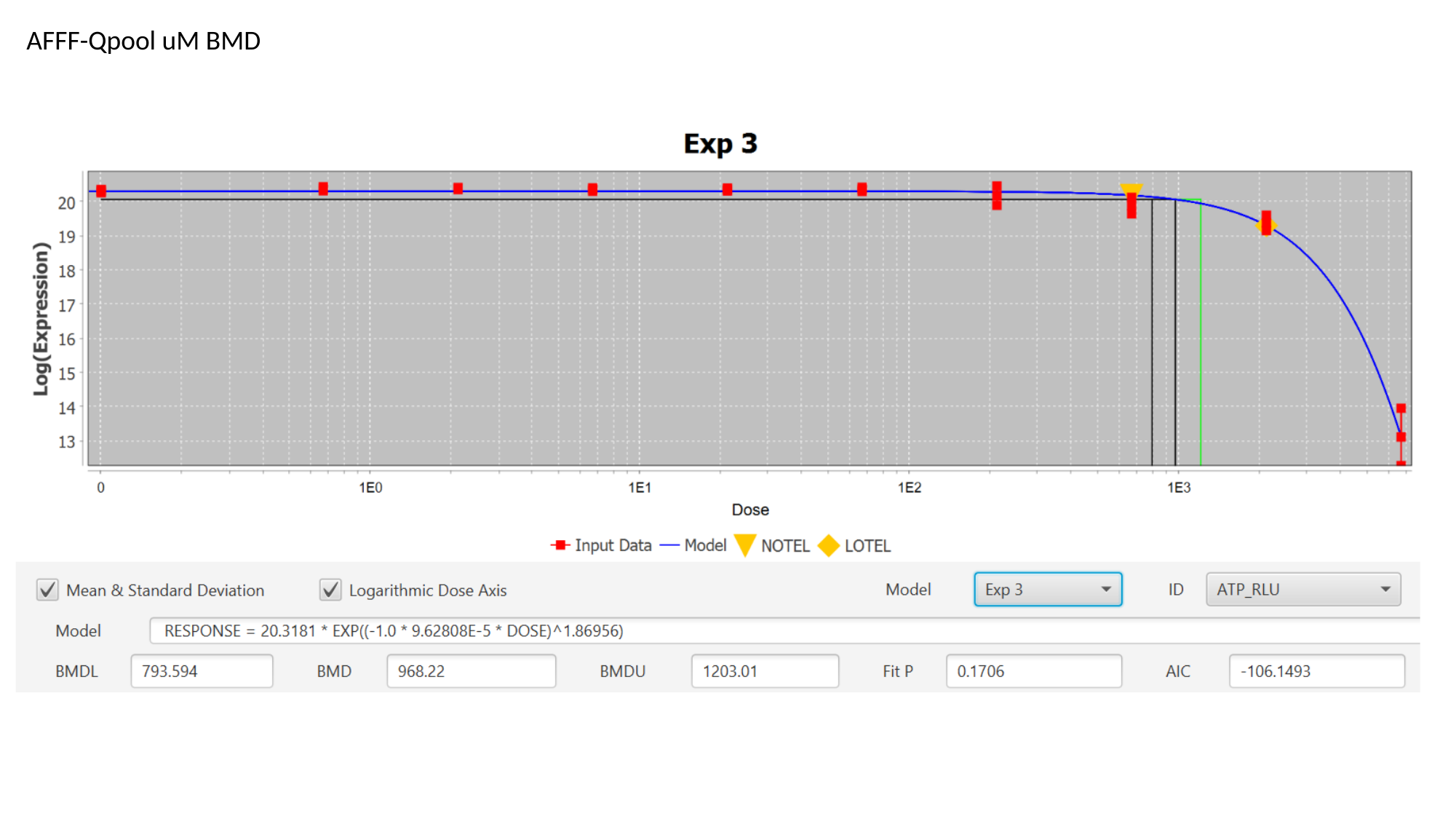

AFFF-Qpool uM BMD

## Slide 14
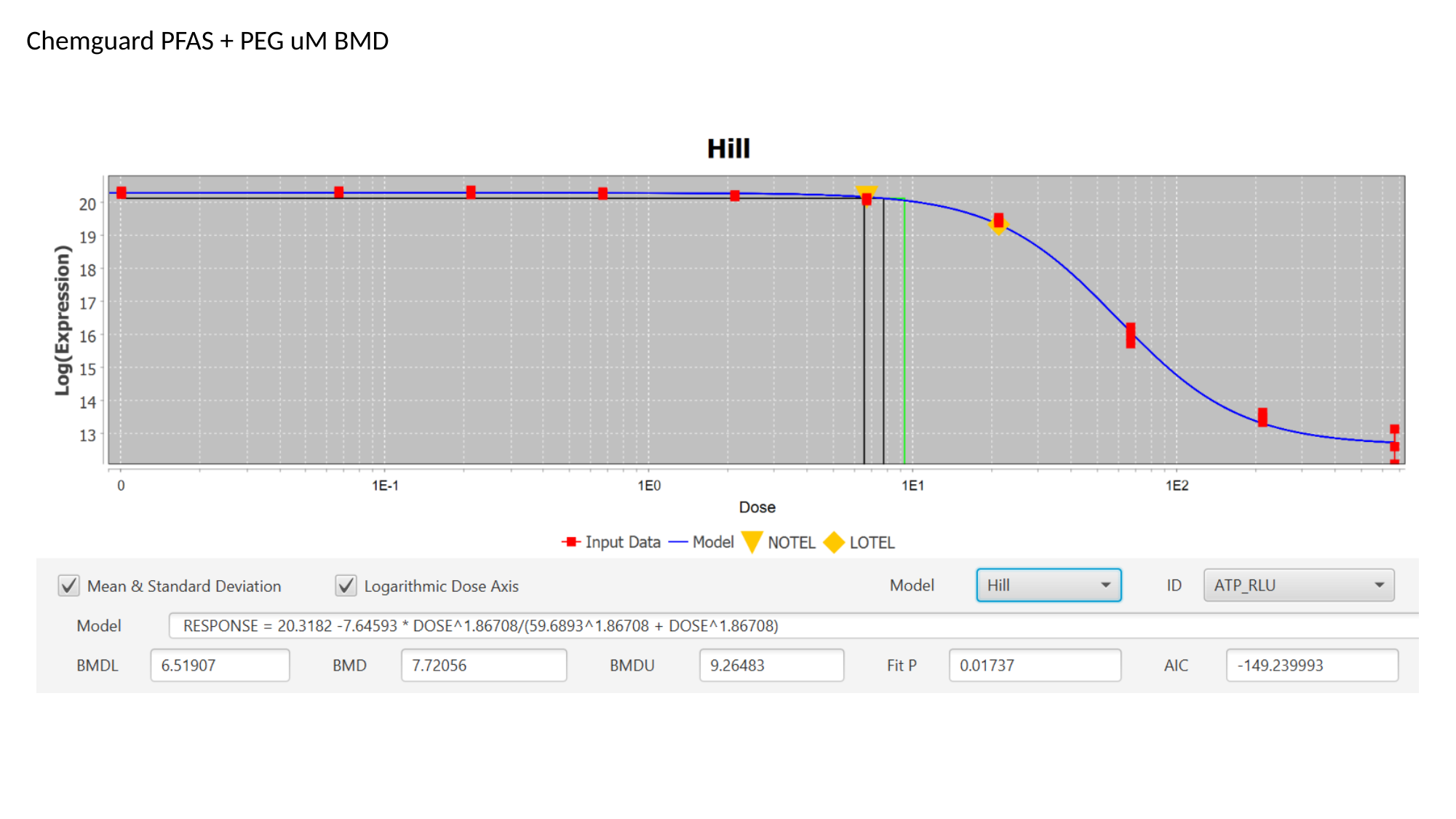

Chemguard PFAS + PEG uM BMD

## Slide 15
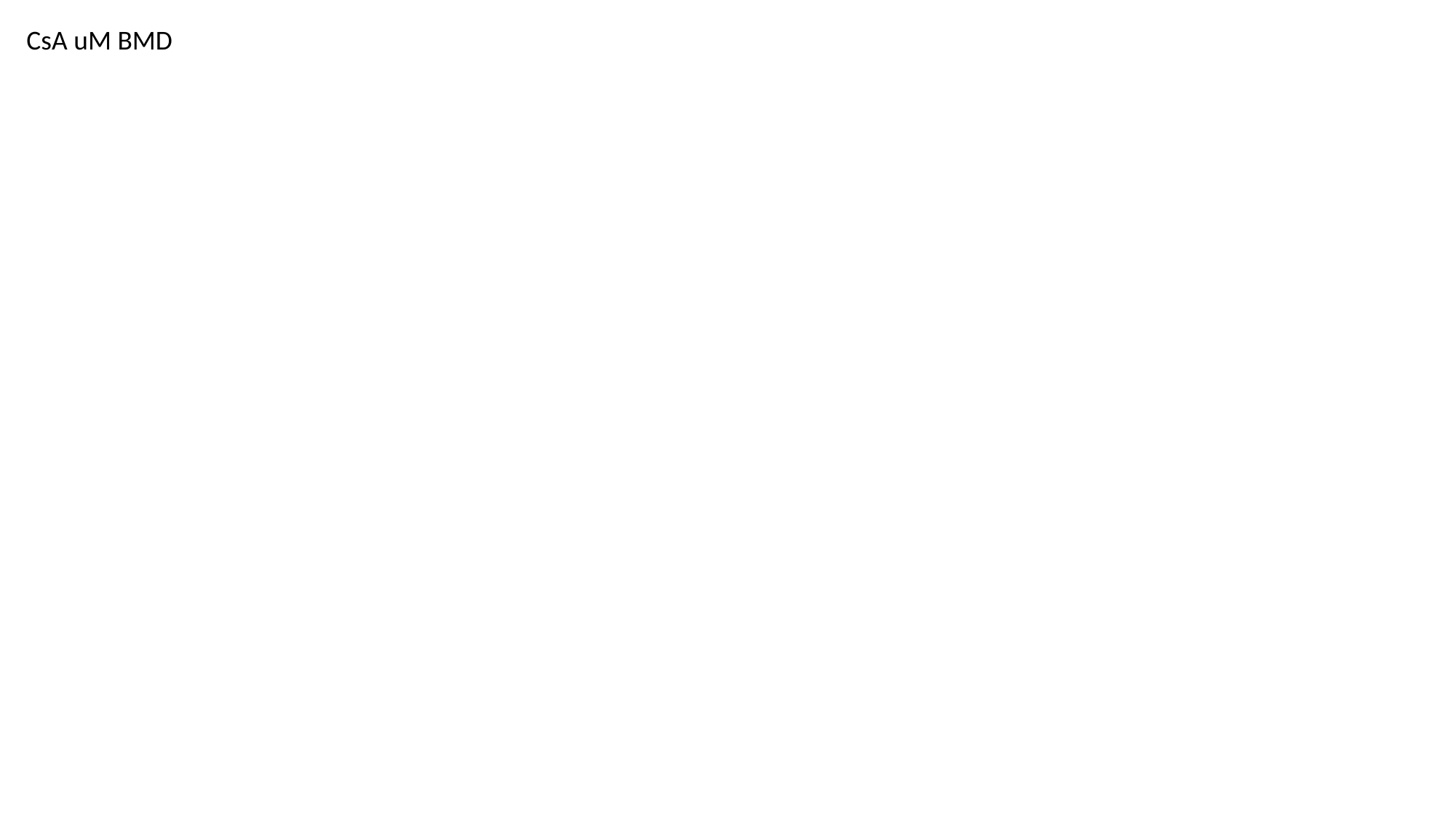

CsA uM BMD

## Slide 16
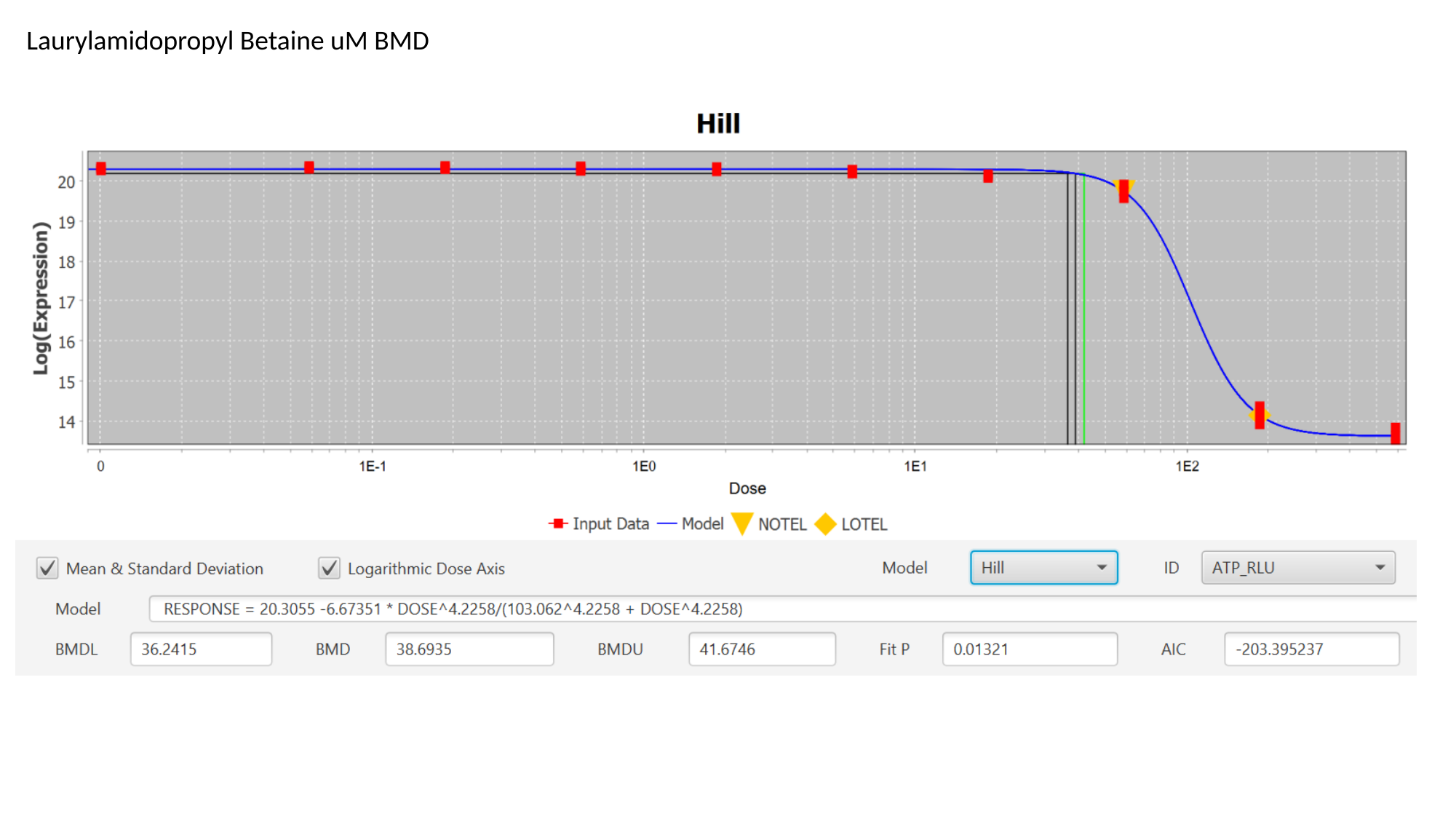

Laurylamidopropyl Betaine uM BMD

## Slide 17
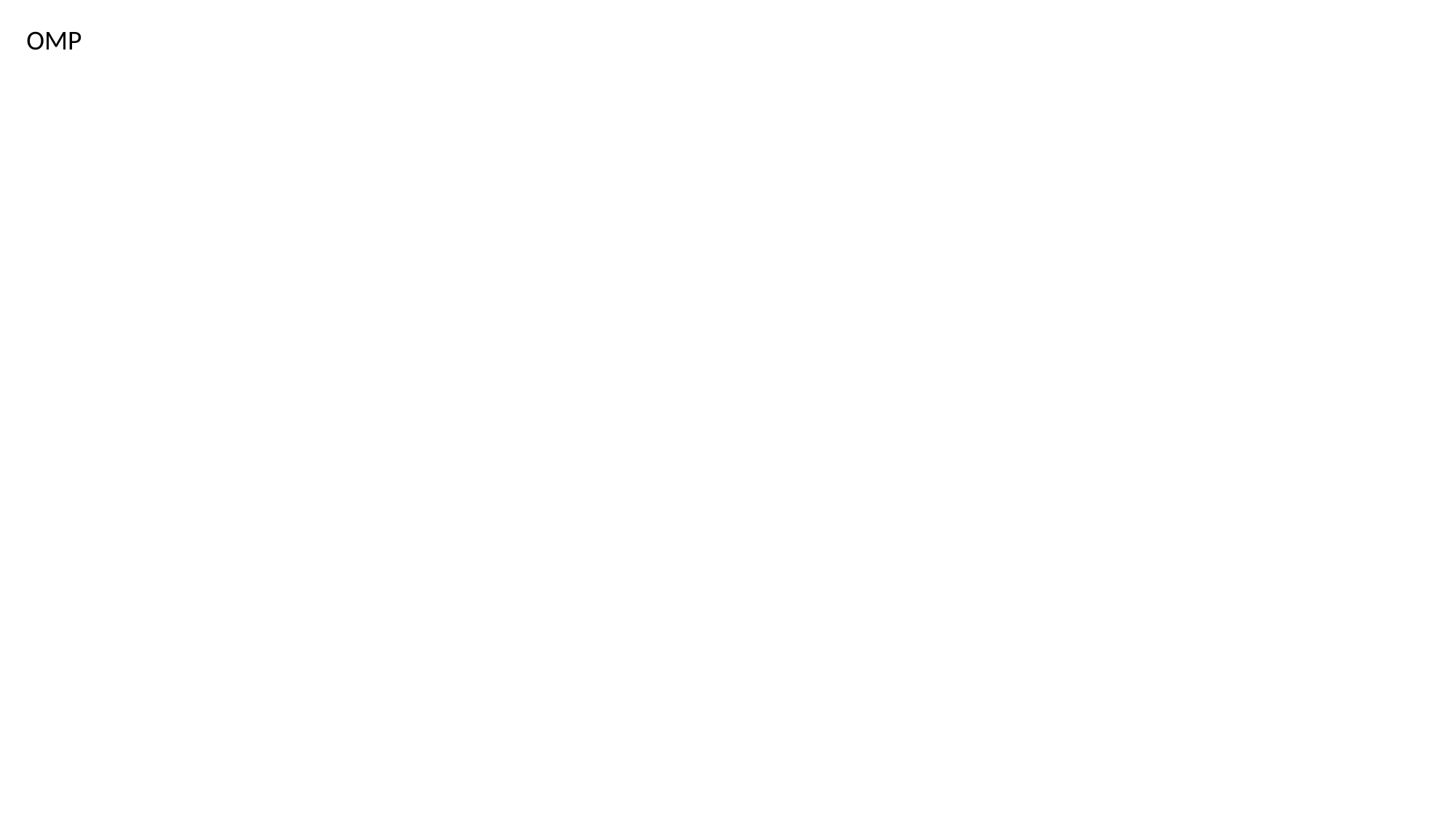

OMP

## Slide 18
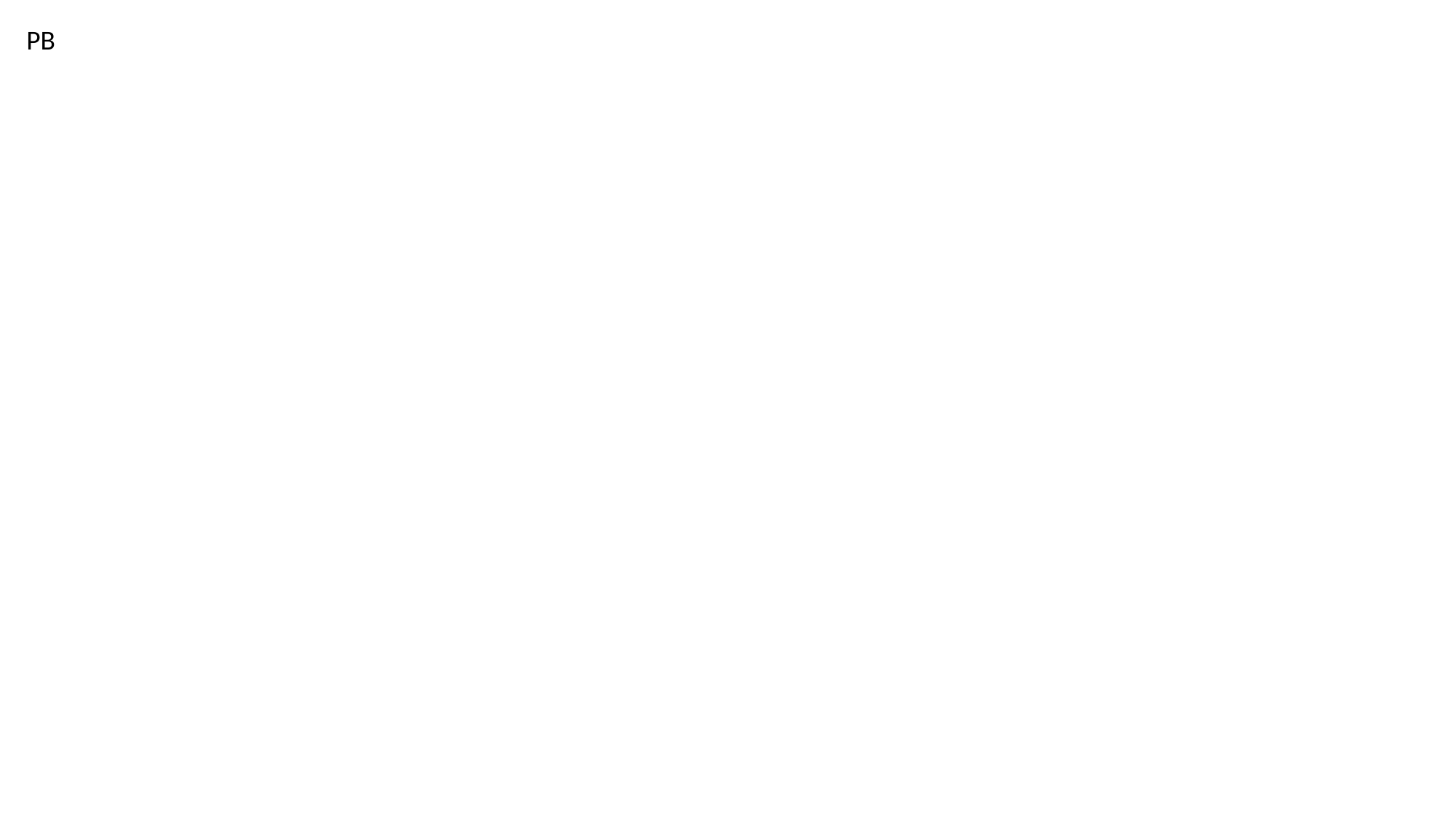

PB

## Slide 19
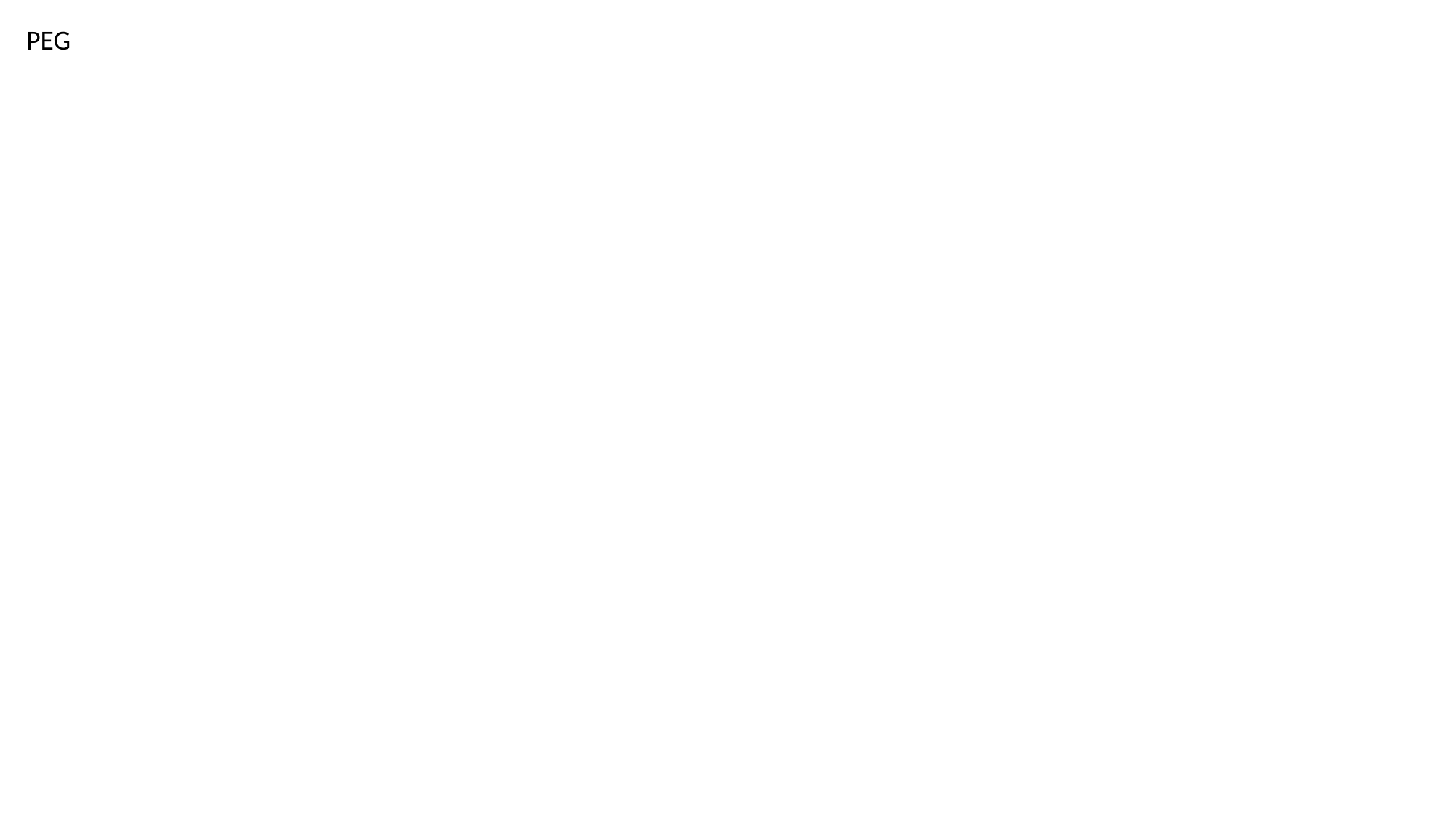

PEG

## Slide 20
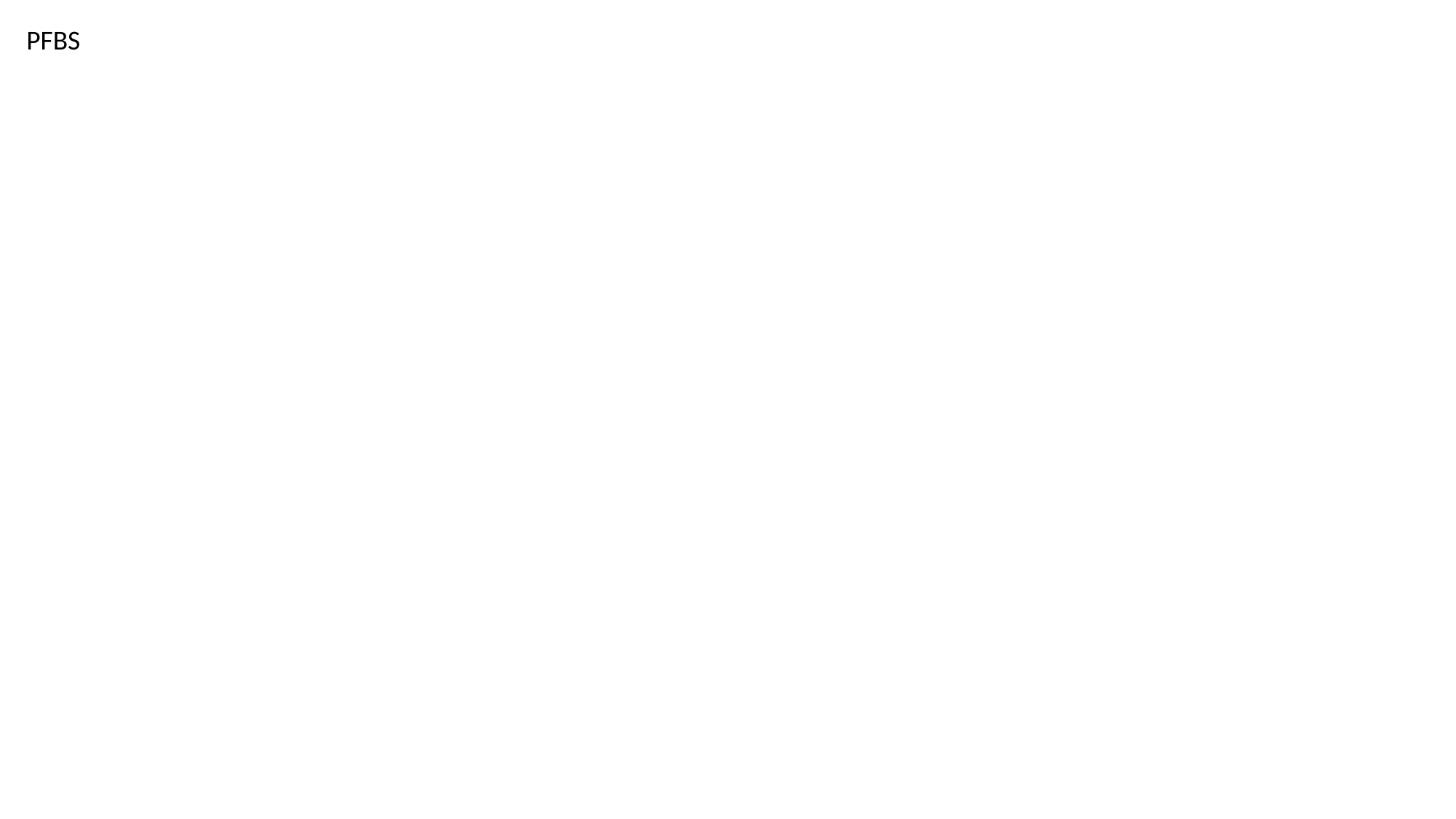

PFBS

## Slide 21
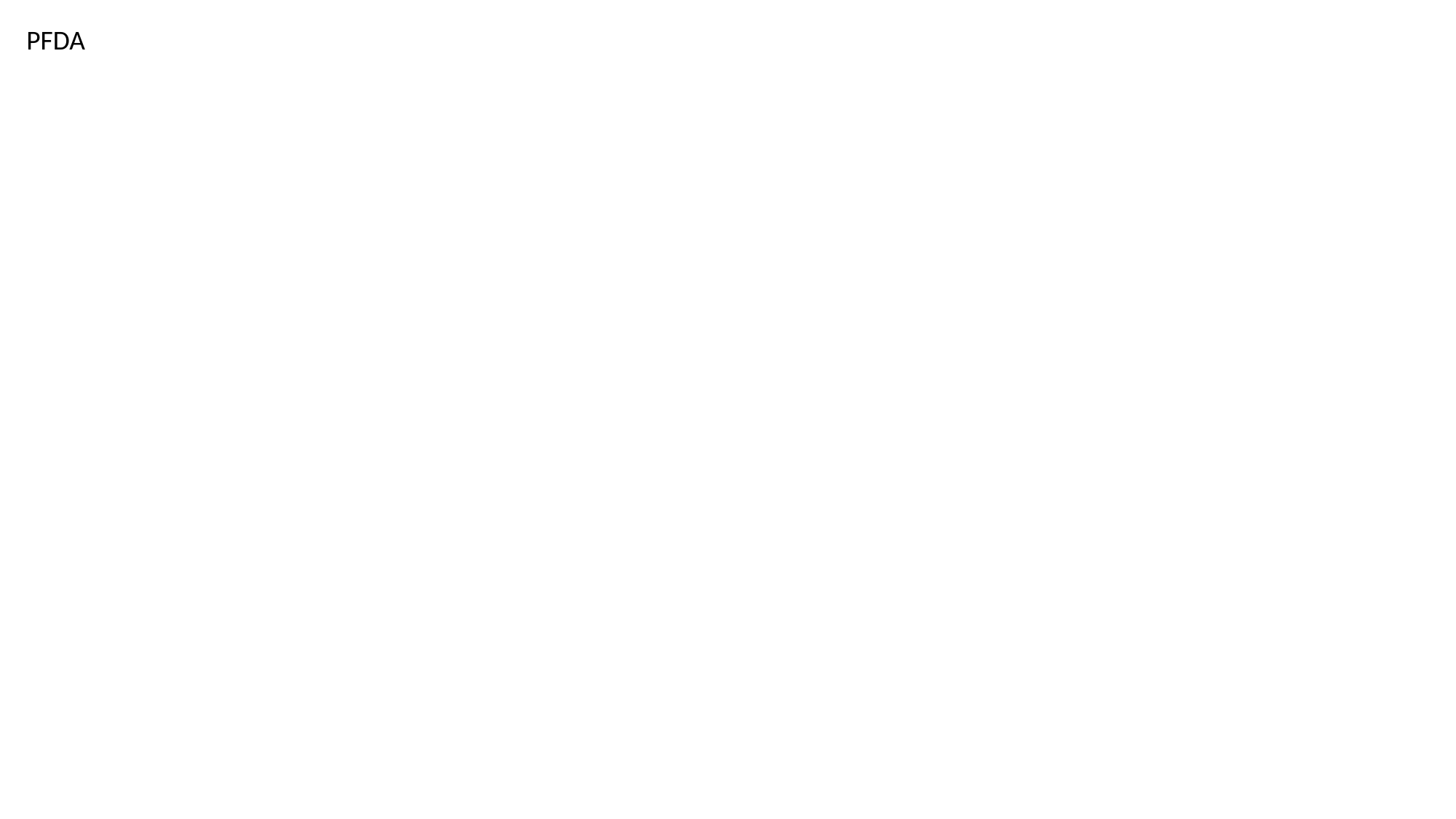

PFDA

## Slide 22
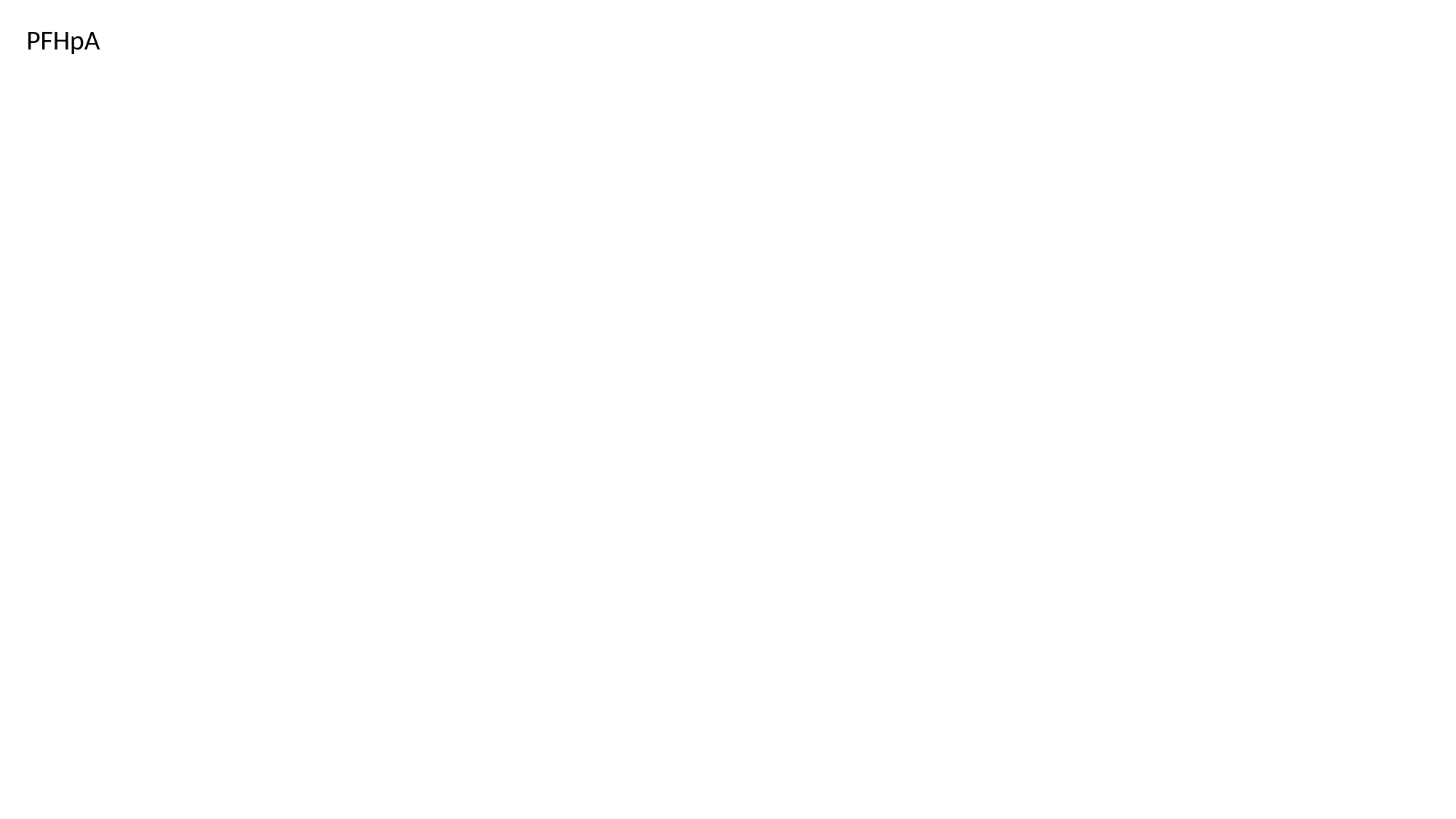

PFHpA

## Slide 23
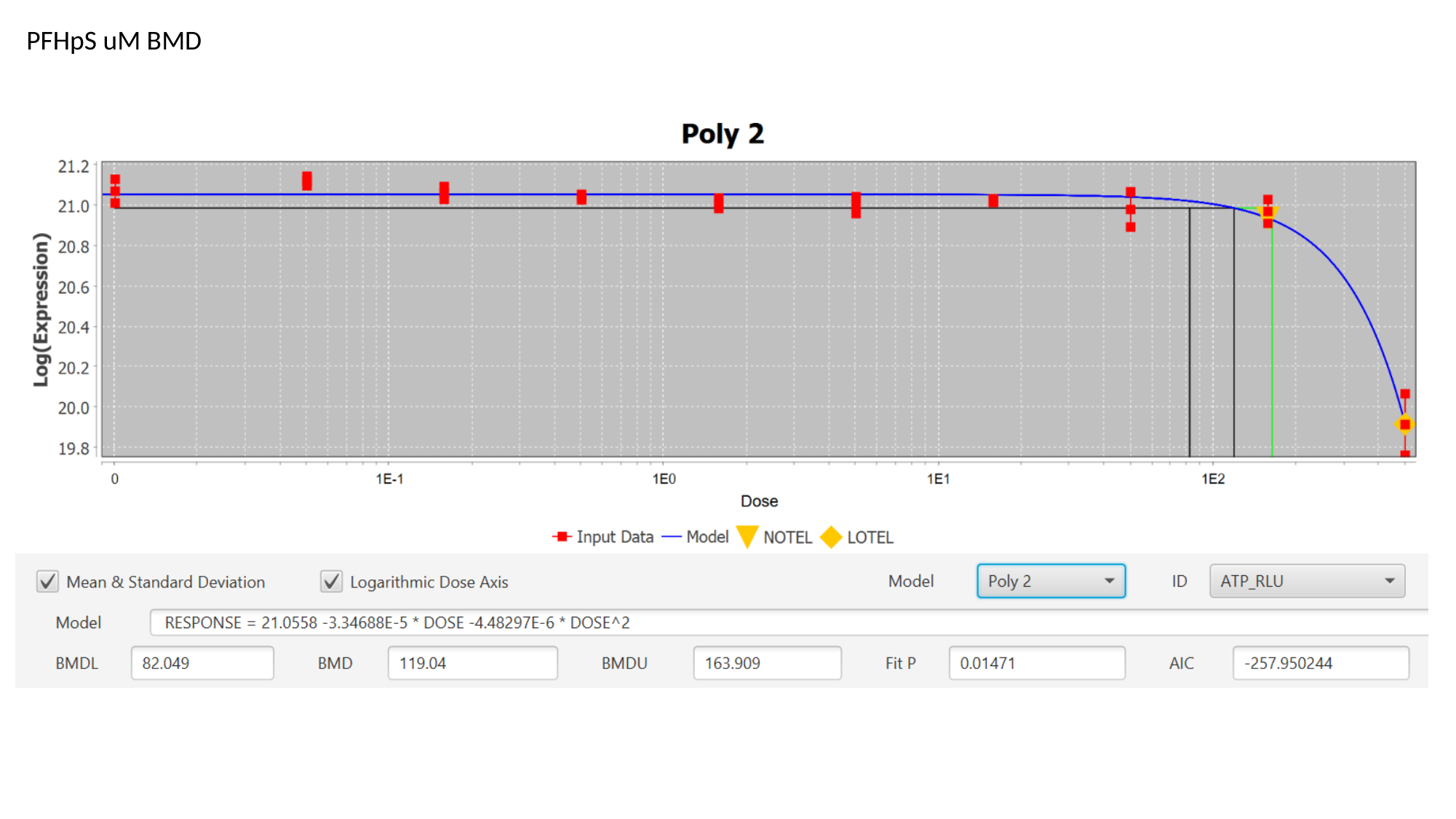

PFHpS uM BMD

## Slide 24
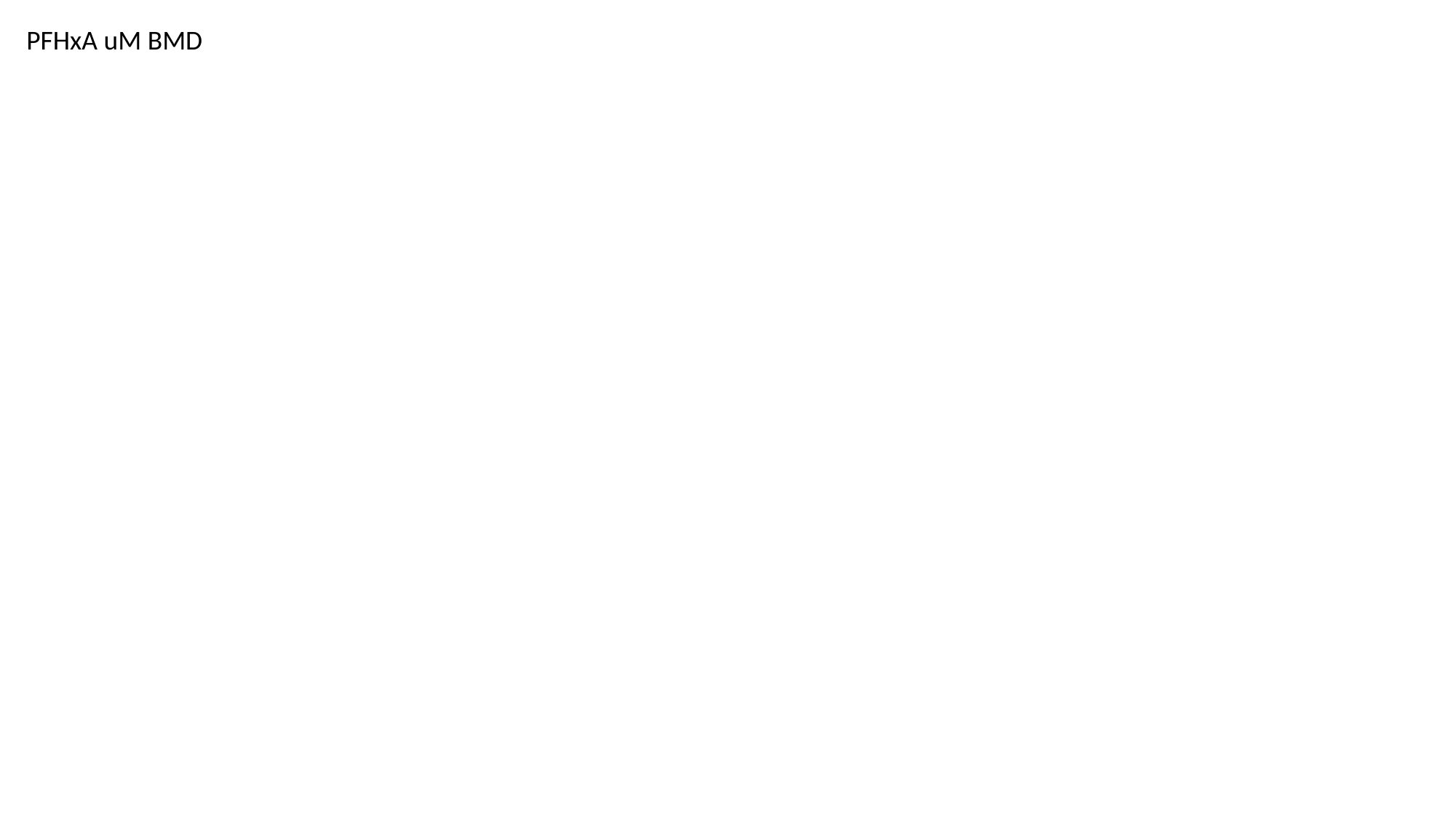

PFHxA uM BMD

## Slide 25
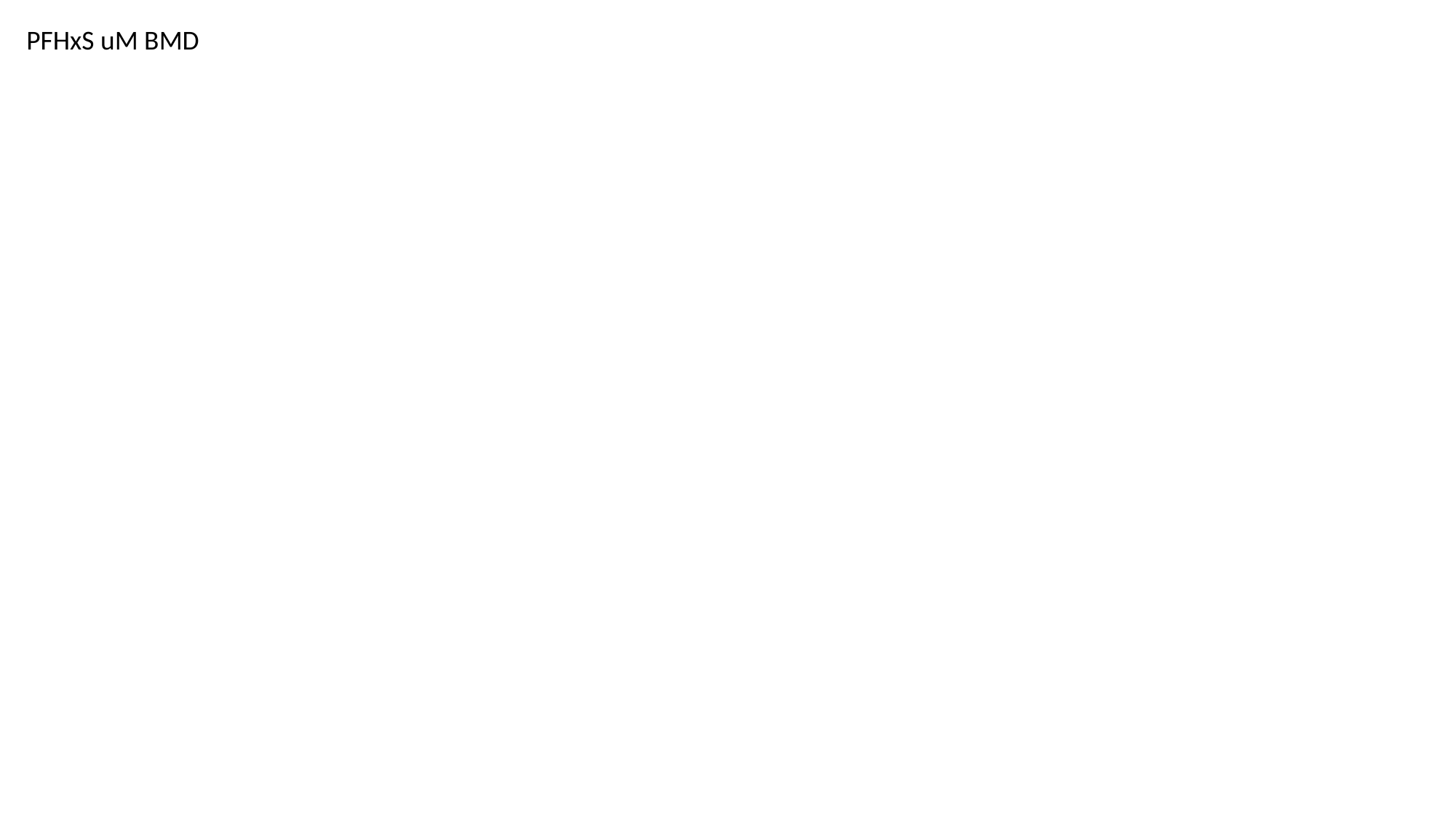

PFHxS uM BMD

## Slide 26
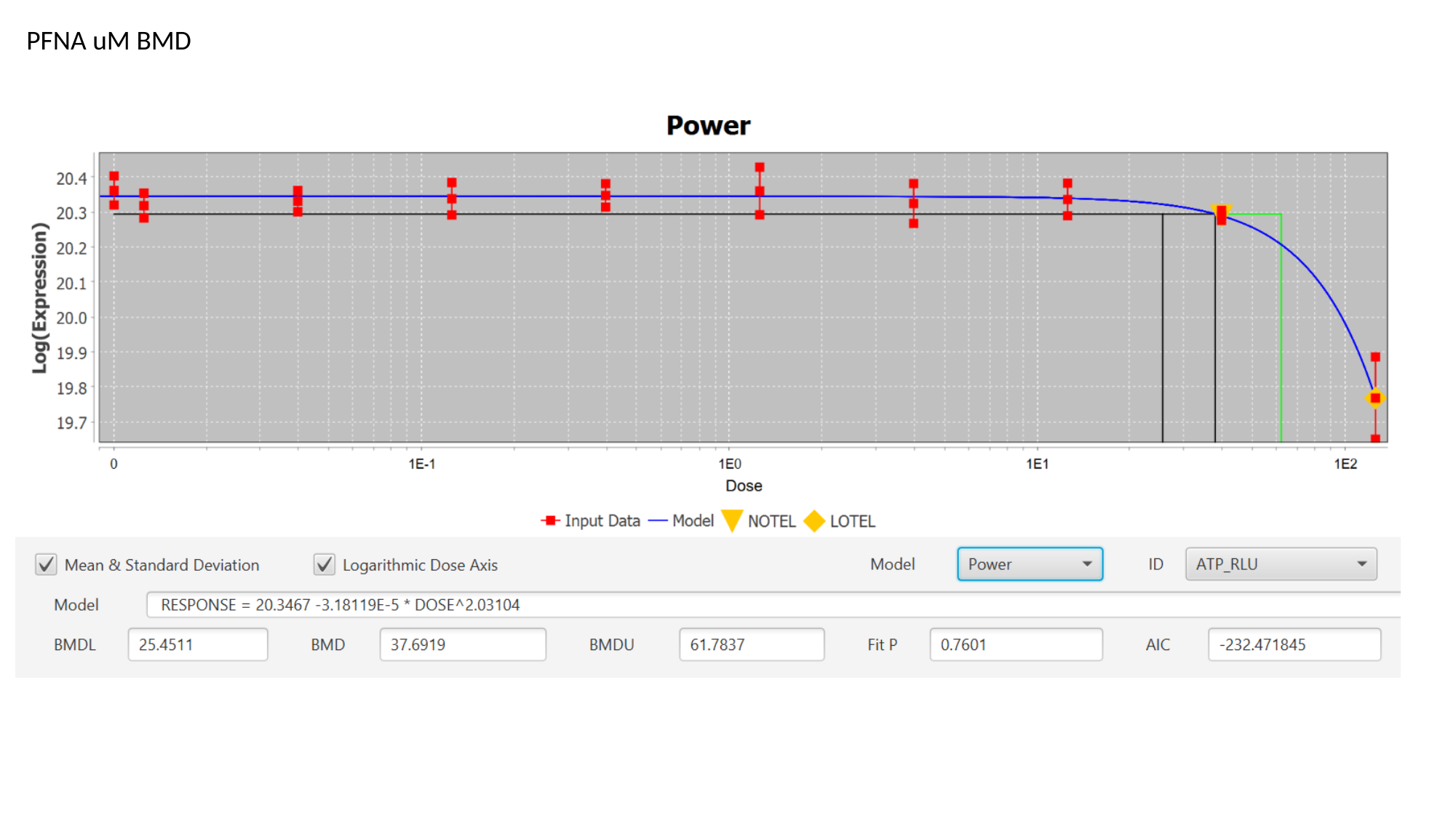

PFNA uM BMD

## Slide 27
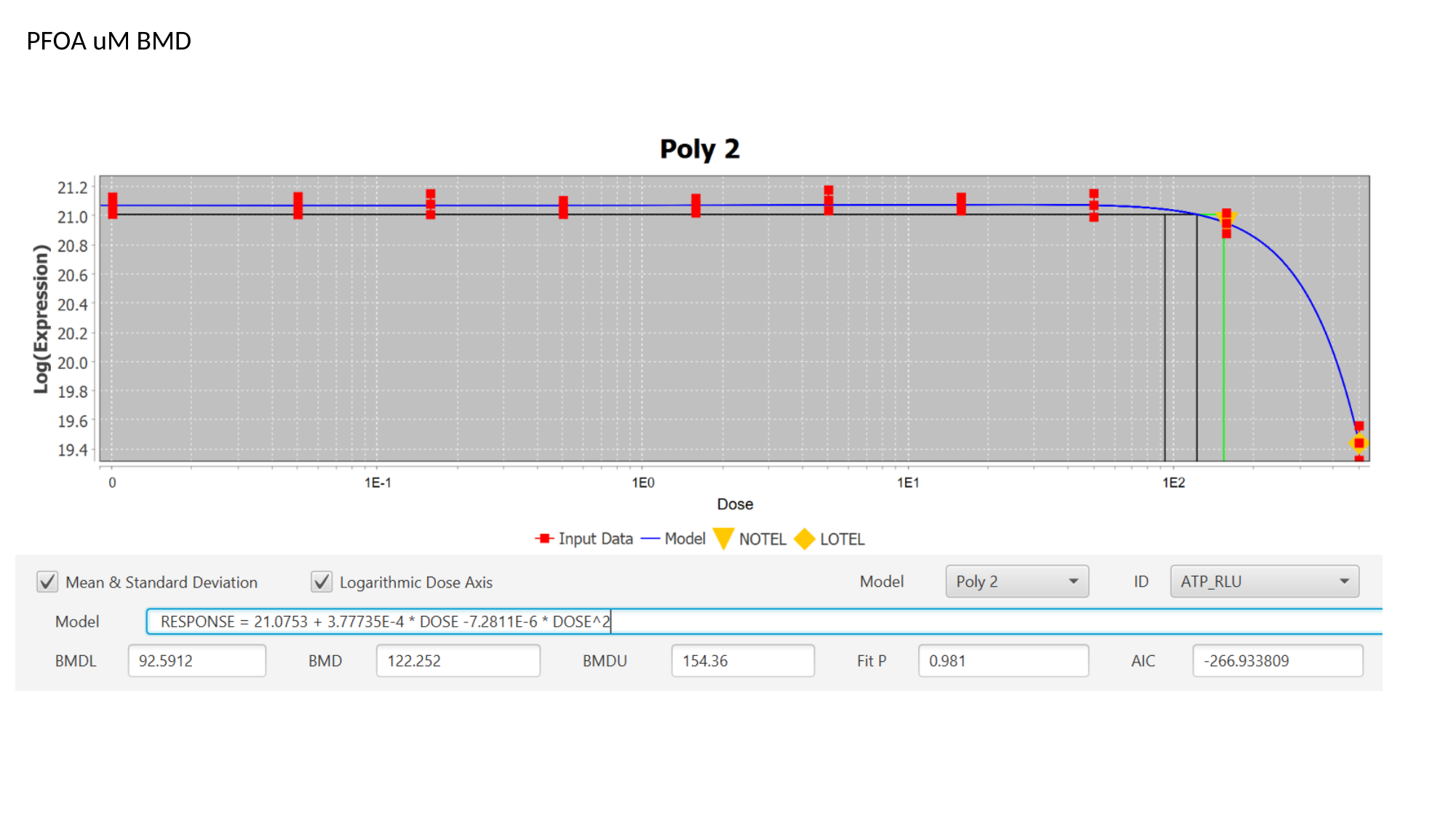

PFOA uM BMD

## Slide 28
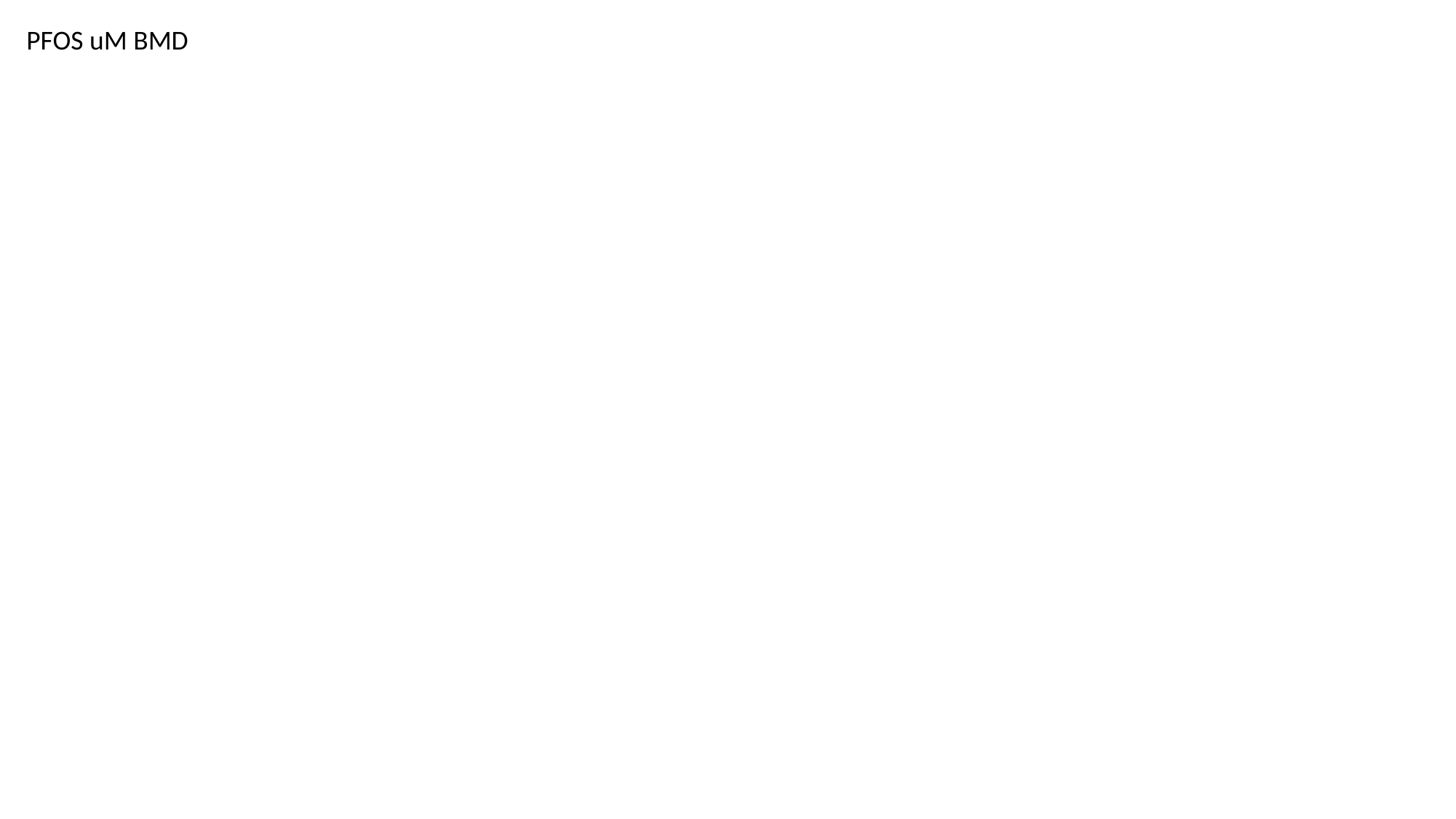

PFOS uM BMD

## Slide 29
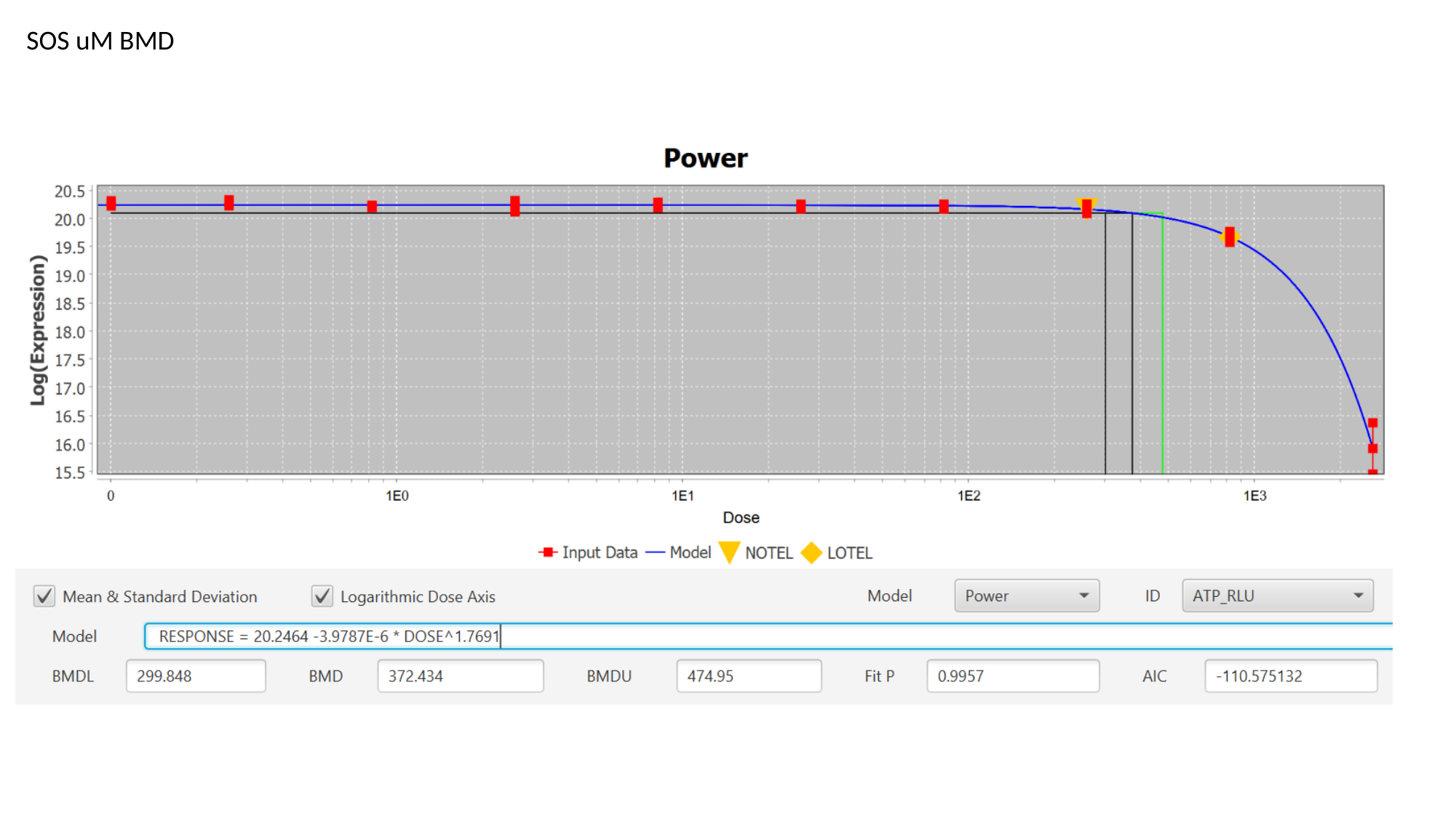

SOS uM BMD
