# Supplementary figures and images for: Unraveling Human Hepatocellular Responses to PFAS and Aqueous Film-Forming Foams (AFFFs) for Molecular Hazard Prioritization and In Vivo Translation
Source: Environ Sci Technol. 2025 Feb 2;59(5):2423–35. doi: 10.1021/acs.est.4c10595 (PMC11823446; doi:10.1021/acs.est.4c10595)

## Slide 1
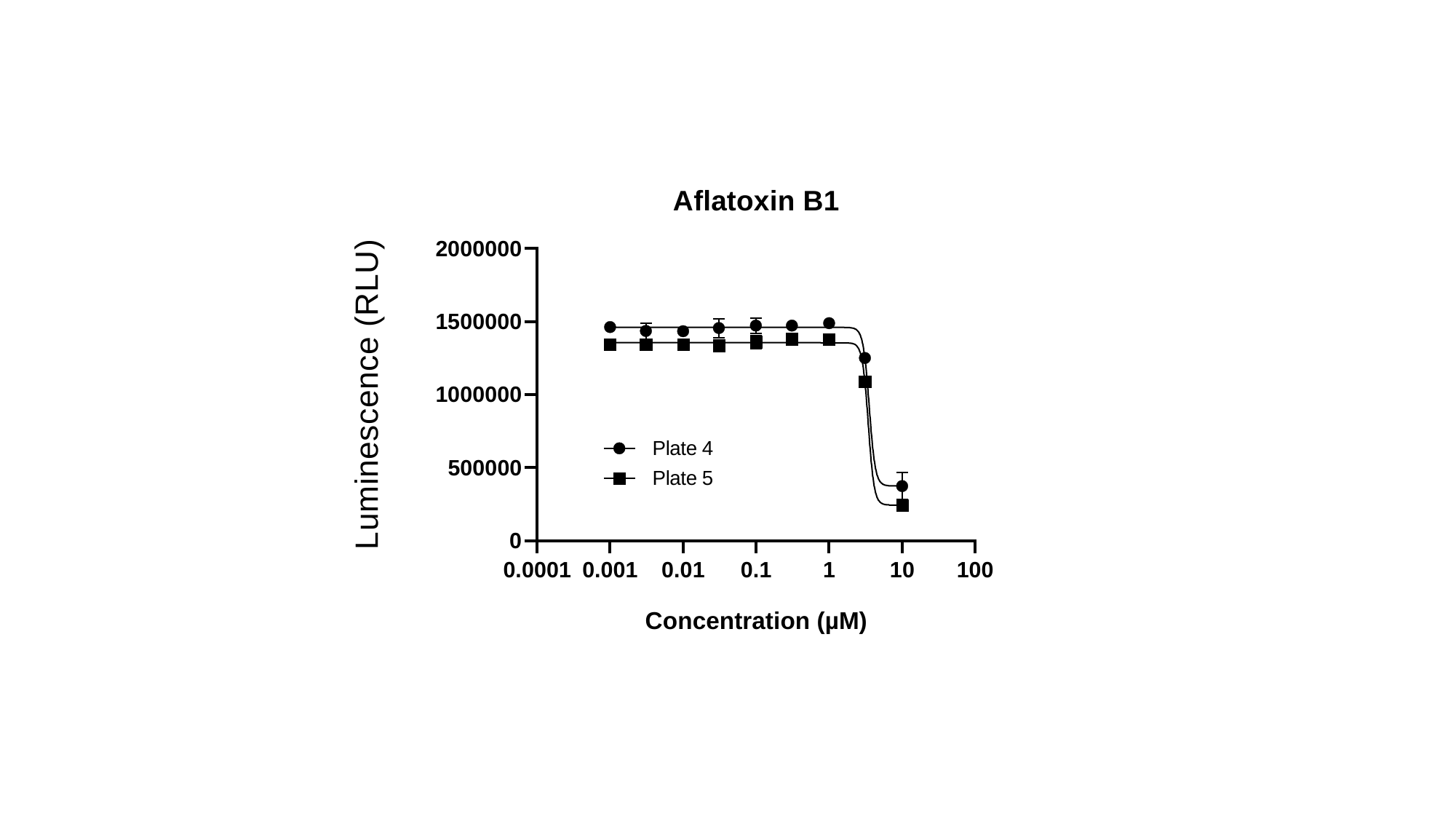

Supplement: Supplementary file 8 — es4c10595_si_008.zip [file es4c10595_si_008.zip › Analyzed Data Files/Aflatoxin B1 Positive Control/Aflatoxin B1 Supplemental Figure Image.pptx]

## Slide 1
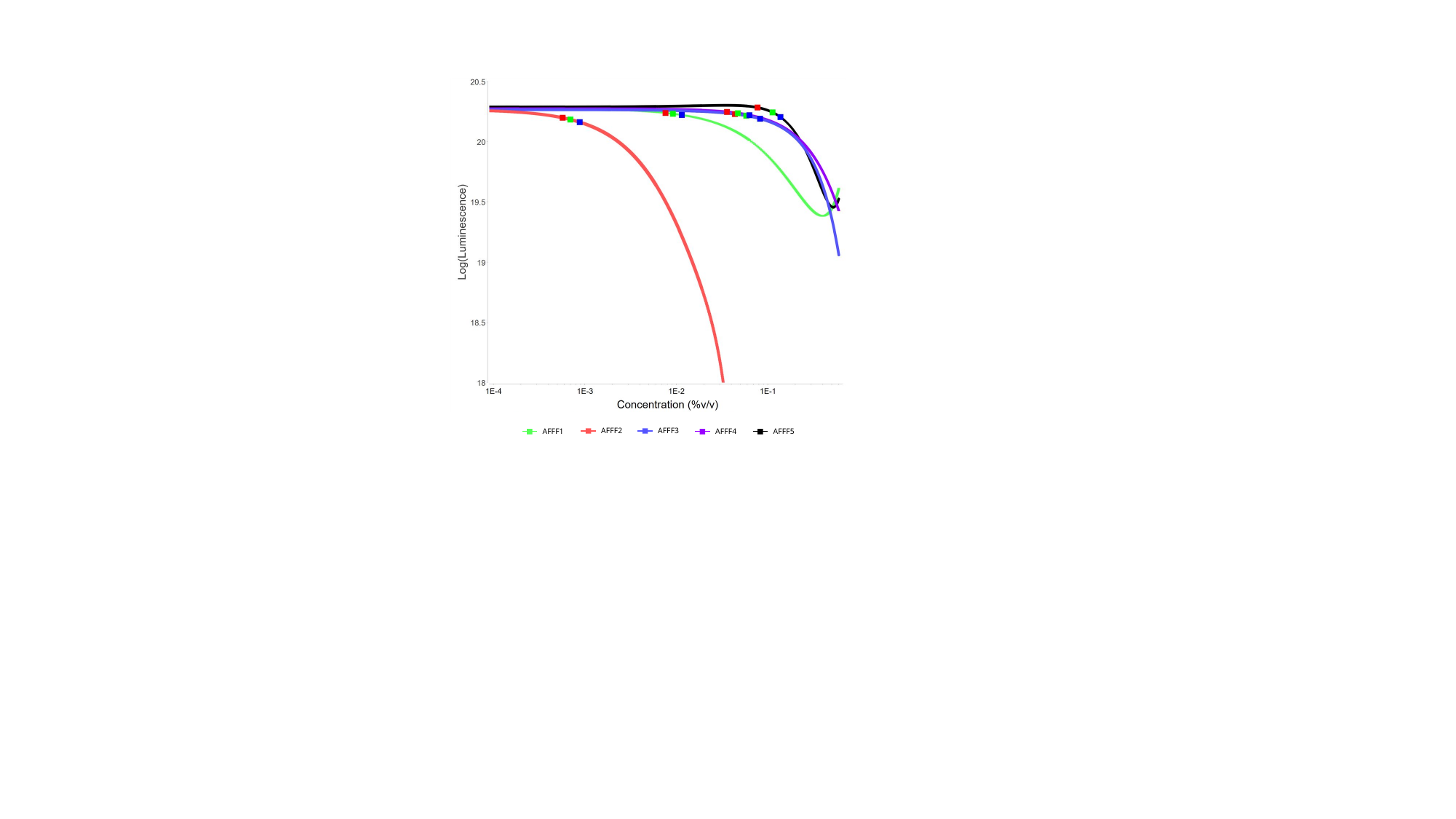

AFFF2
AFFF3
AFFF1
AFFF4
AFFF5

Supplement: Supplementary file 8 — es4c10595_si_008.zip [file es4c10595_si_008.zip › Analyzed Data Files/ATP Depletion Curve Overlays/ATP AFFF BMC Overlay with larger labels.pptx]

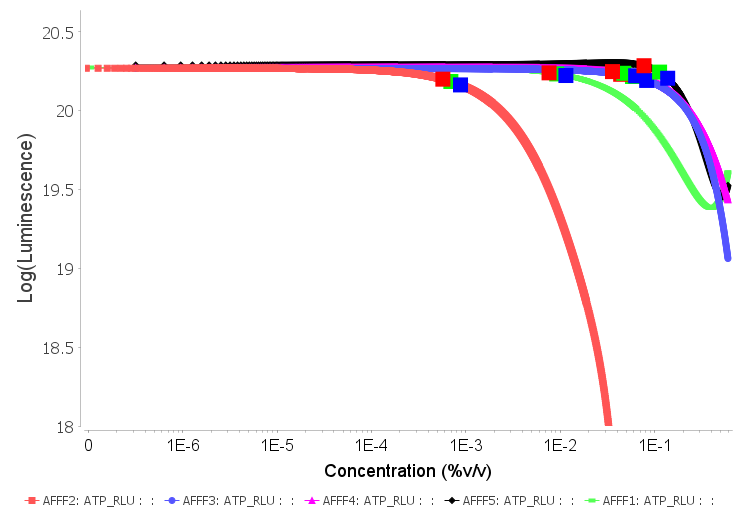

Supplement: Supplementary file 8 — es4c10595_si_008.zip [file es4c10595_si_008.zip › Analyzed Data Files/ATP Depletion Curve Overlays/ATP_AFFF_BMC_Curve_Overlays.png]

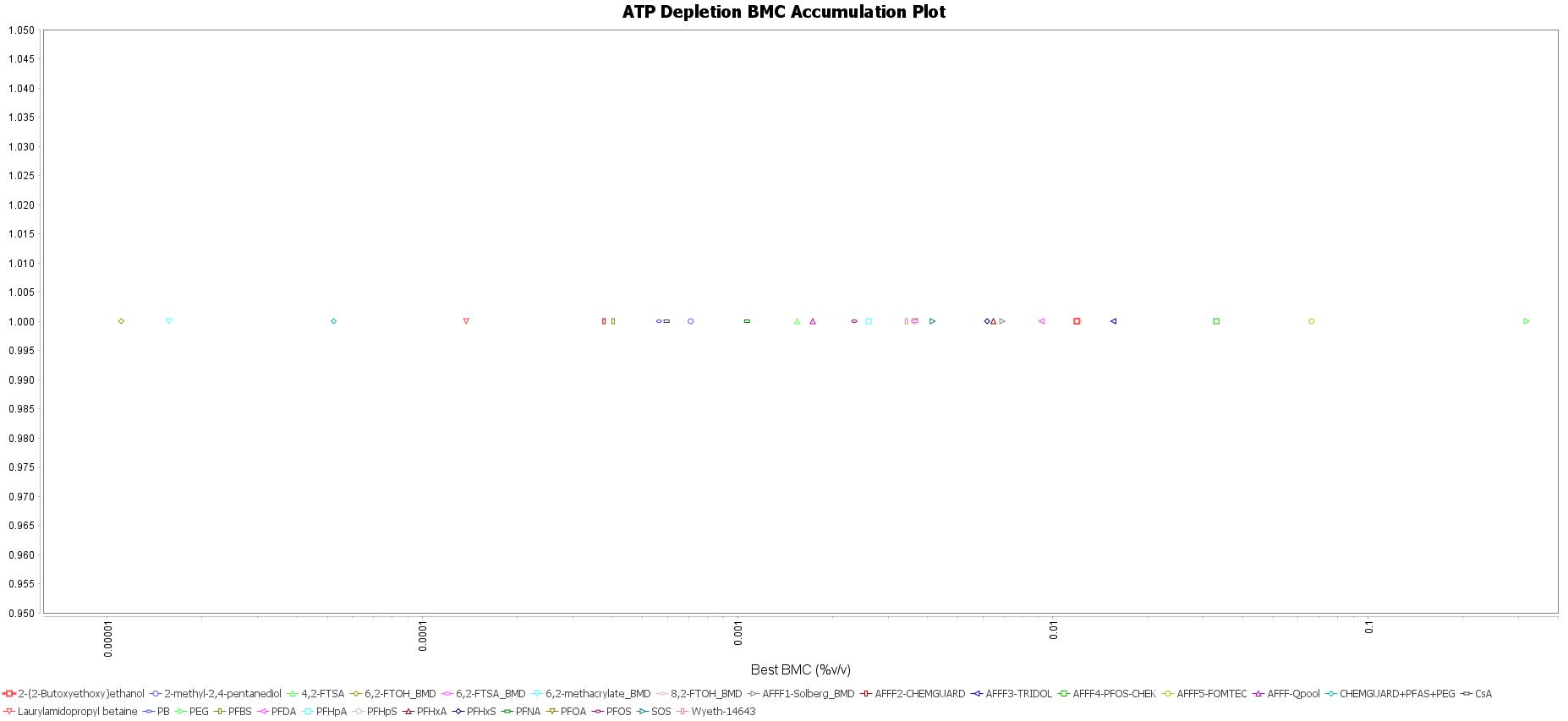

Supplement: Supplementary file 8 — es4c10595_si_008.zip [file es4c10595_si_008.zip › Analyzed Data Files/BMDExpress Analysis/BMC modeling input files/ATP Depletion BMCs from Dose Range Finding]

## Slide 1
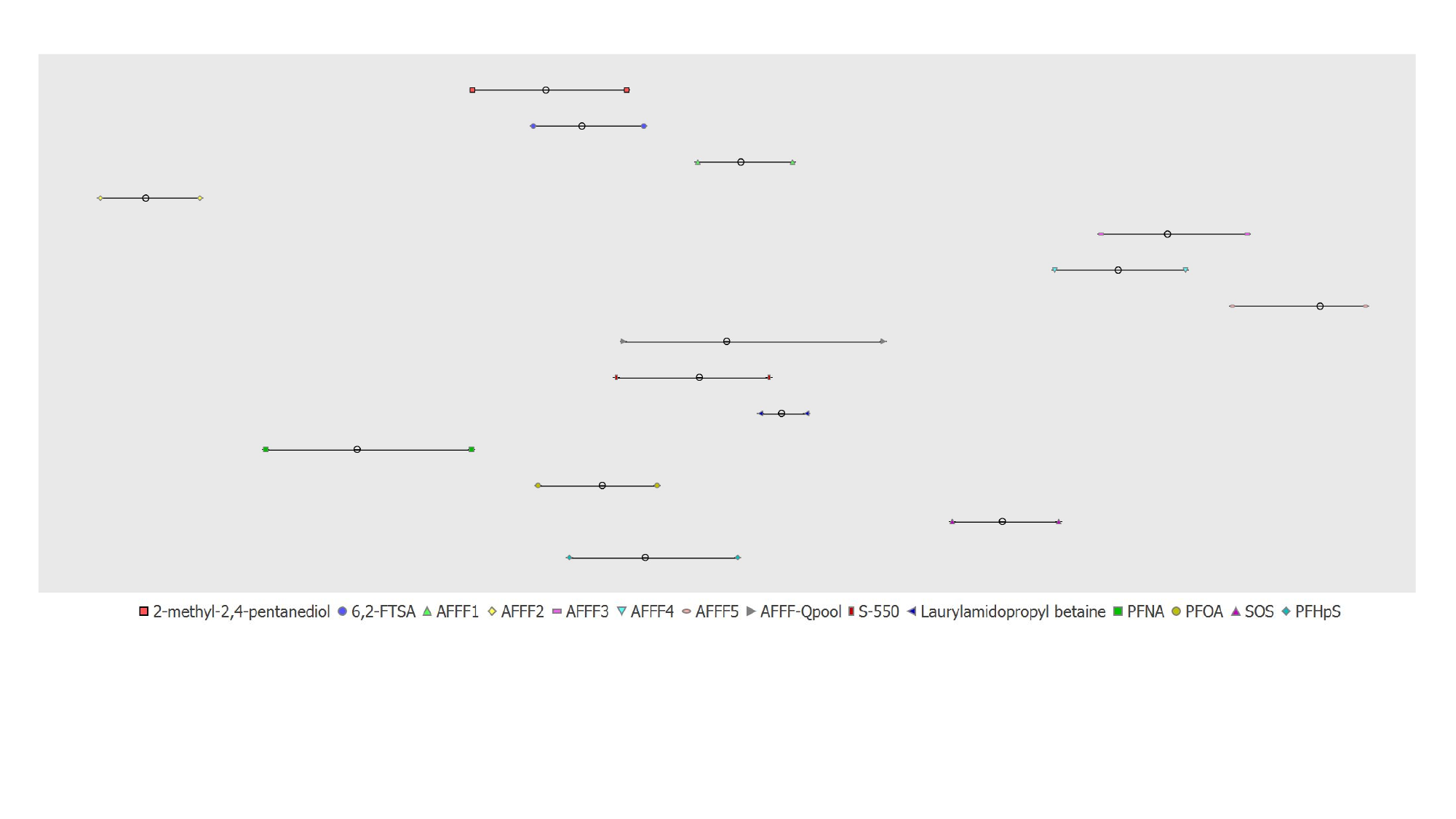

#

Supplement: Supplementary file 8 — es4c10595_si_008.zip [file es4c10595_si_008.zip › Analyzed Data Files/Range Plot All CTG Positive Figure.pptx]

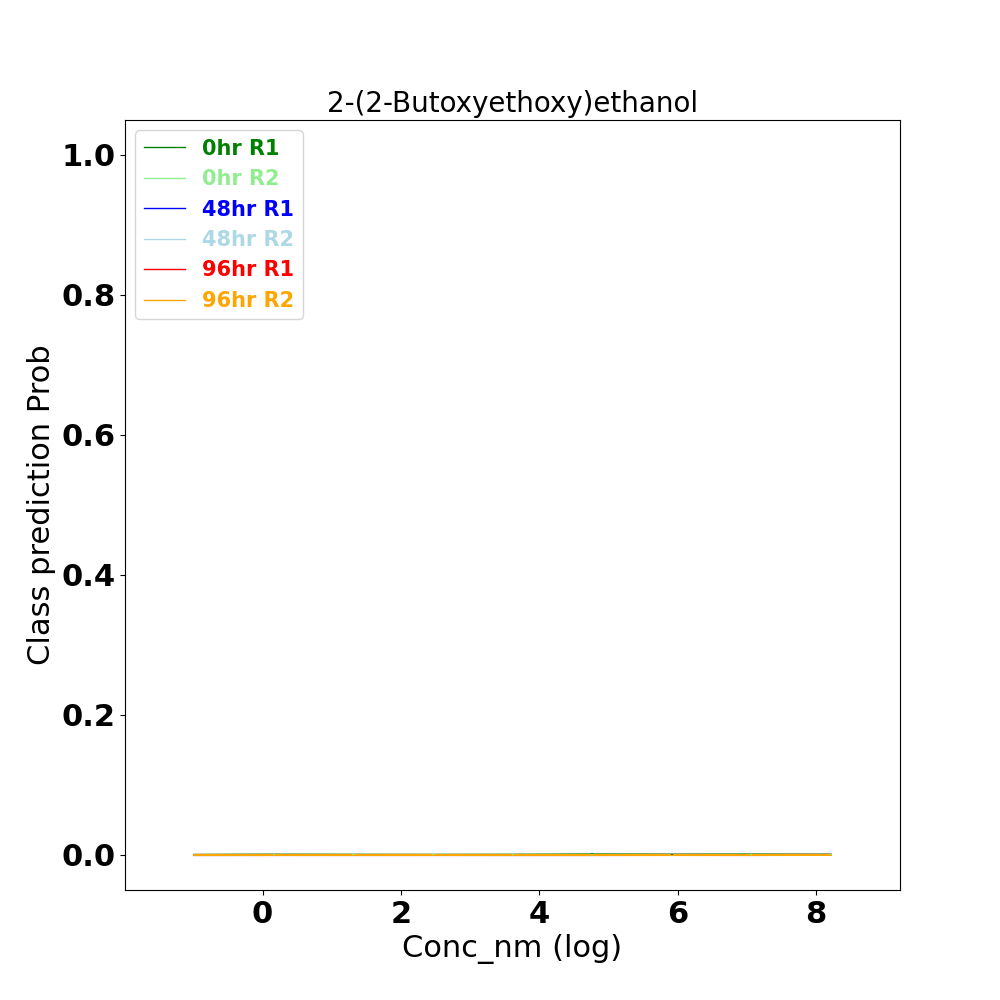

Supplement: Supplementary file 9 — es4c10595_si_009.zip [file es4c10595_si_009.zip › Cell Morphology Image Analysis-Sciome/Dose_Response_Plots/Dose-reponse-2-(2-Butoxyethoxy)ethanol.png]

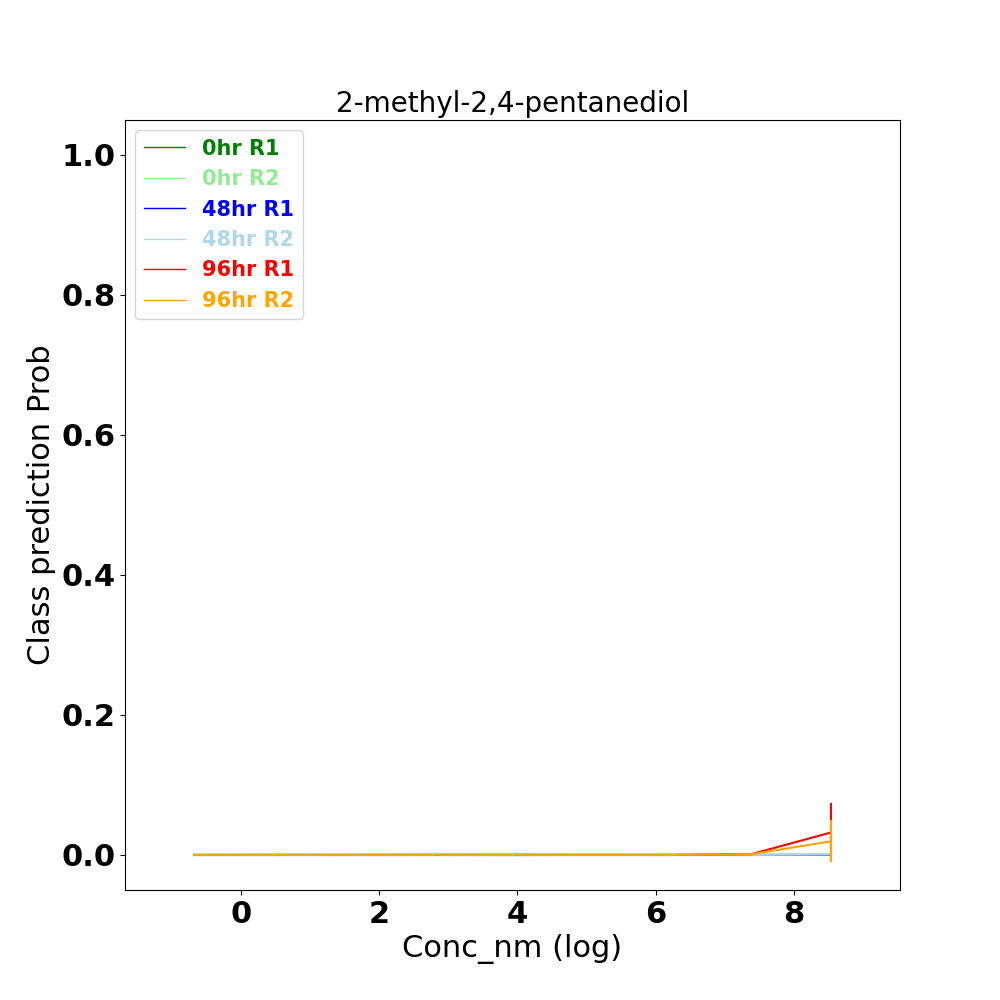

Supplement: Supplementary file 9 — es4c10595_si_009.zip [file es4c10595_si_009.zip › Cell Morphology Image Analysis-Sciome/Dose_Response_Plots/Dose-reponse-2-methyl-2,4-pentanediol.png]

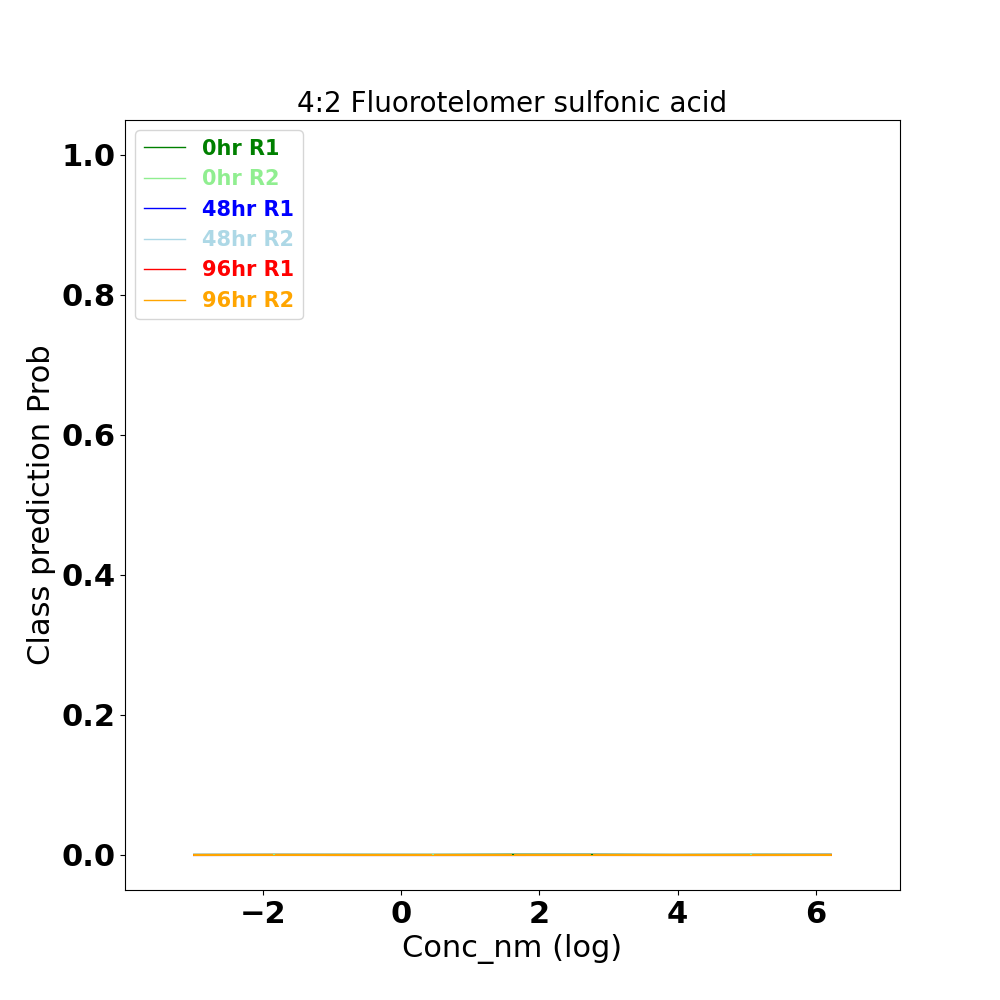

Supplement: Supplementary file 9 — es4c10595_si_009.zip [file es4c10595_si_009.zip › Cell Morphology Image Analysis-Sciome/Dose_Response_Plots/Dose-reponse-4%3A2 Fluorotelomer sulfonic acid.png]

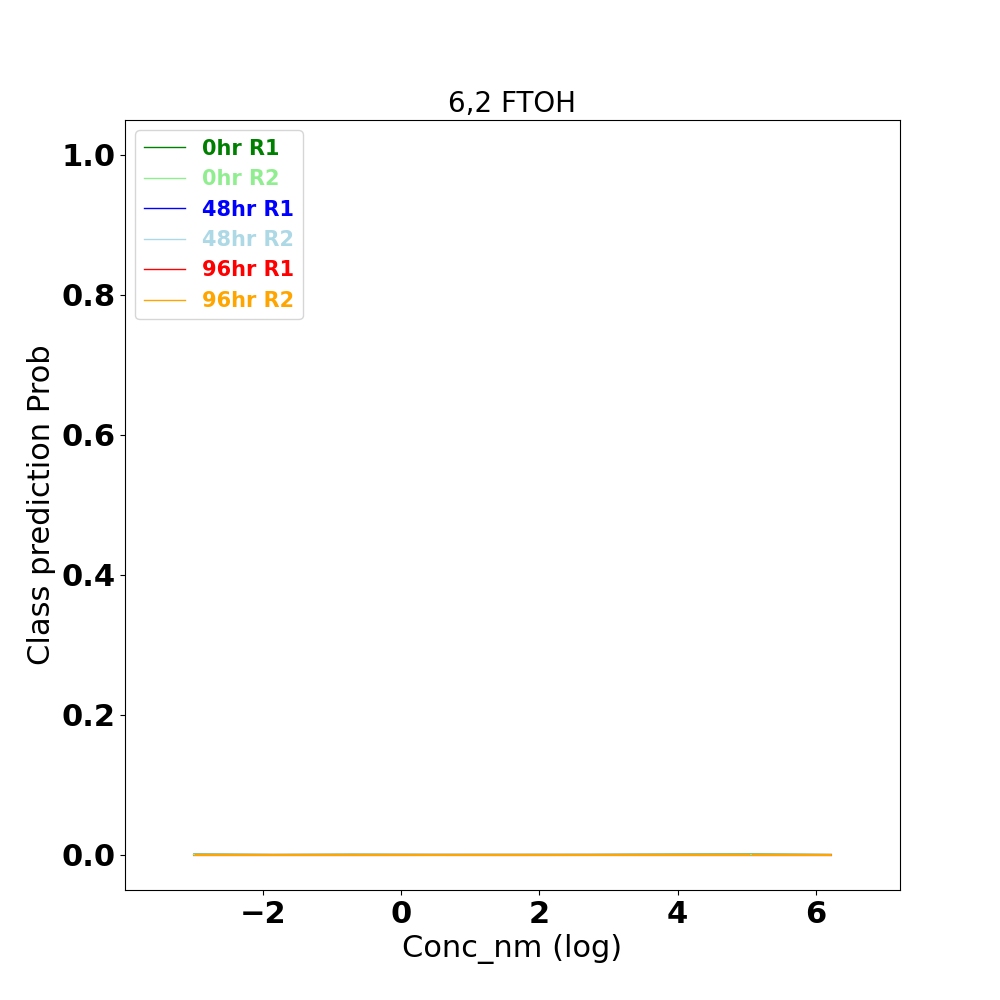

Supplement: Supplementary file 9 — es4c10595_si_009.zip [file es4c10595_si_009.zip › Cell Morphology Image Analysis-Sciome/Dose_Response_Plots/Dose-reponse-6,2 FTOH.png]

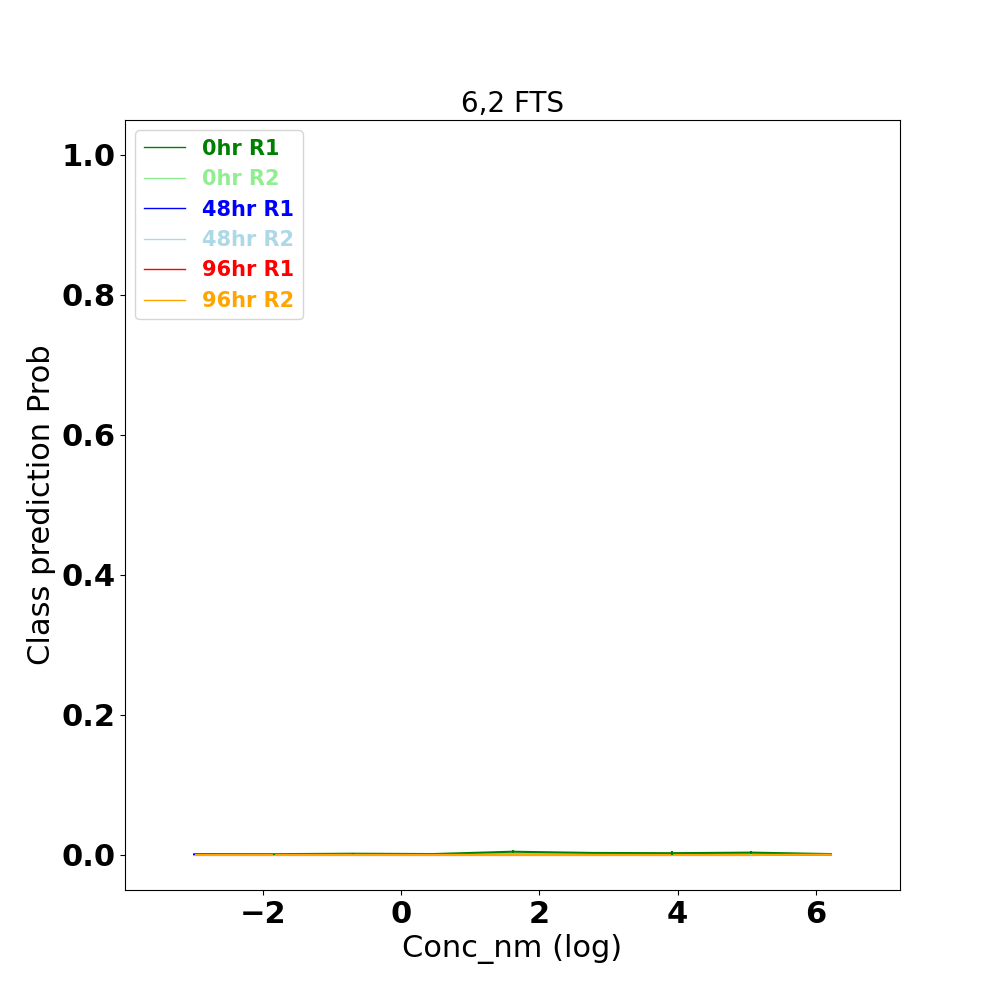

Supplement: Supplementary file 9 — es4c10595_si_009.zip [file es4c10595_si_009.zip › Cell Morphology Image Analysis-Sciome/Dose_Response_Plots/Dose-reponse-6,2 FTS.png]

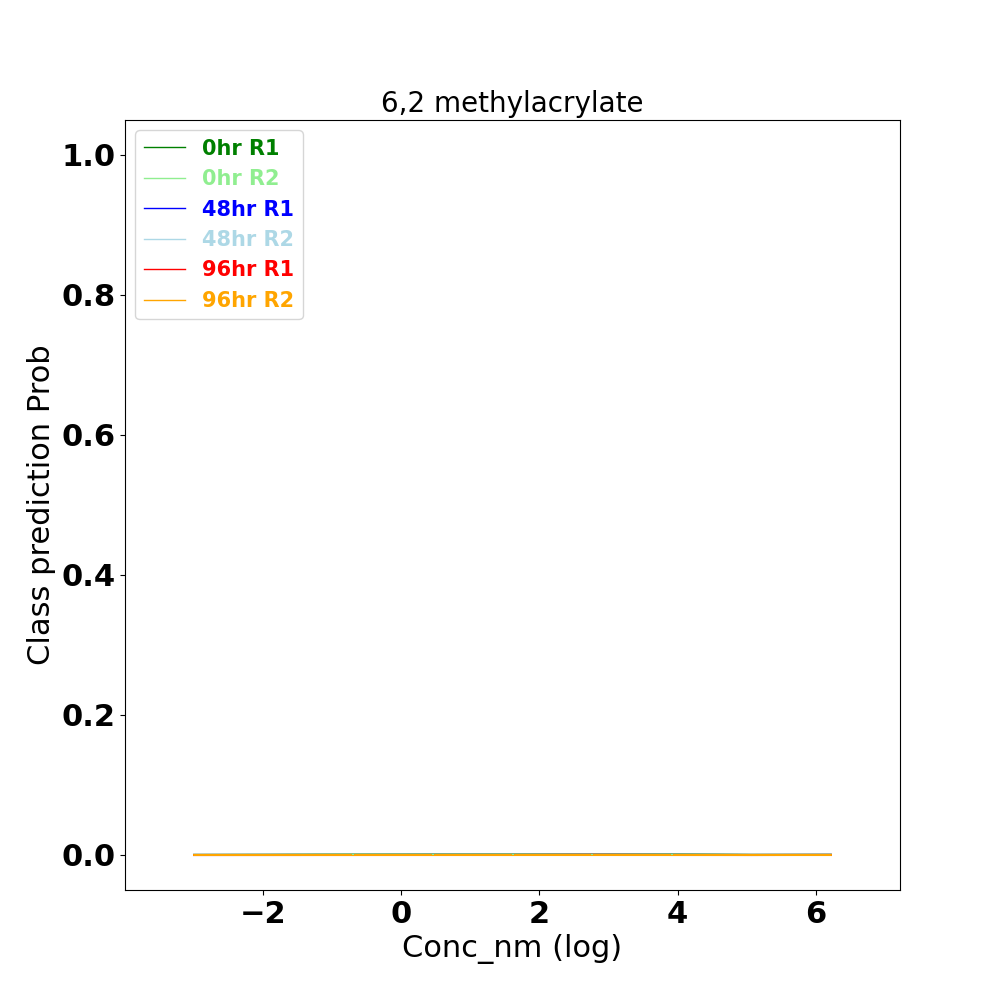

Supplement: Supplementary file 9 — es4c10595_si_009.zip [file es4c10595_si_009.zip › Cell Morphology Image Analysis-Sciome/Dose_Response_Plots/Dose-reponse-6,2 methylacrylate.png]

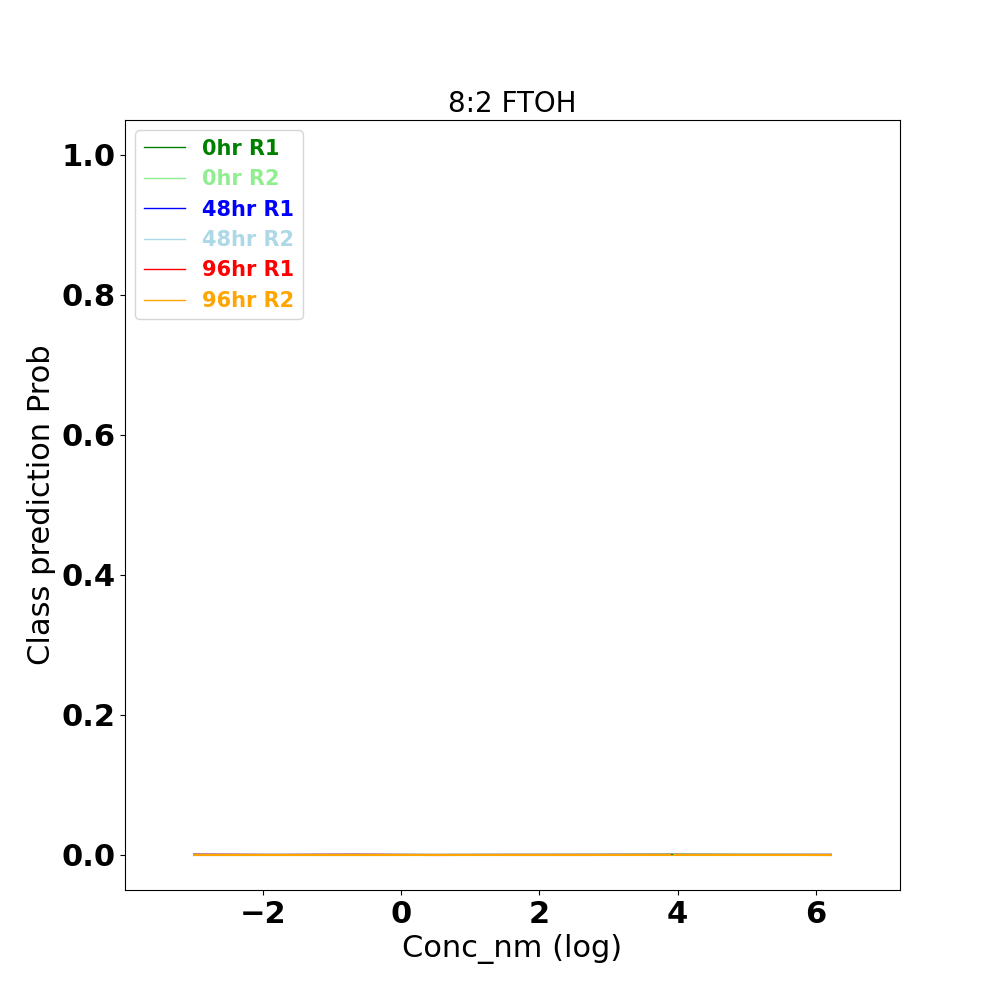

Supplement: Supplementary file 9 — es4c10595_si_009.zip [file es4c10595_si_009.zip › Cell Morphology Image Analysis-Sciome/Dose_Response_Plots/Dose-reponse-8%3A2 FTOH.png]

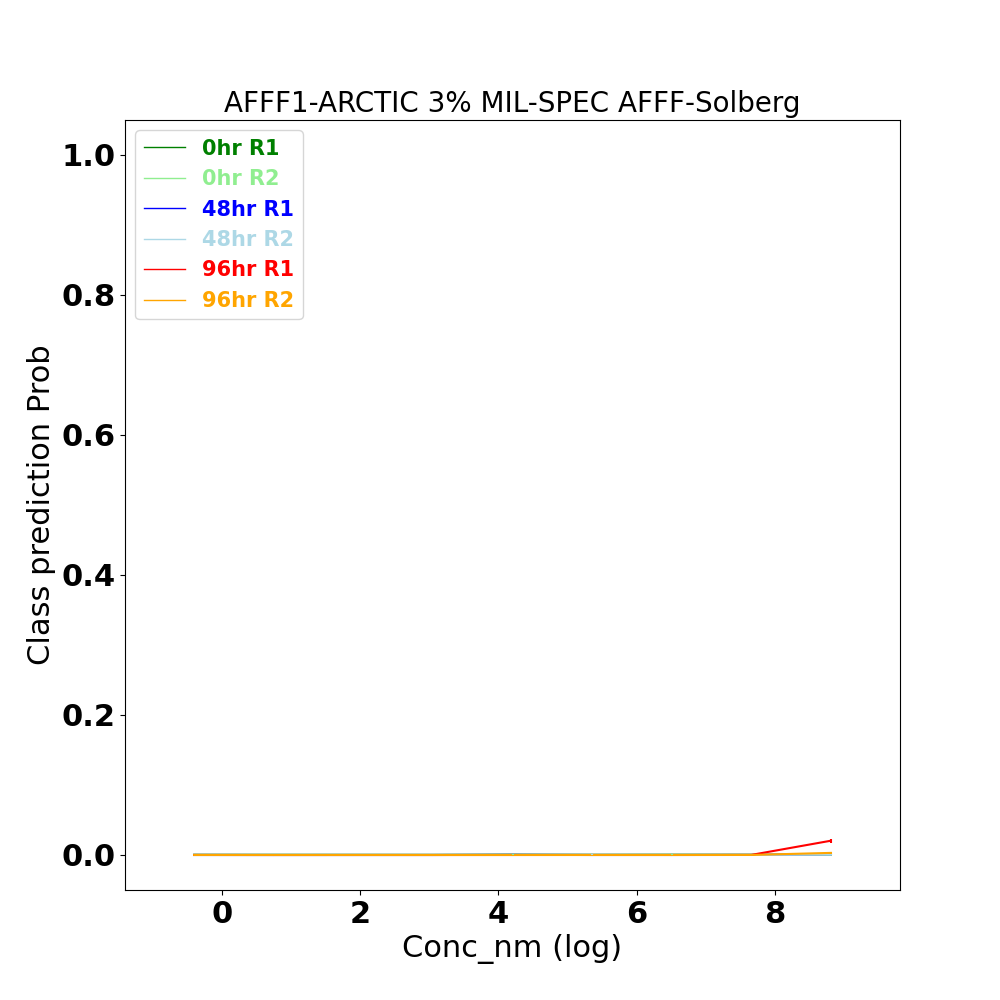

Supplement: Supplementary file 9 — es4c10595_si_009.zip [file es4c10595_si_009.zip › Cell Morphology Image Analysis-Sciome/Dose_Response_Plots/Dose-reponse-AFFF1-ARCTIC 3% MIL-SPEC AFFF-Solberg.png]

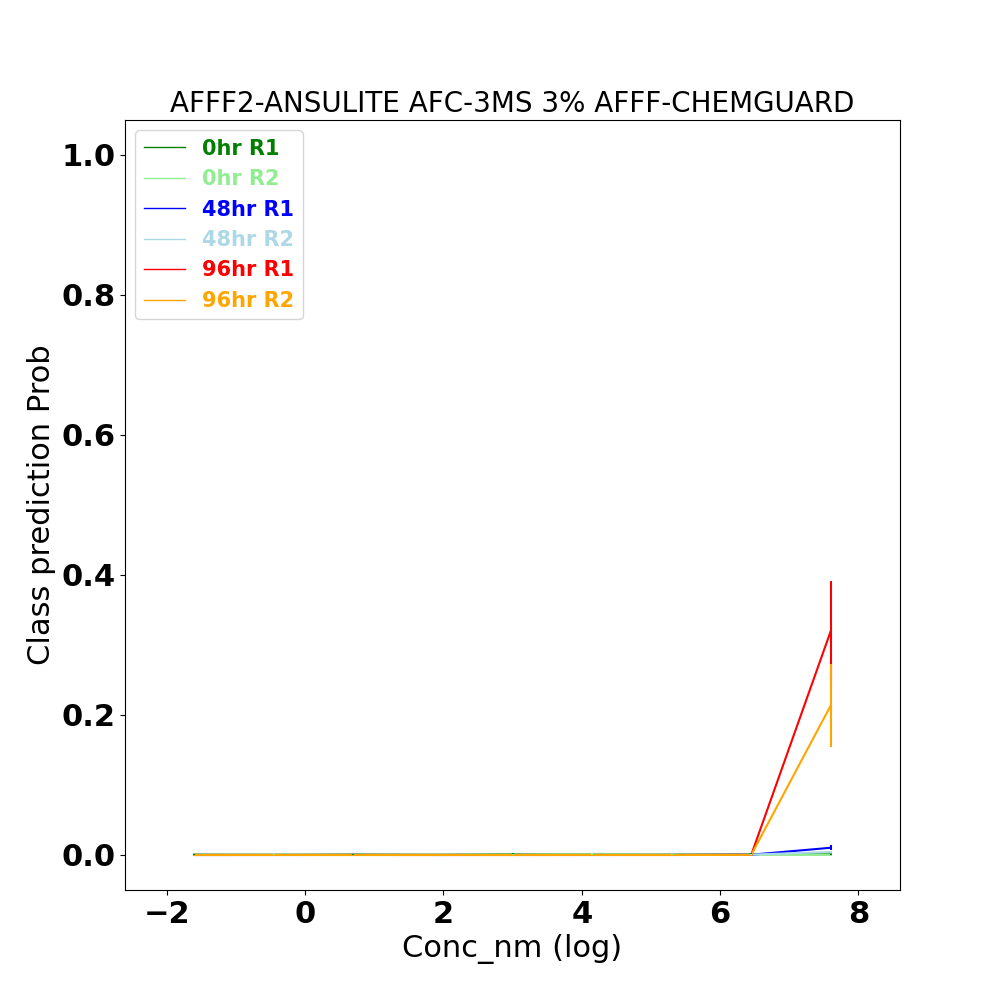

Supplement: Supplementary file 9 — es4c10595_si_009.zip [file es4c10595_si_009.zip › Cell Morphology Image Analysis-Sciome/Dose_Response_Plots/Dose-reponse-AFFF2-ANSULITE AFC-3MS 3% AFFF-CHEMGUARD.png]

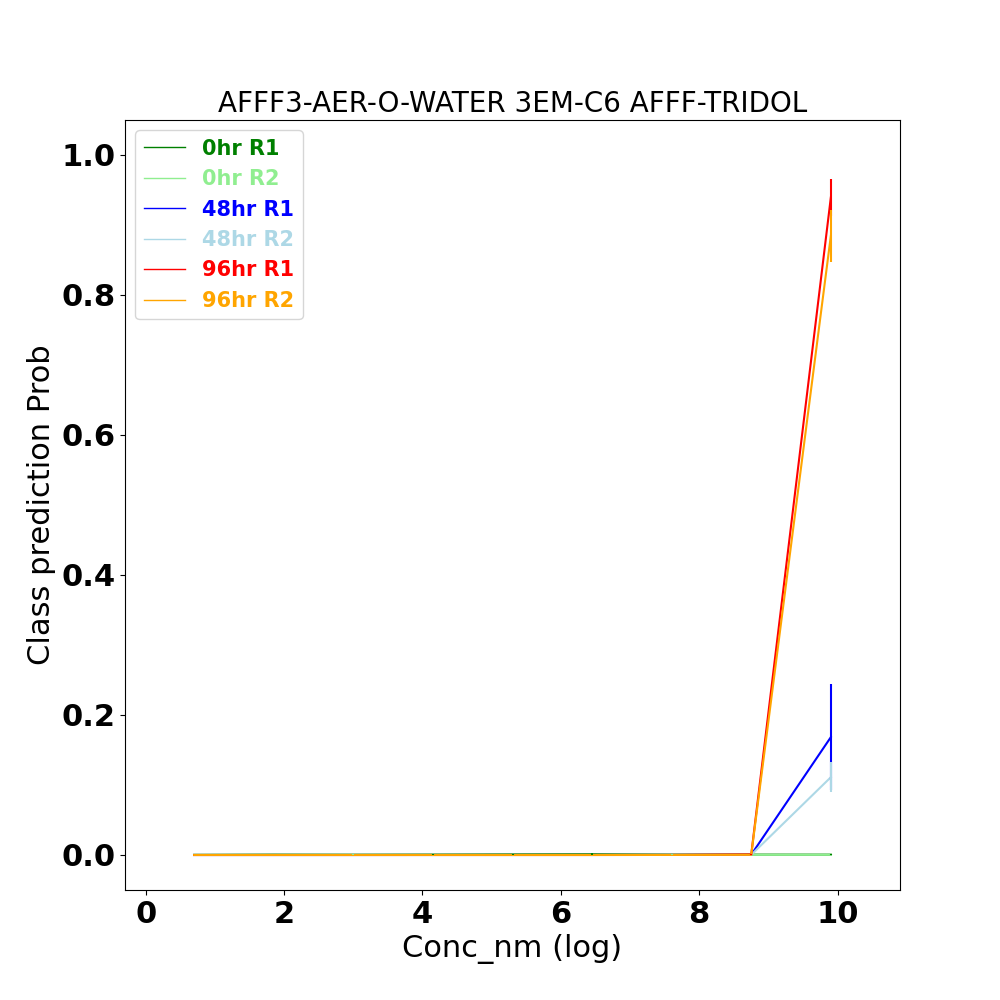

Supplement: Supplementary file 9 — es4c10595_si_009.zip [file es4c10595_si_009.zip › Cell Morphology Image Analysis-Sciome/Dose_Response_Plots/Dose-reponse-AFFF3-AER-O-WATER 3EM-C6 AFFF-TRIDOL.png]

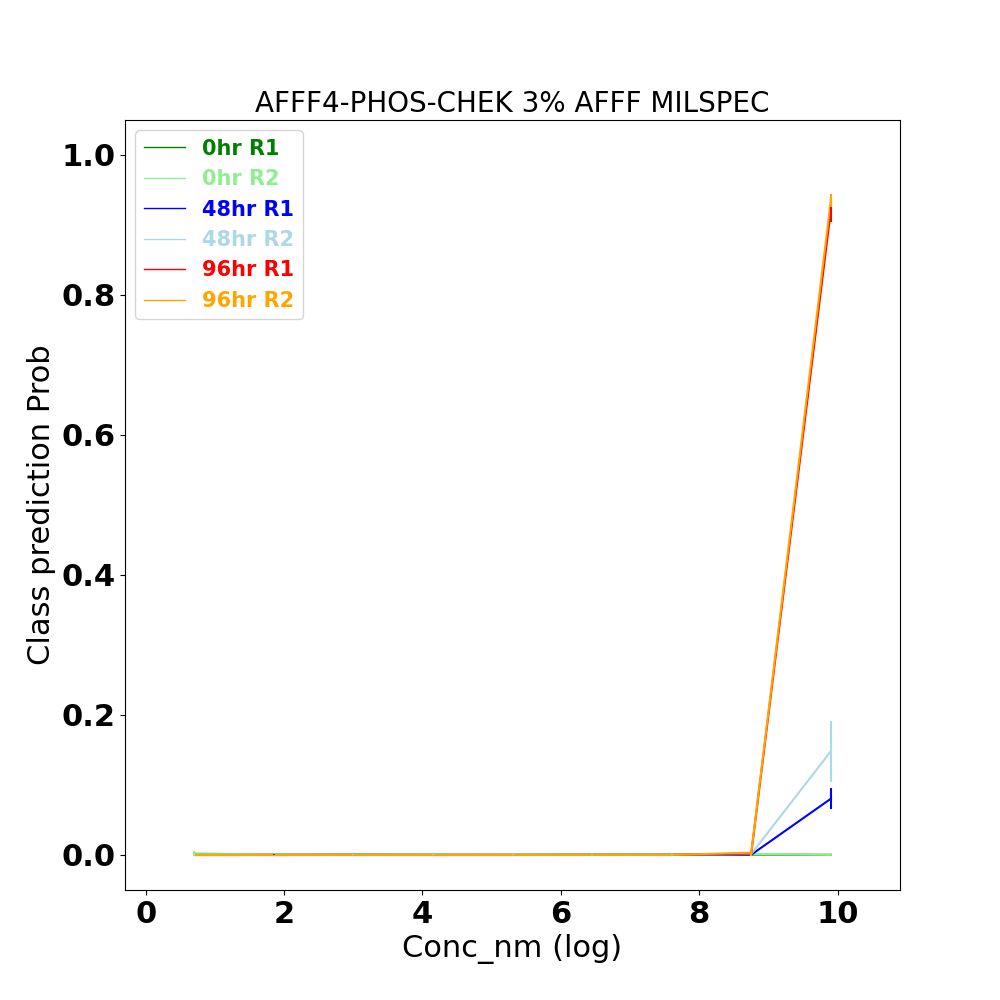

Supplement: Supplementary file 9 — es4c10595_si_009.zip [file es4c10595_si_009.zip › Cell Morphology Image Analysis-Sciome/Dose_Response_Plots/Dose-reponse-AFFF4-PHOS-CHEK 3% AFFF MILSPEC.png]

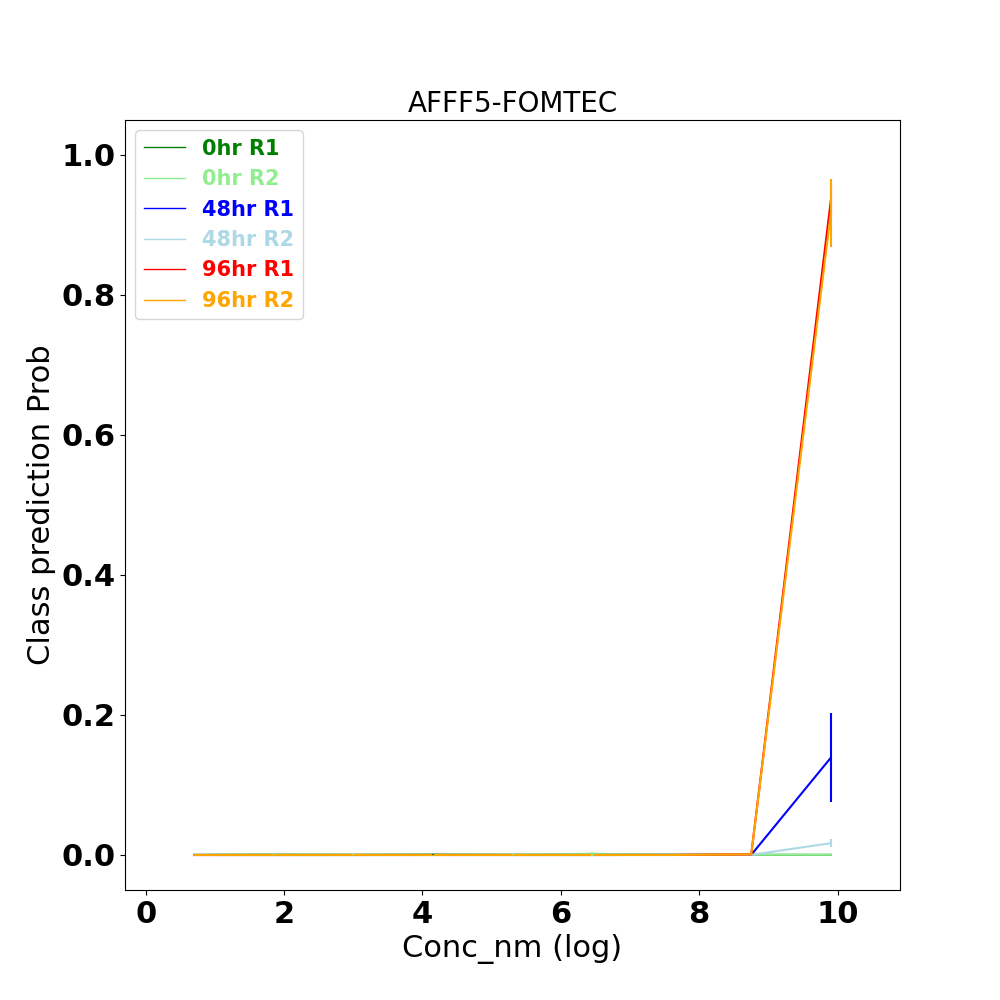

Supplement: Supplementary file 9 — es4c10595_si_009.zip [file es4c10595_si_009.zip › Cell Morphology Image Analysis-Sciome/Dose_Response_Plots/Dose-reponse-AFFF5-FOMTEC.png]

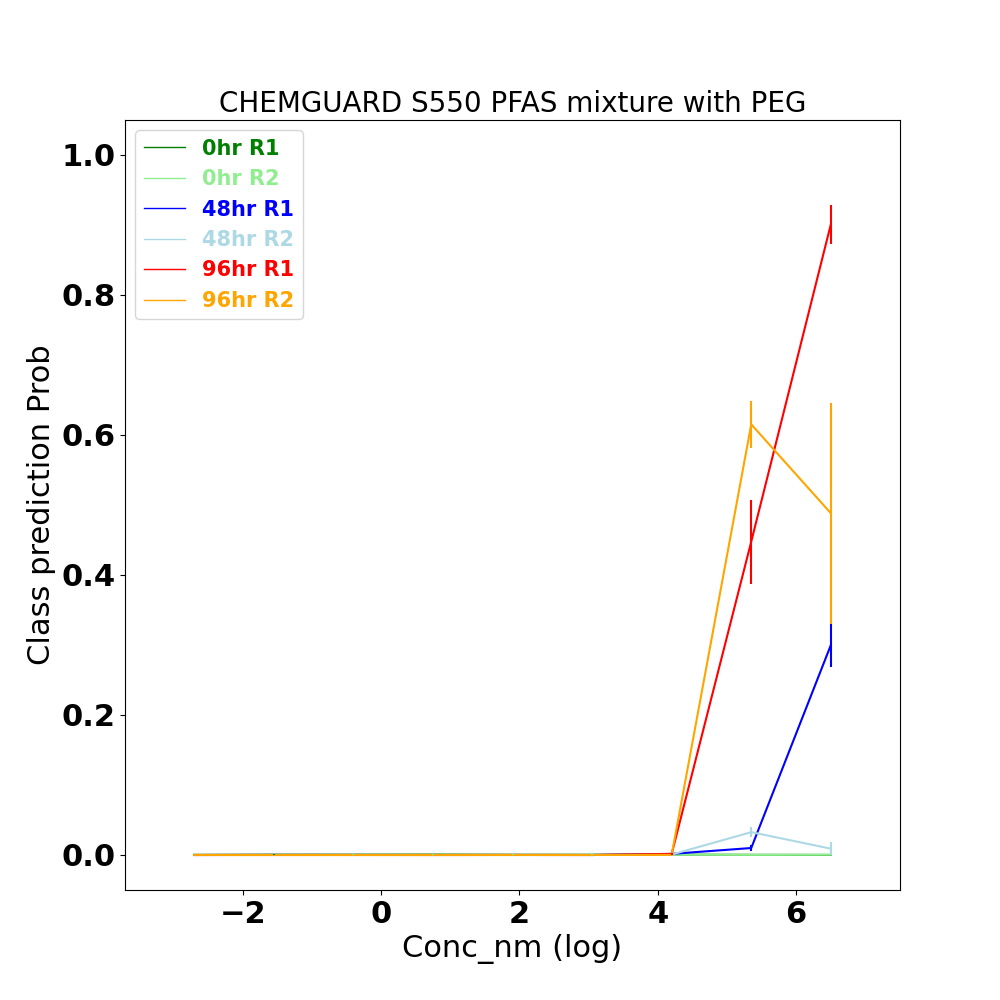

Supplement: Supplementary file 9 — es4c10595_si_009.zip [file es4c10595_si_009.zip › Cell Morphology Image Analysis-Sciome/Dose_Response_Plots/Dose-reponse-CHEMGUARD S550 PFAS mixture with PEG.png]

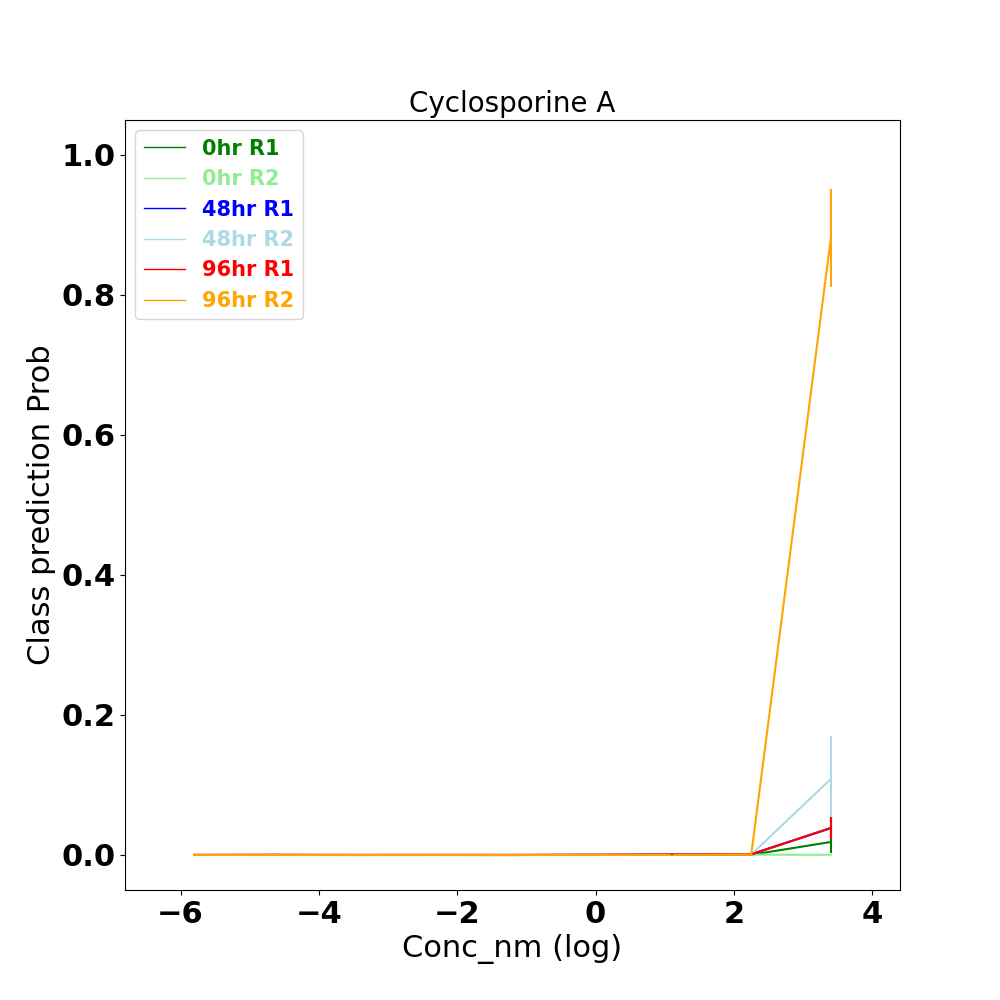

Supplement: Supplementary file 9 — es4c10595_si_009.zip [file es4c10595_si_009.zip › Cell Morphology Image Analysis-Sciome/Dose_Response_Plots/Dose-reponse-Cyclosporine A.png]

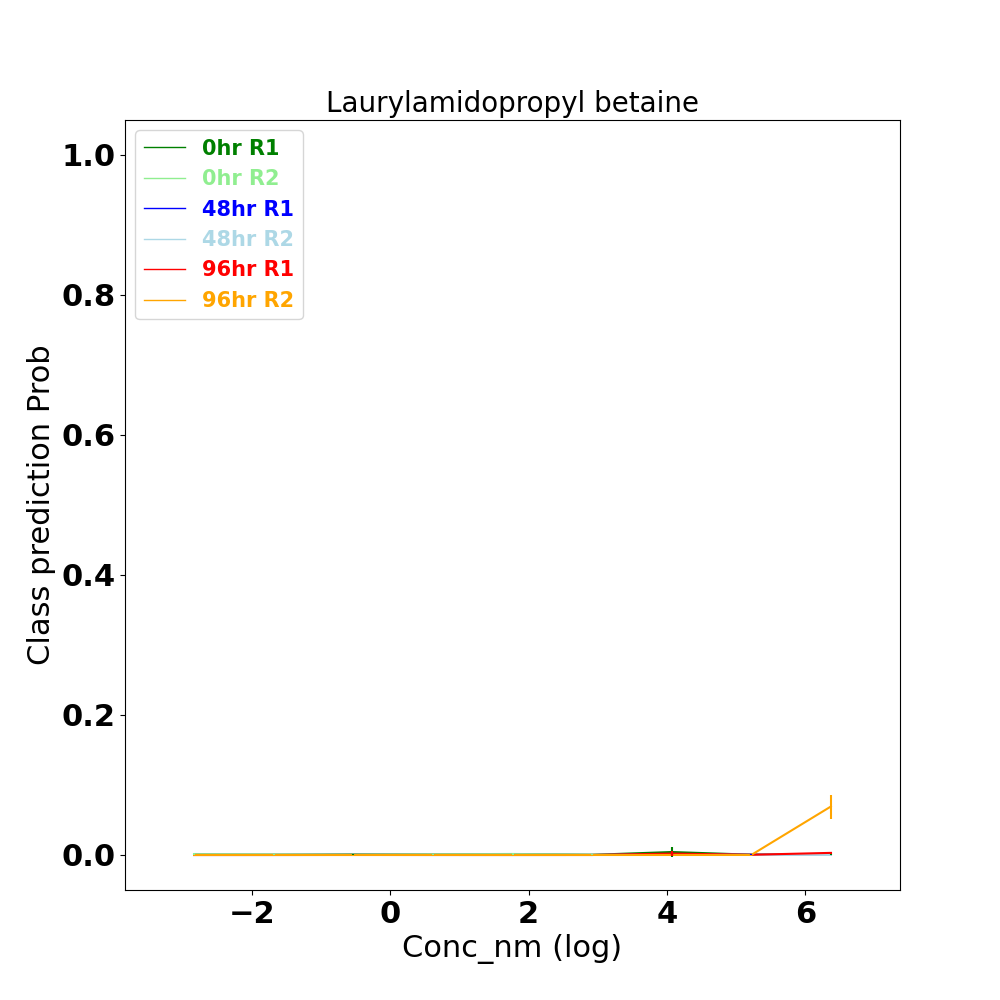

Supplement: Supplementary file 9 — es4c10595_si_009.zip [file es4c10595_si_009.zip › Cell Morphology Image Analysis-Sciome/Dose_Response_Plots/Dose-reponse-Laurylamidopropyl betaine.png]

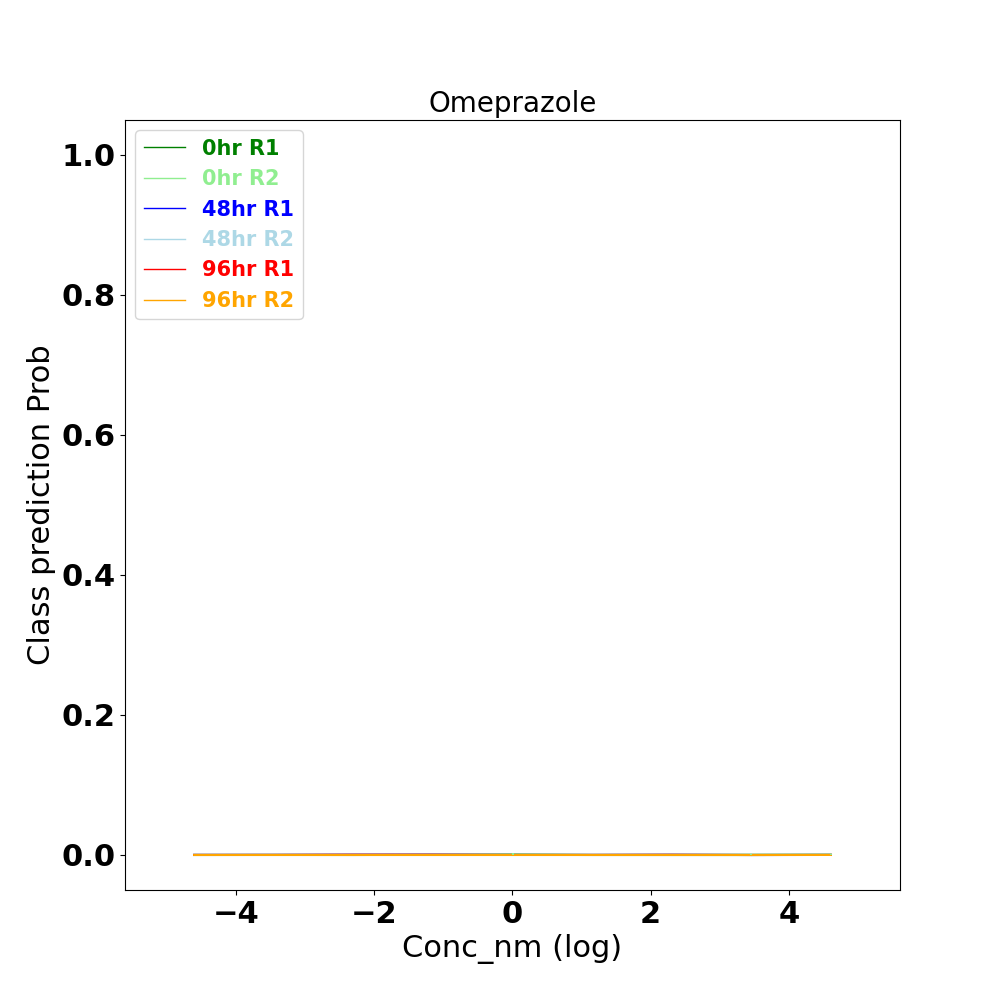

Supplement: Supplementary file 9 — es4c10595_si_009.zip [file es4c10595_si_009.zip › Cell Morphology Image Analysis-Sciome/Dose_Response_Plots/Dose-reponse-Omeprazole.png]

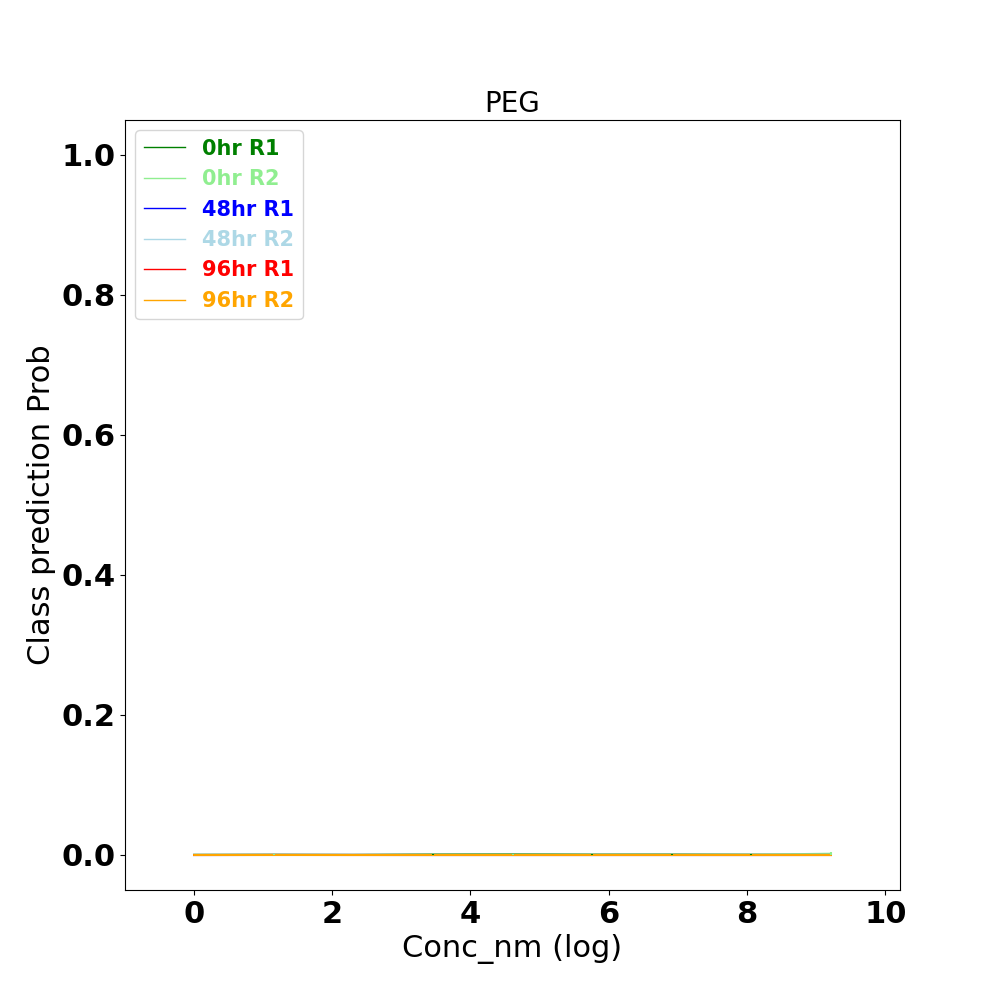

Supplement: Supplementary file 9 — es4c10595_si_009.zip [file es4c10595_si_009.zip › Cell Morphology Image Analysis-Sciome/Dose_Response_Plots/Dose-reponse-PEG.png]

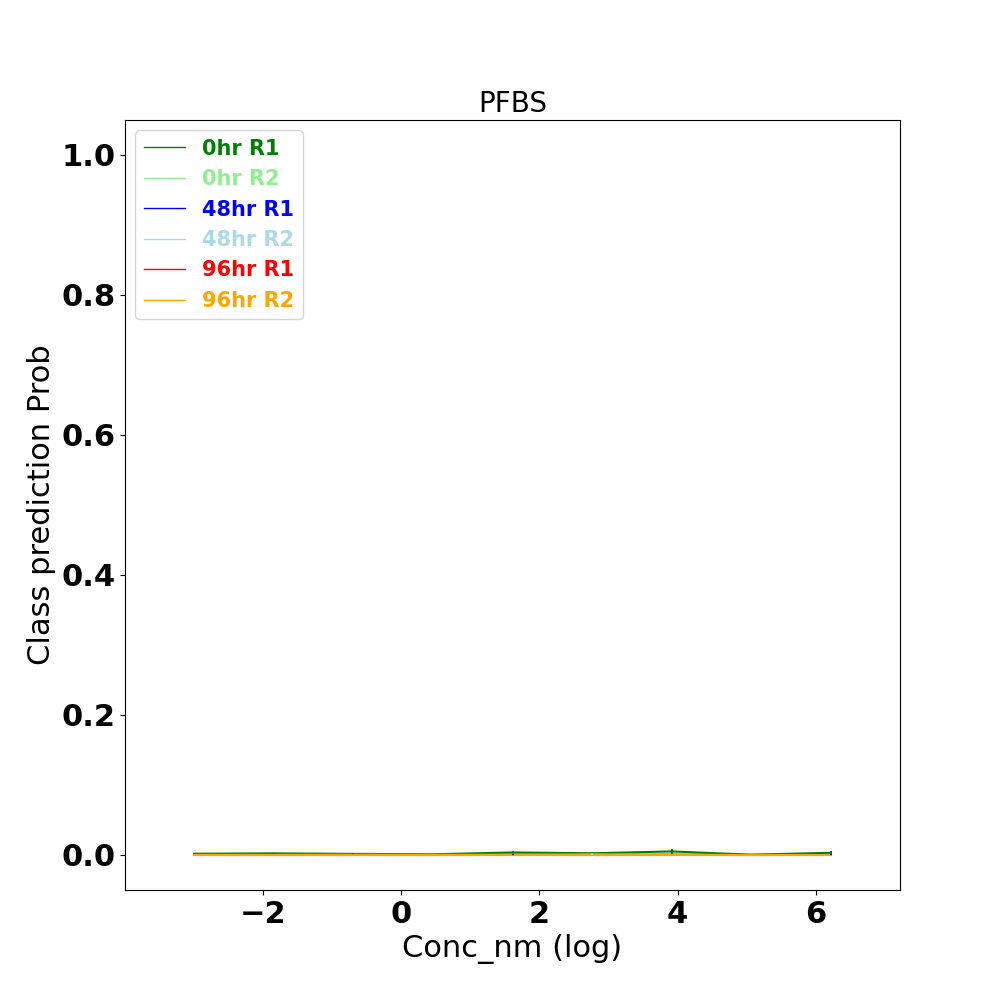

Supplement: Supplementary file 9 — es4c10595_si_009.zip [file es4c10595_si_009.zip › Cell Morphology Image Analysis-Sciome/Dose_Response_Plots/Dose-reponse-PFBS.png]

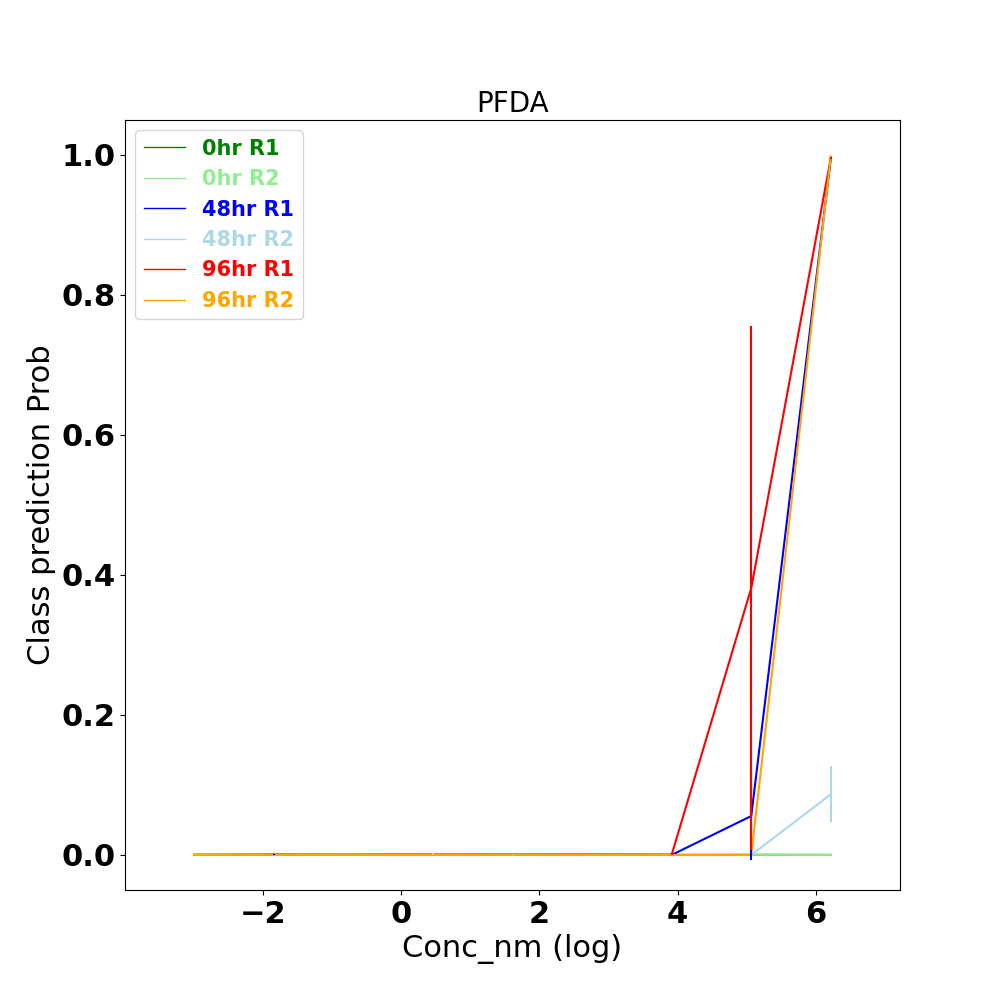

Supplement: Supplementary file 9 — es4c10595_si_009.zip [file es4c10595_si_009.zip › Cell Morphology Image Analysis-Sciome/Dose_Response_Plots/Dose-reponse-PFDA.png]

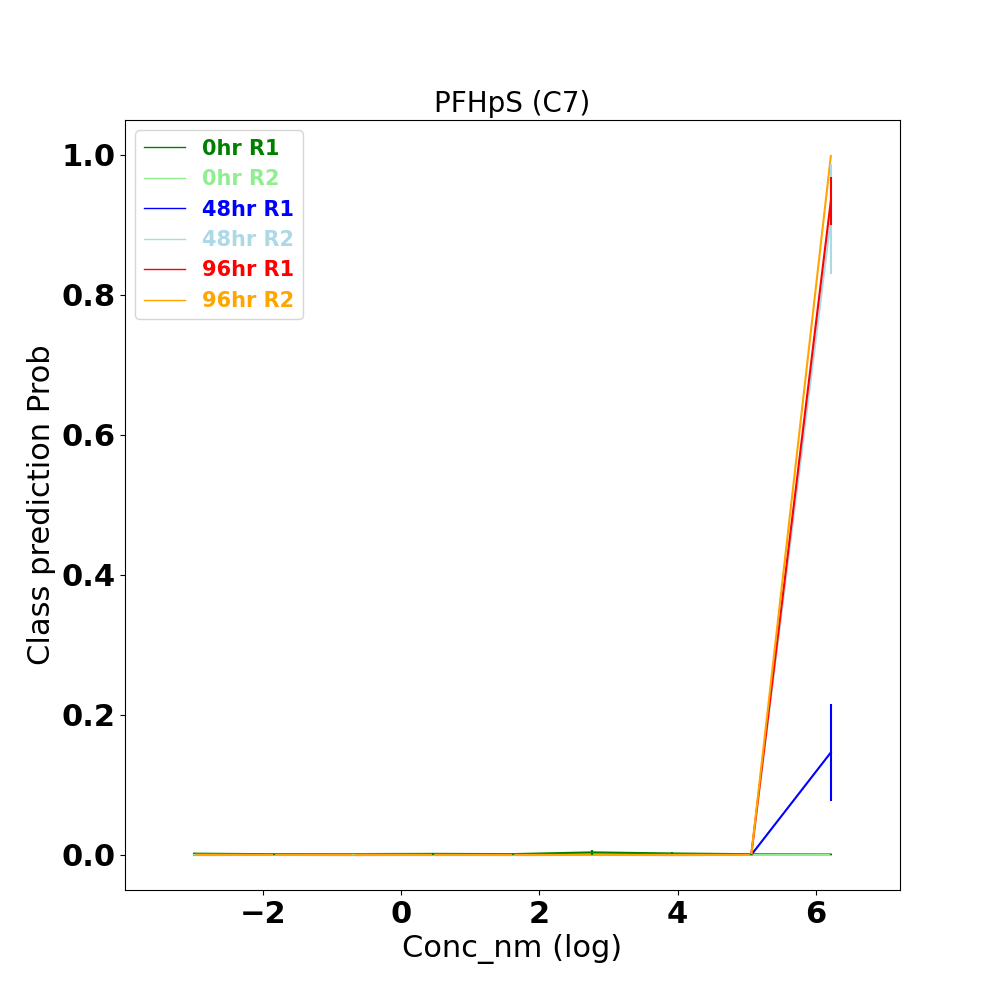

Supplement: Supplementary file 9 — es4c10595_si_009.zip [file es4c10595_si_009.zip › Cell Morphology Image Analysis-Sciome/Dose_Response_Plots/Dose-reponse-PFHpS (C7).png]

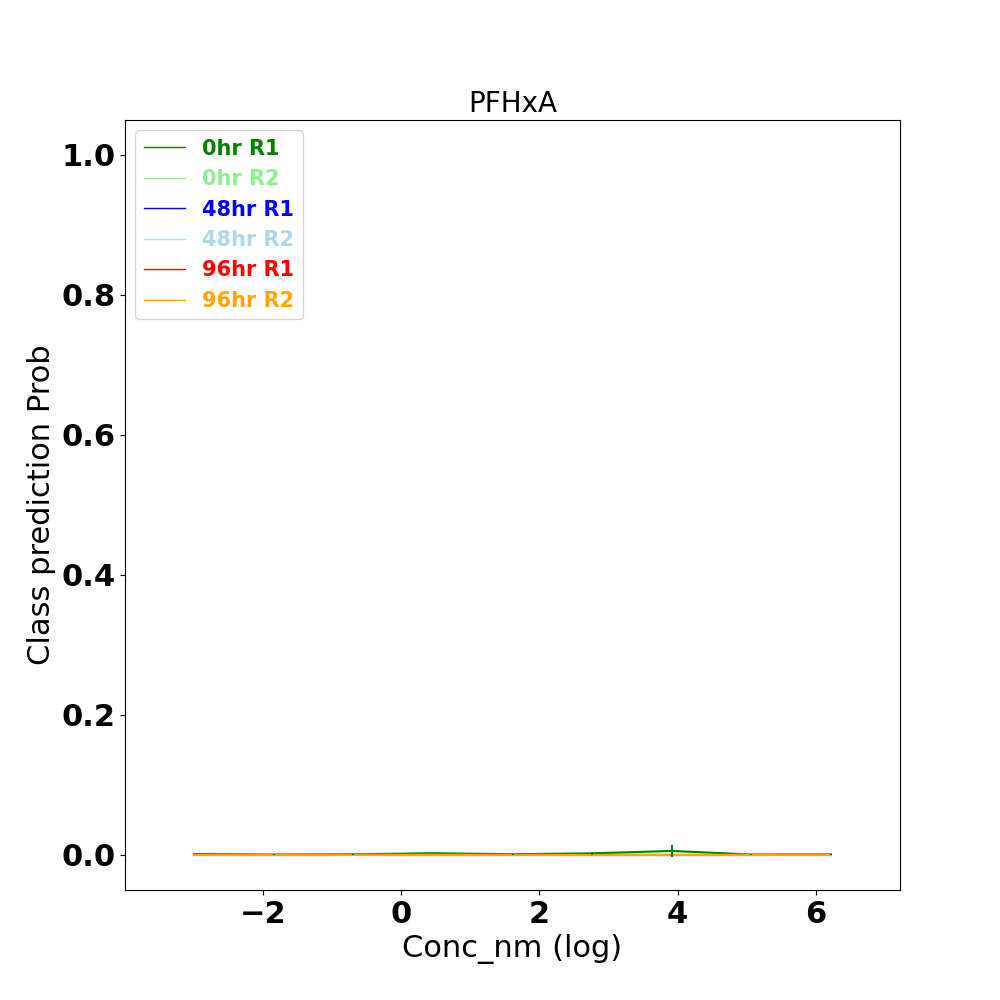

Supplement: Supplementary file 9 — es4c10595_si_009.zip [file es4c10595_si_009.zip › Cell Morphology Image Analysis-Sciome/Dose_Response_Plots/Dose-reponse-PFHxA.png]

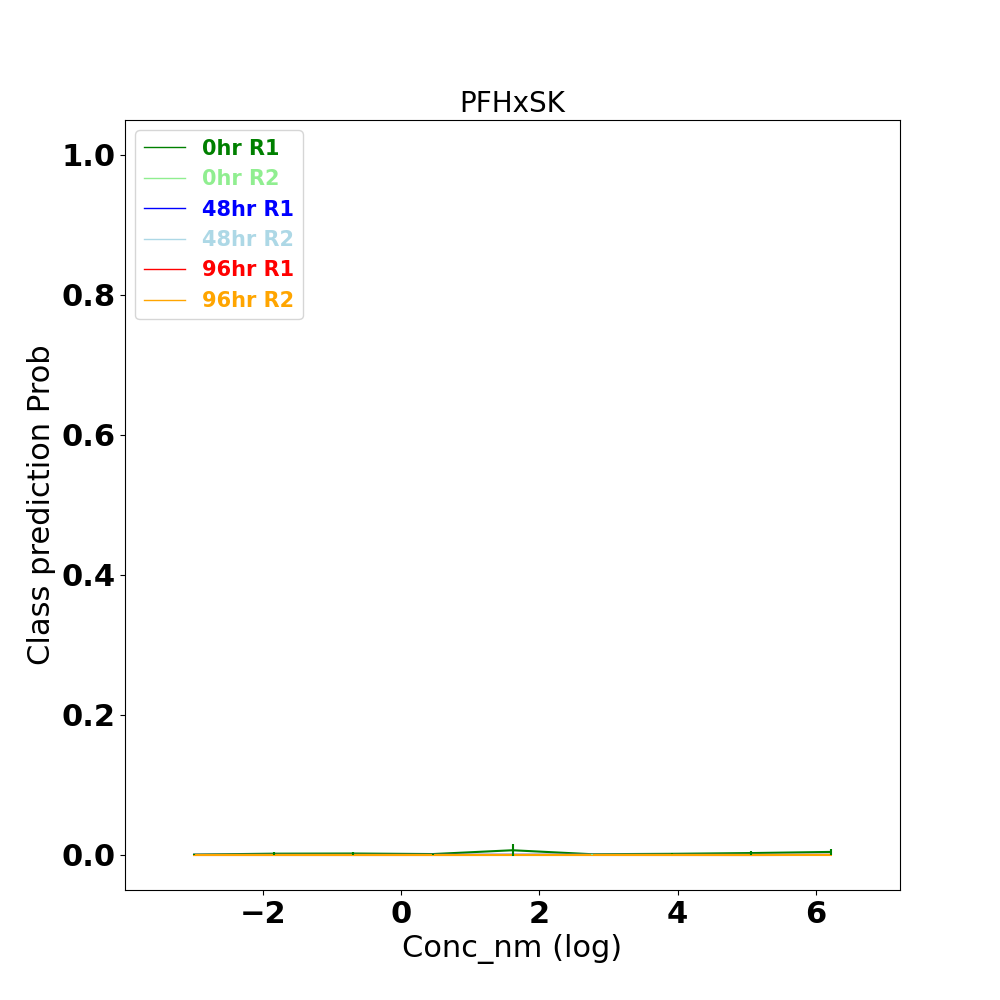

Supplement: Supplementary file 9 — es4c10595_si_009.zip [file es4c10595_si_009.zip › Cell Morphology Image Analysis-Sciome/Dose_Response_Plots/Dose-reponse-PFHxSK.png]

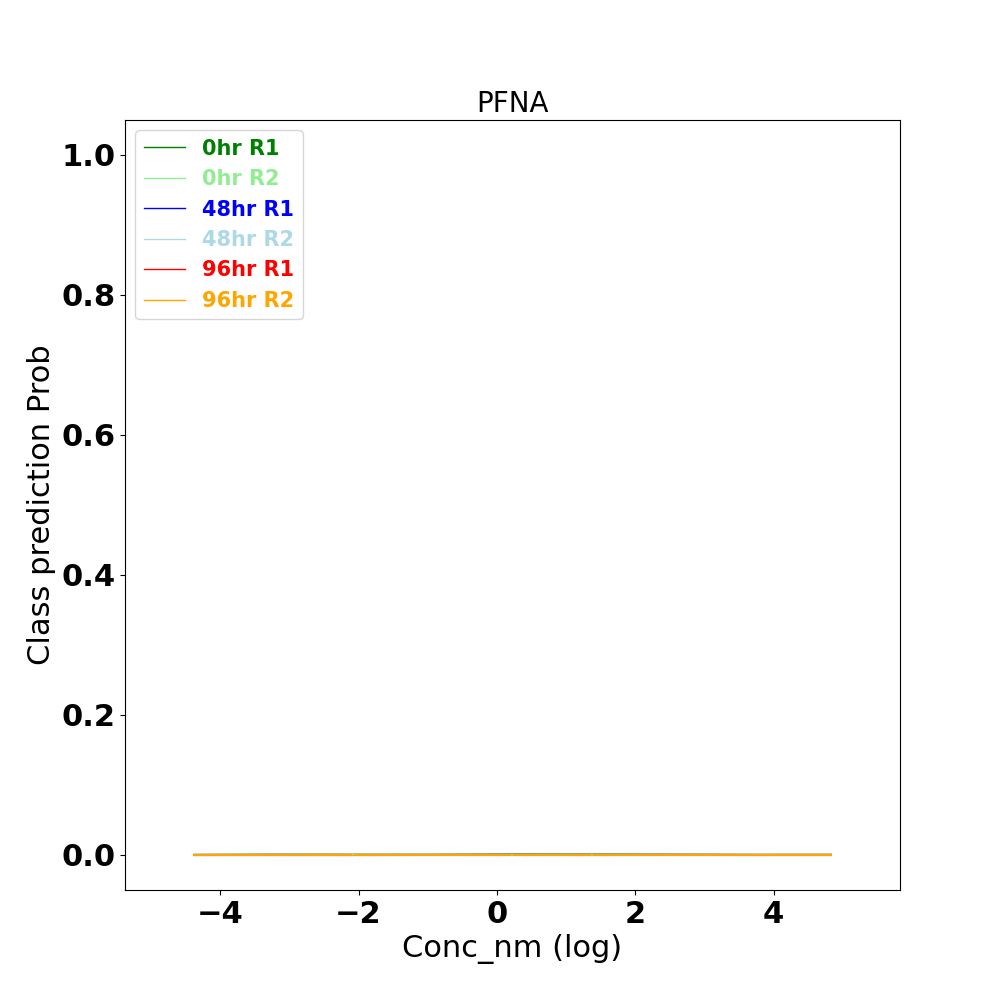

Supplement: Supplementary file 9 — es4c10595_si_009.zip [file es4c10595_si_009.zip › Cell Morphology Image Analysis-Sciome/Dose_Response_Plots/Dose-reponse-PFNA.png]

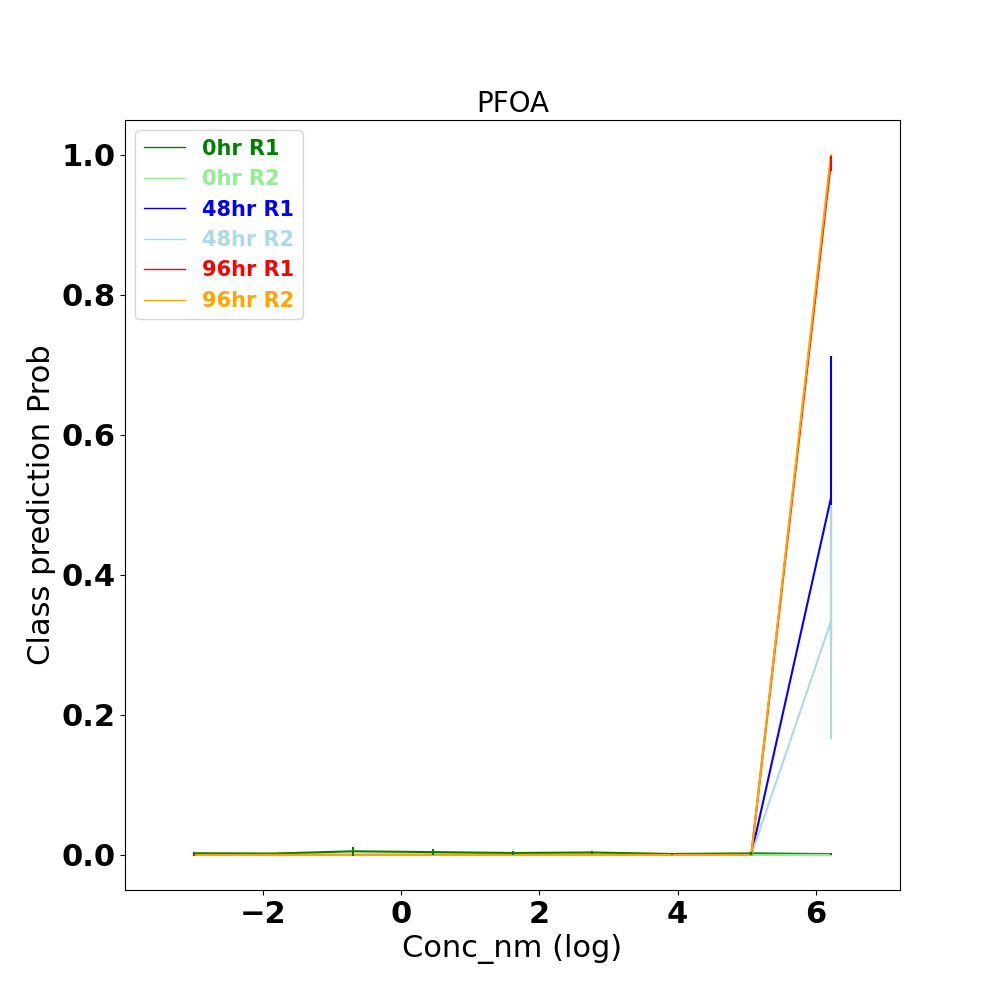

Supplement: Supplementary file 9 — es4c10595_si_009.zip [file es4c10595_si_009.zip › Cell Morphology Image Analysis-Sciome/Dose_Response_Plots/Dose-reponse-PFOA.png]

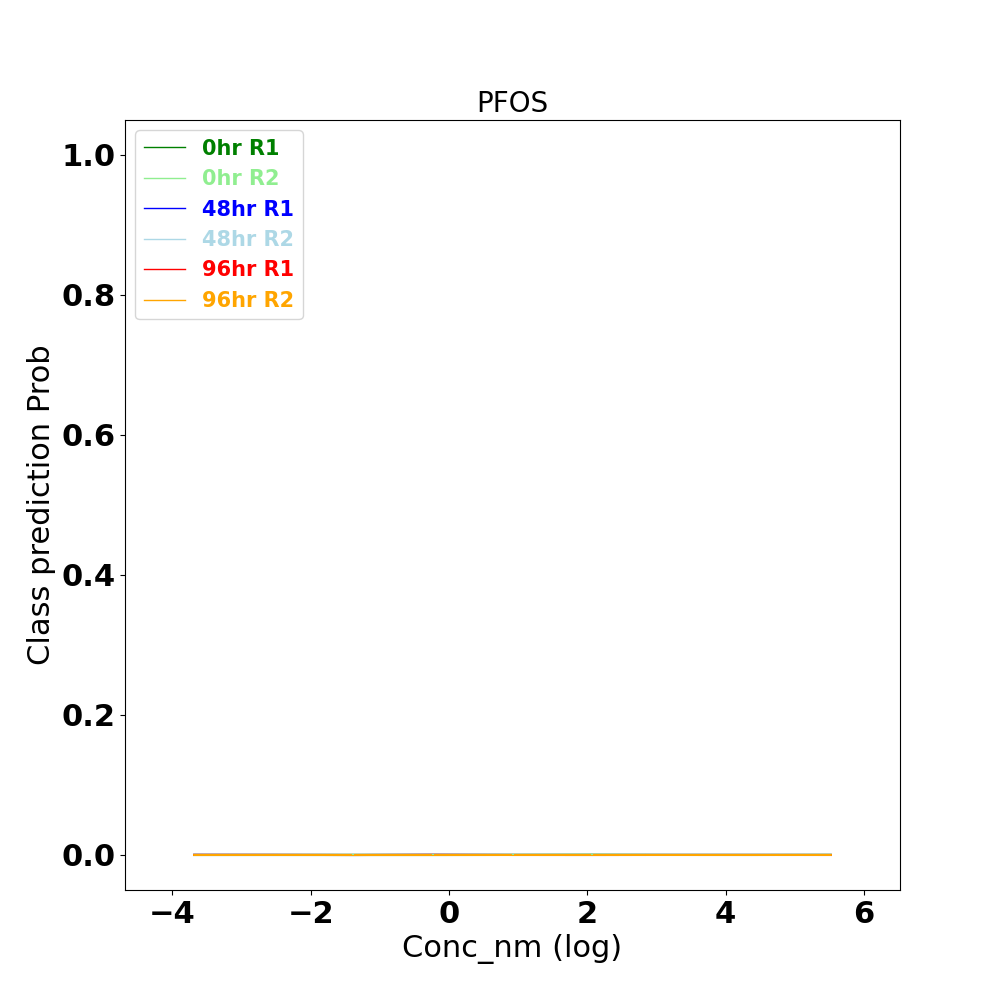

Supplement: Supplementary file 9 — es4c10595_si_009.zip [file es4c10595_si_009.zip › Cell Morphology Image Analysis-Sciome/Dose_Response_Plots/Dose-reponse-PFOS.png]

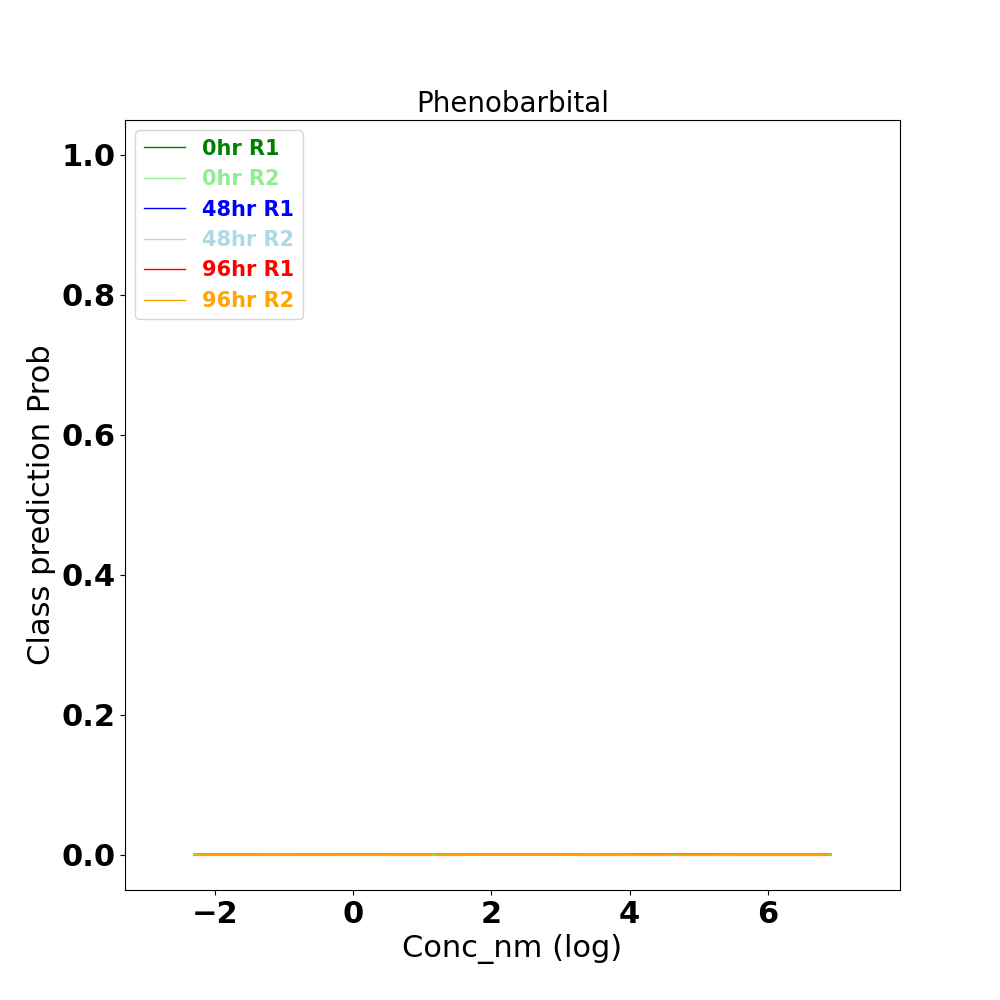

Supplement: Supplementary file 9 — es4c10595_si_009.zip [file es4c10595_si_009.zip › Cell Morphology Image Analysis-Sciome/Dose_Response_Plots/Dose-reponse-Phenobarbital.png]

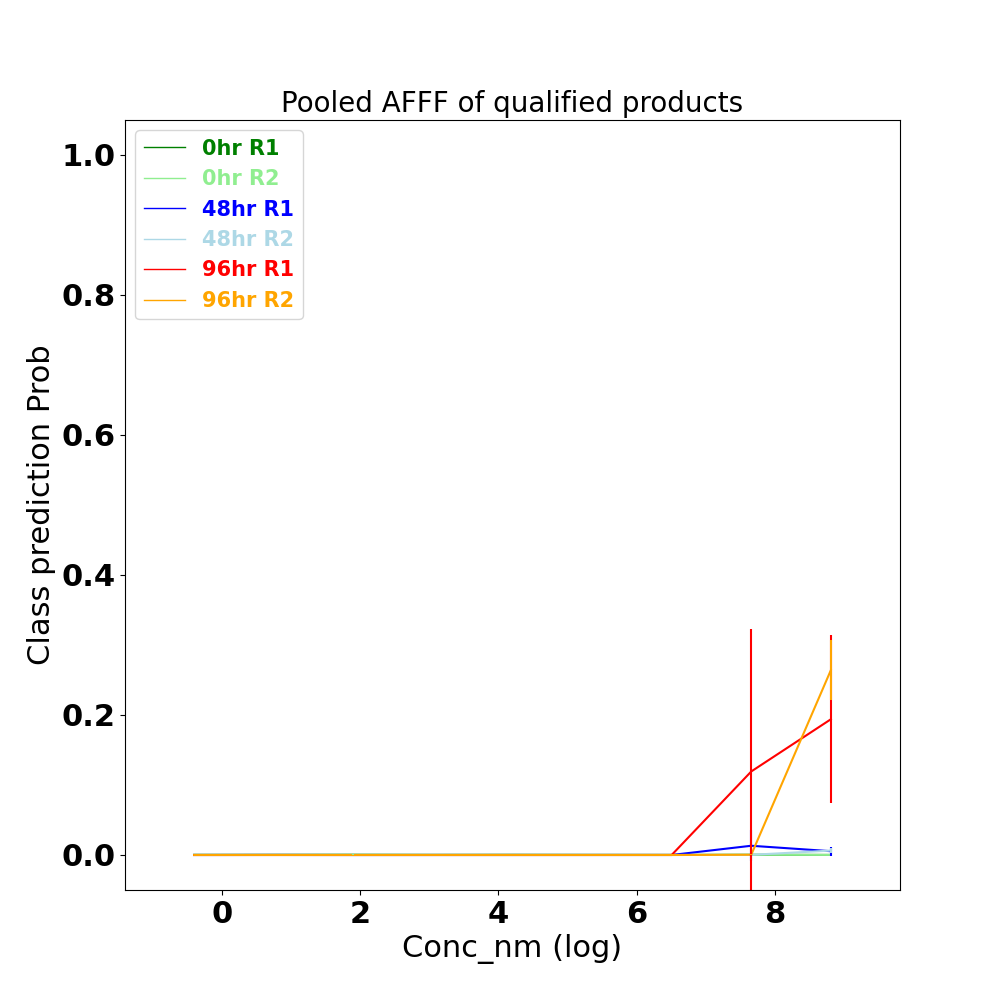

Supplement: Supplementary file 9 — es4c10595_si_009.zip [file es4c10595_si_009.zip › Cell Morphology Image Analysis-Sciome/Dose_Response_Plots/Dose-reponse-Pooled AFFF of qualified products.png]

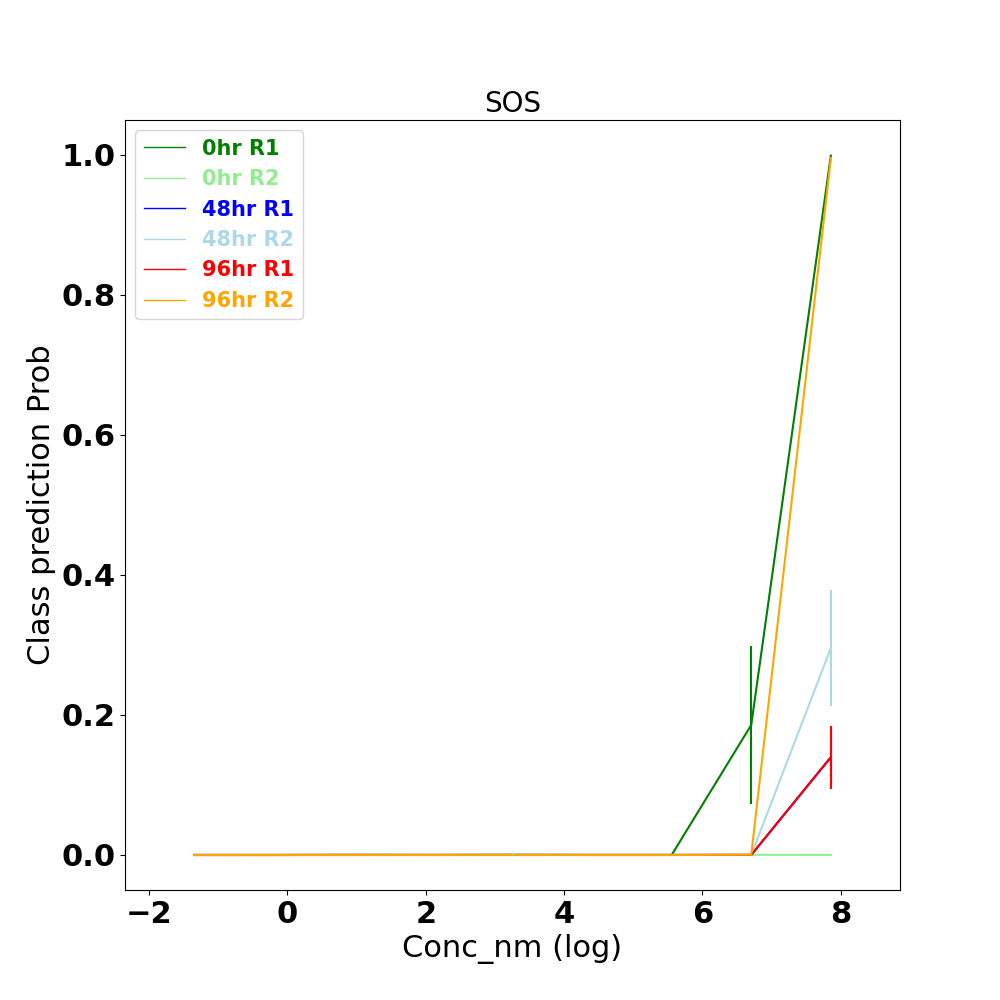

Supplement: Supplementary file 9 — es4c10595_si_009.zip [file es4c10595_si_009.zip › Cell Morphology Image Analysis-Sciome/Dose_Response_Plots/Dose-reponse-SOS.png]

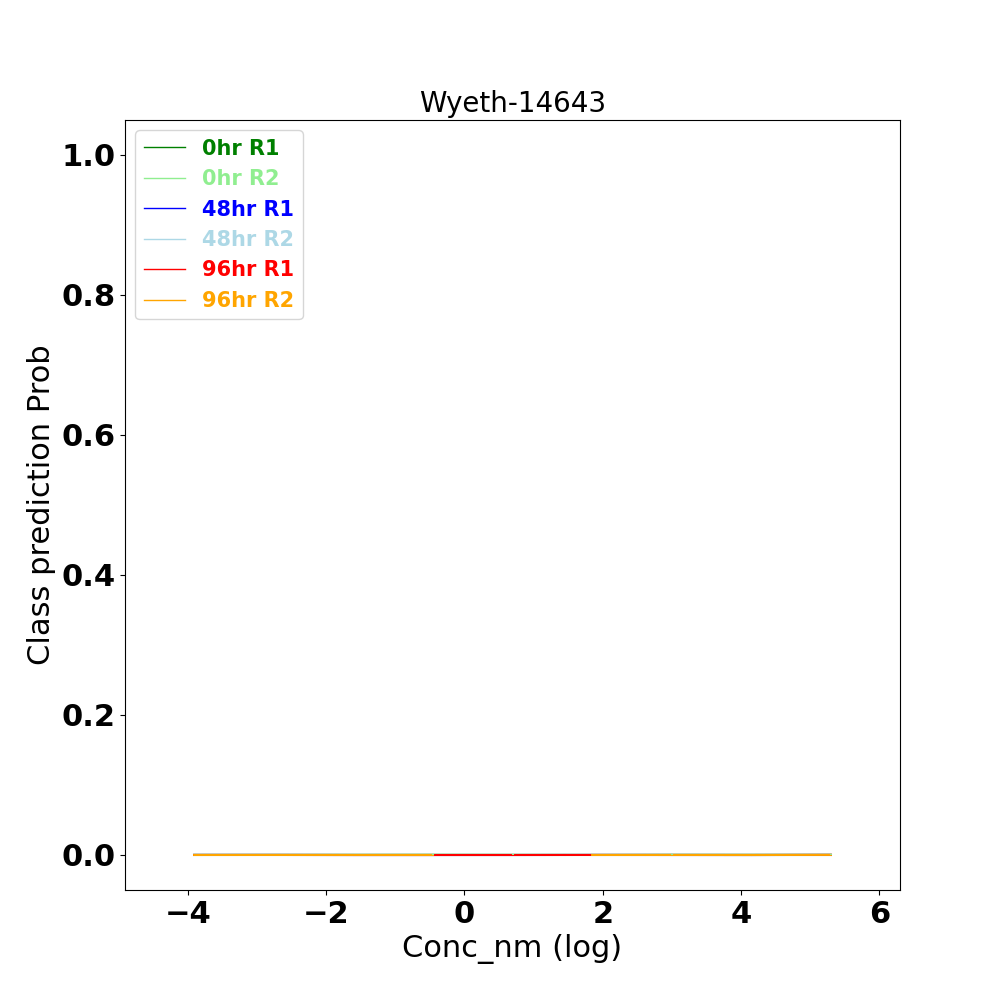

Supplement: Supplementary file 9 — es4c10595_si_009.zip [file es4c10595_si_009.zip › Cell Morphology Image Analysis-Sciome/Dose_Response_Plots/Dose-reponse-Wyeth-14643.png]

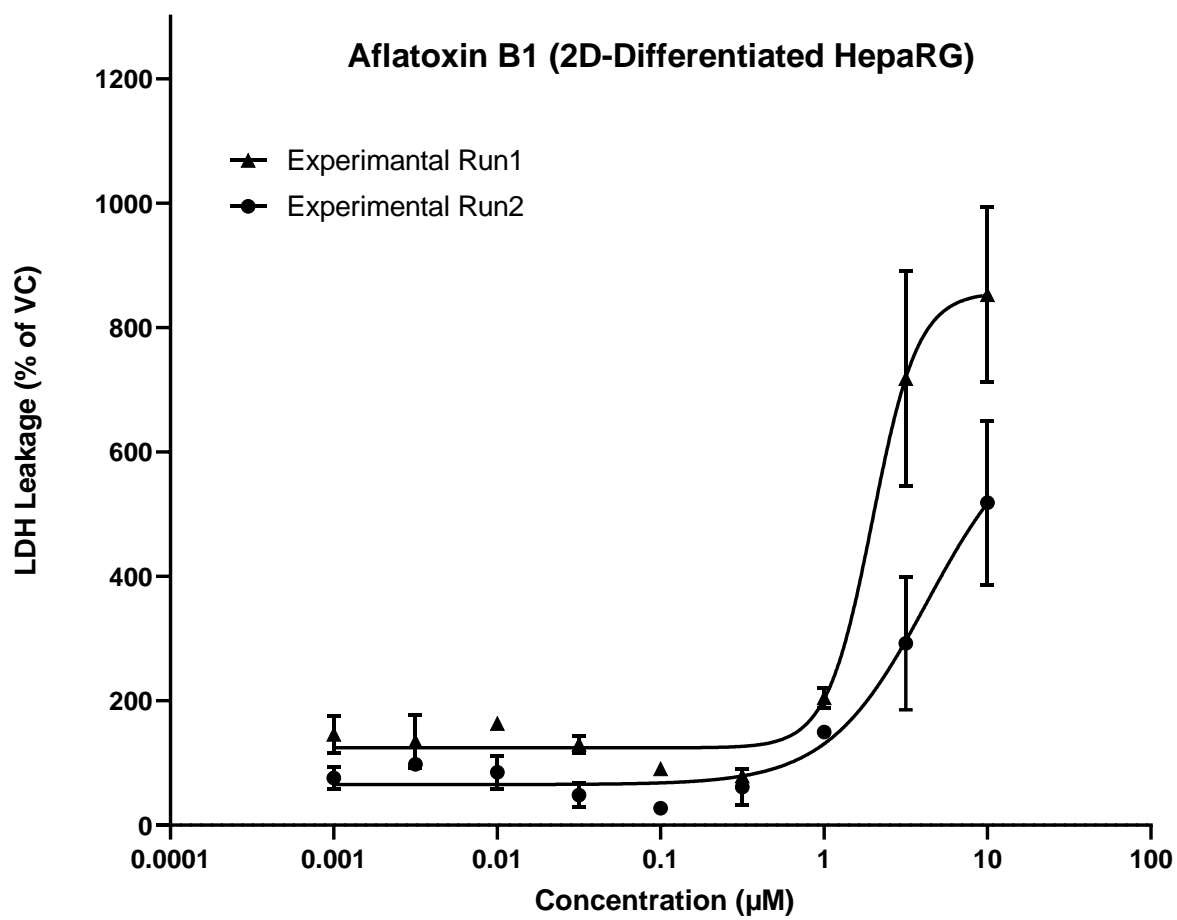

Supplement: Supplementary file 10 — es4c10595_si_010.zip [file es4c10595_si_010.zip › Analyzed LDH Leakage from HTT Runs/LDH Leakage AFB1 Supplemental Figure.pdf]
