# Supplementary material for: Unraveling Human Hepatocellular Responses to PFAS and Aqueous Film-Forming Foams (AFFFs) for Molecular Hazard Prioritization and In Vivo Translation
Source: Environ Sci Technol. 2025 Feb 2;59(5):2423–35. doi: 10.1021/acs.est.4c10595 (PMC11823446; doi:10.1021/acs.est.4c10595)
Supplement: Supplementary file 10 — es4c10595_si_010.zip [file es4c10595_si_010.zip › Analyzed LDH Leakage from HTT Runs/FInal LDH BMD Graphs - Run 2.pptx]

## Slide 1
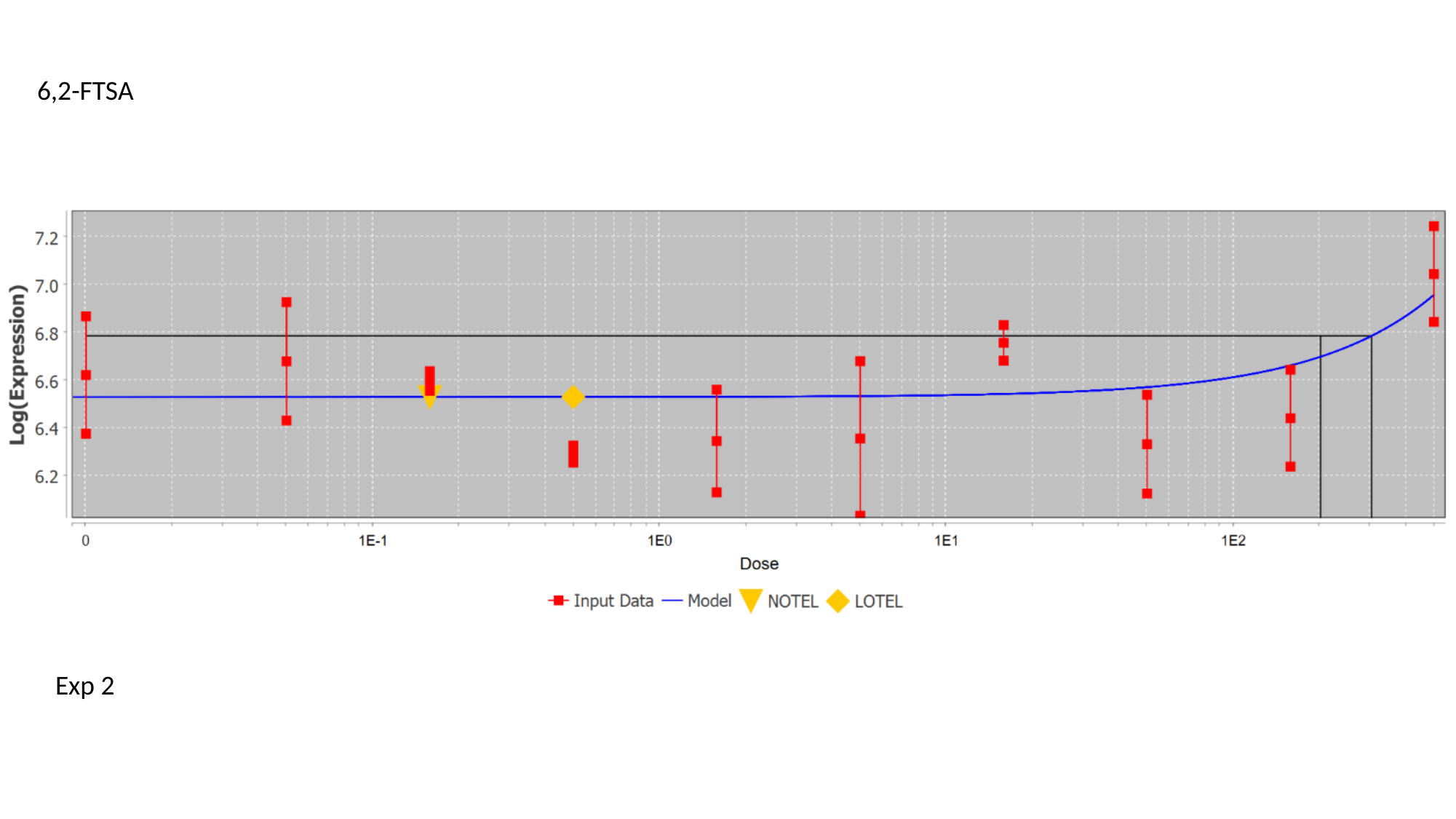

6,2-FTSA
Exp 2

## Slide 2
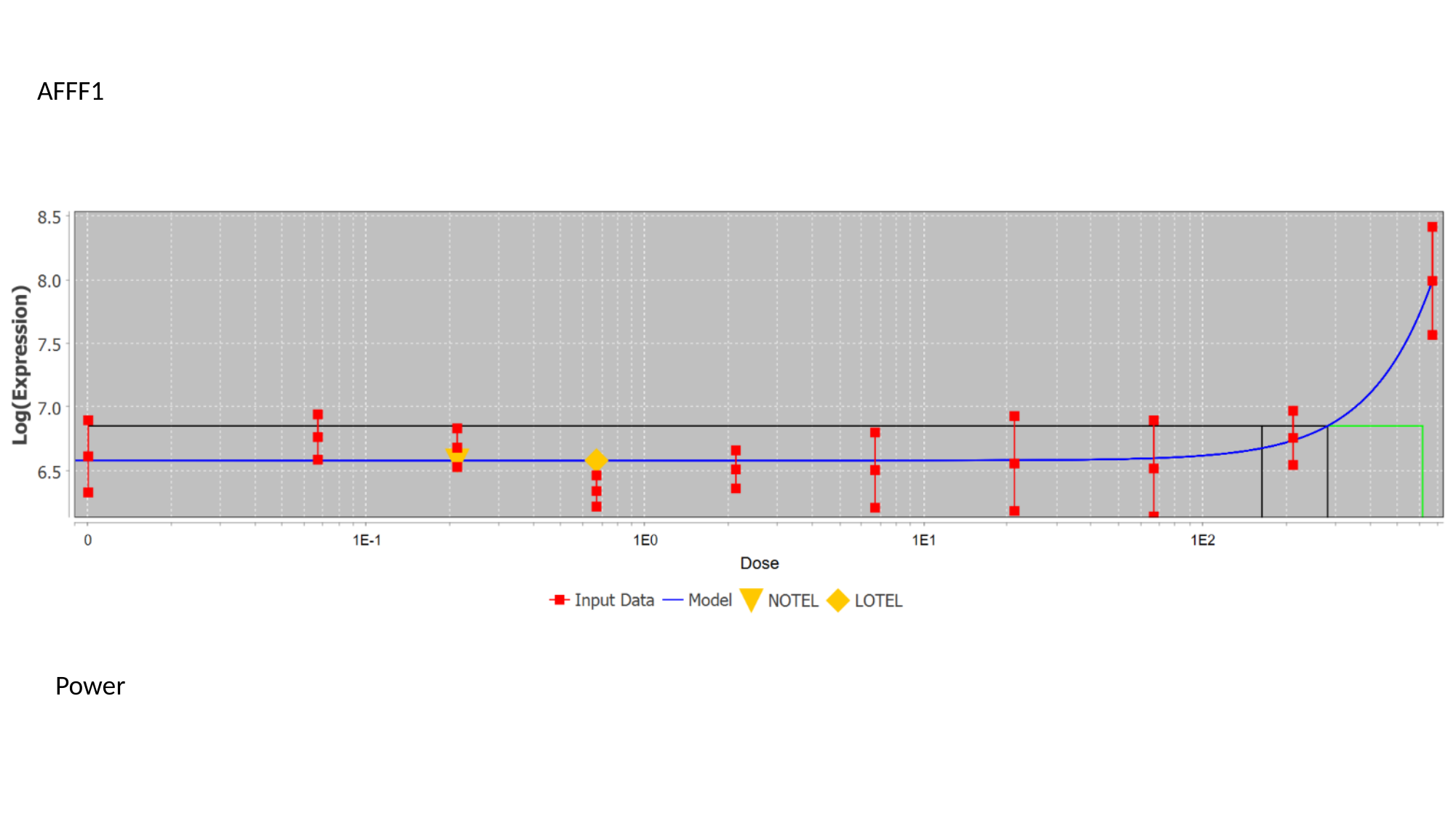

AFFF1
Power

## Slide 3
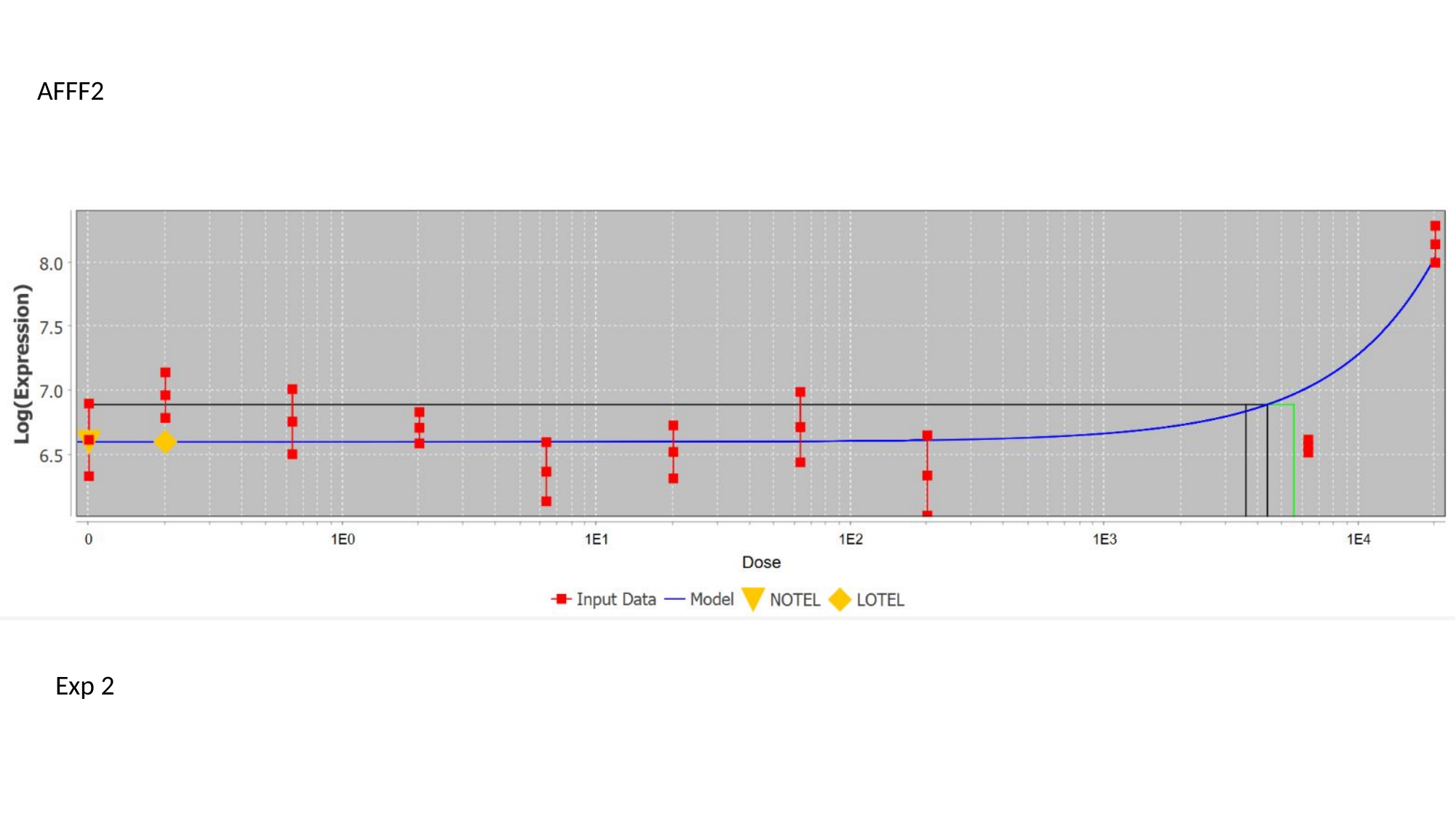

AFFF2
Exp 2

## Slide 4
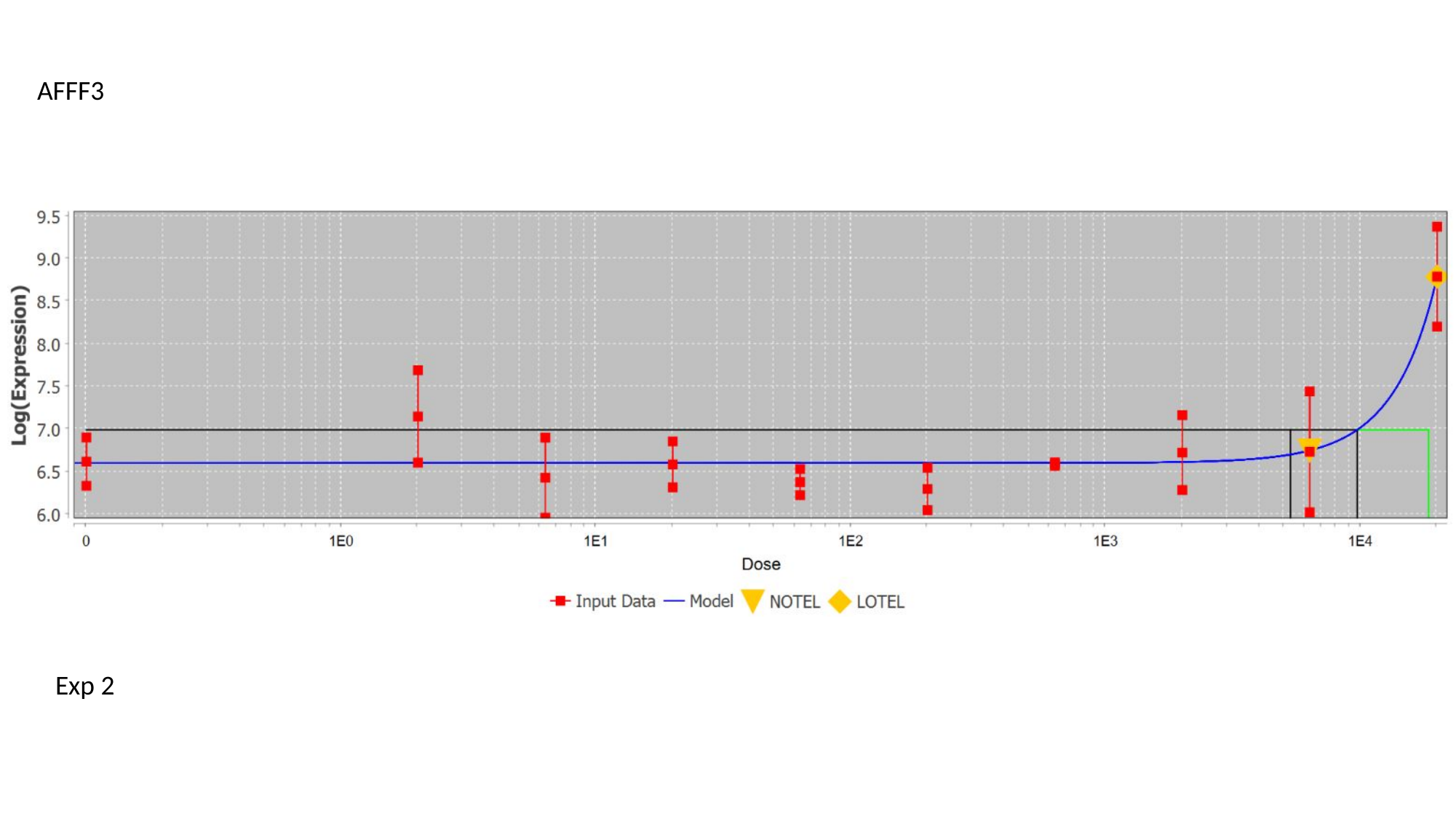

AFFF3
Exp 2

## Slide 5
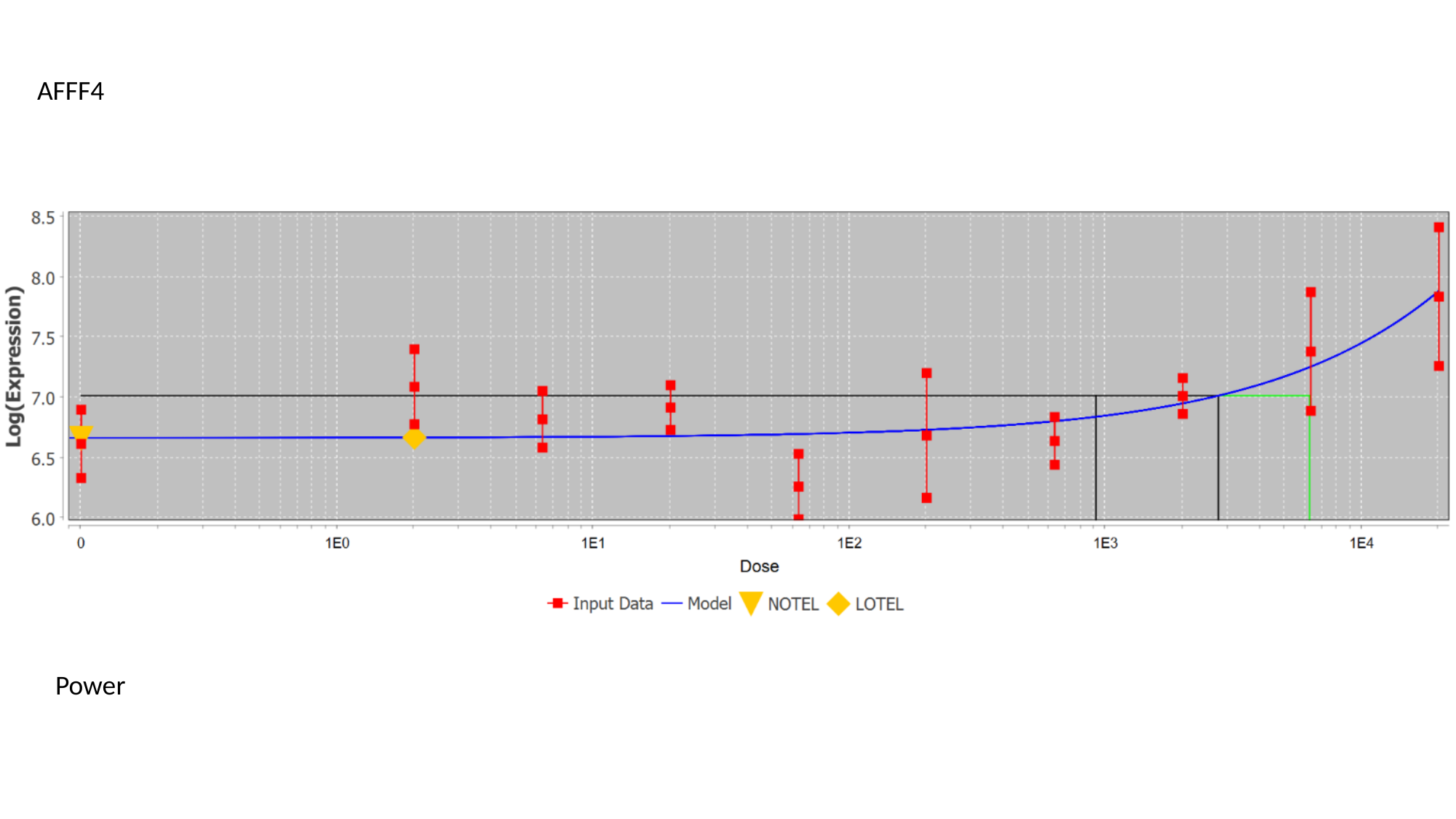

AFFF4
Power

## Slide 6
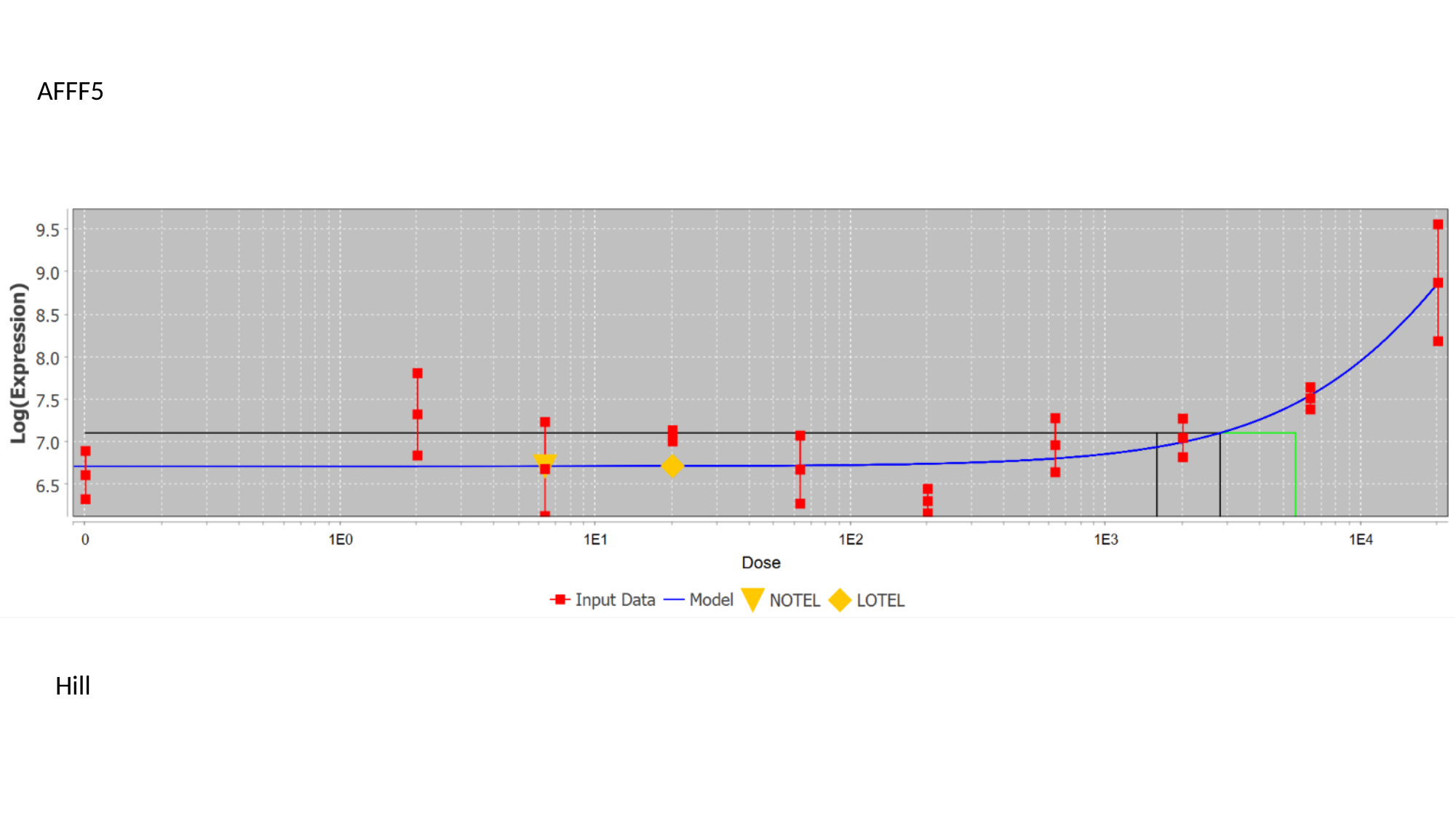

AFFF5
Hill

## Slide 7
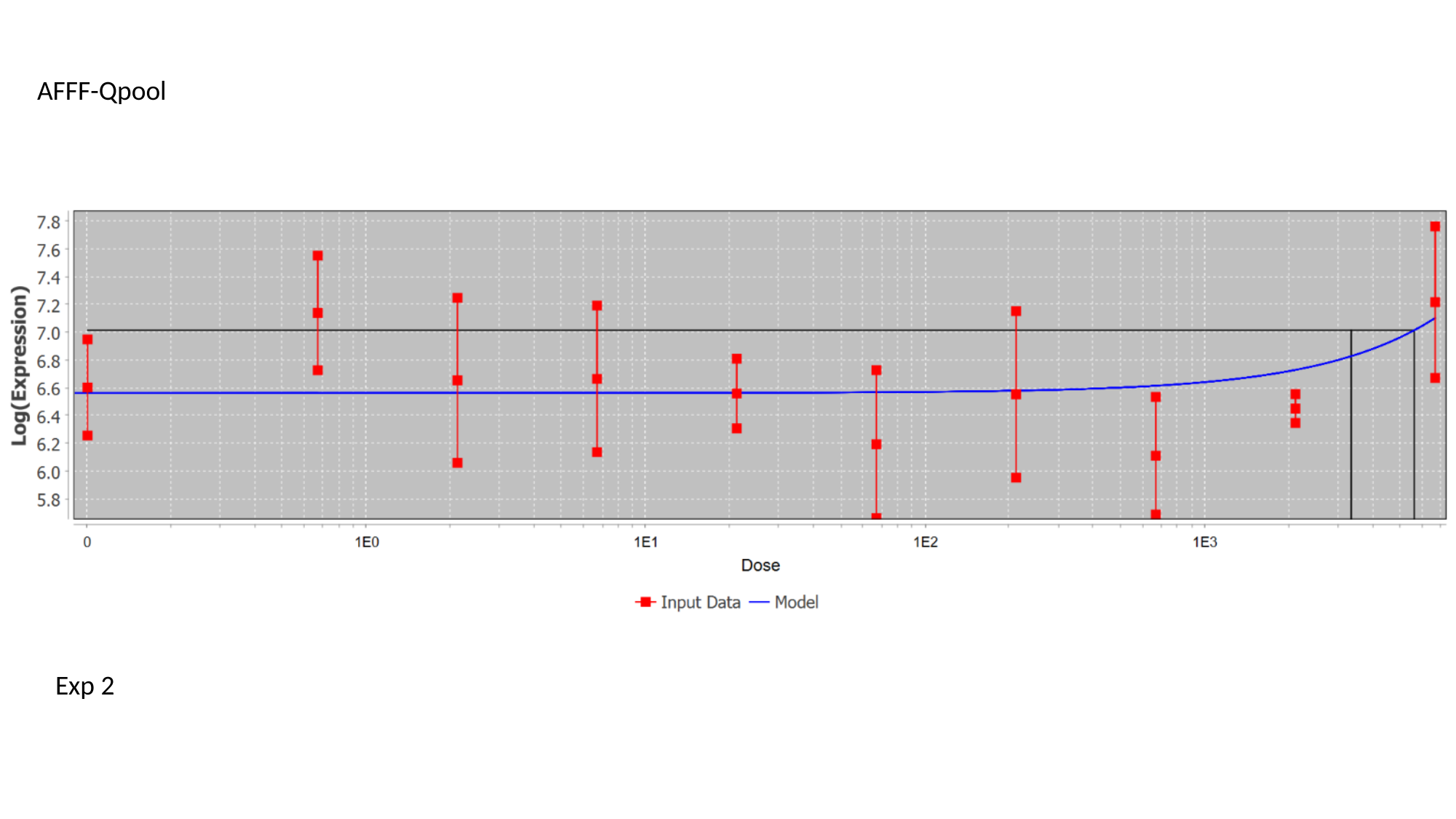

AFFF-Qpool
Exp 2

## Slide 8
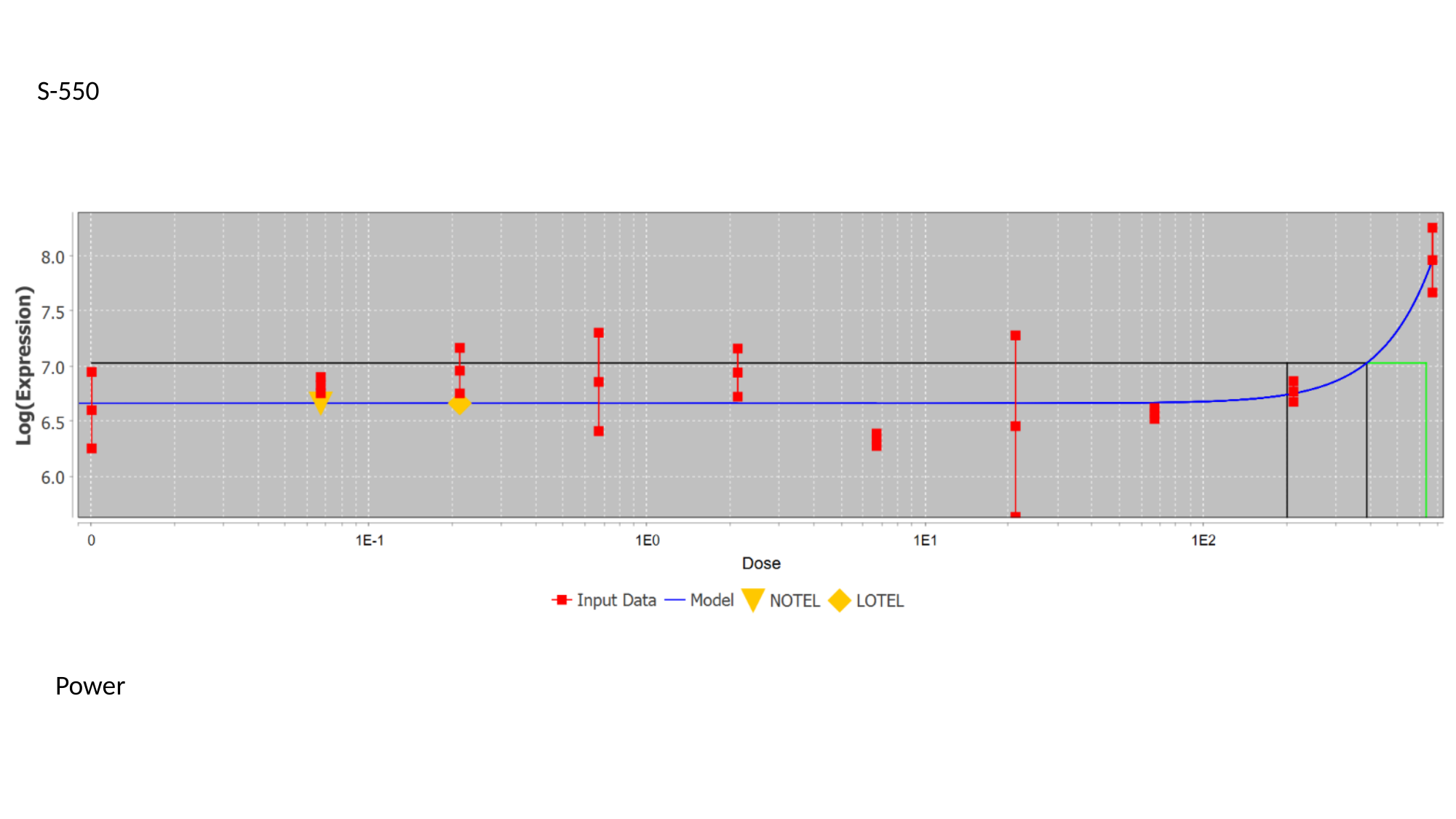

S-550
Power

## Slide 9
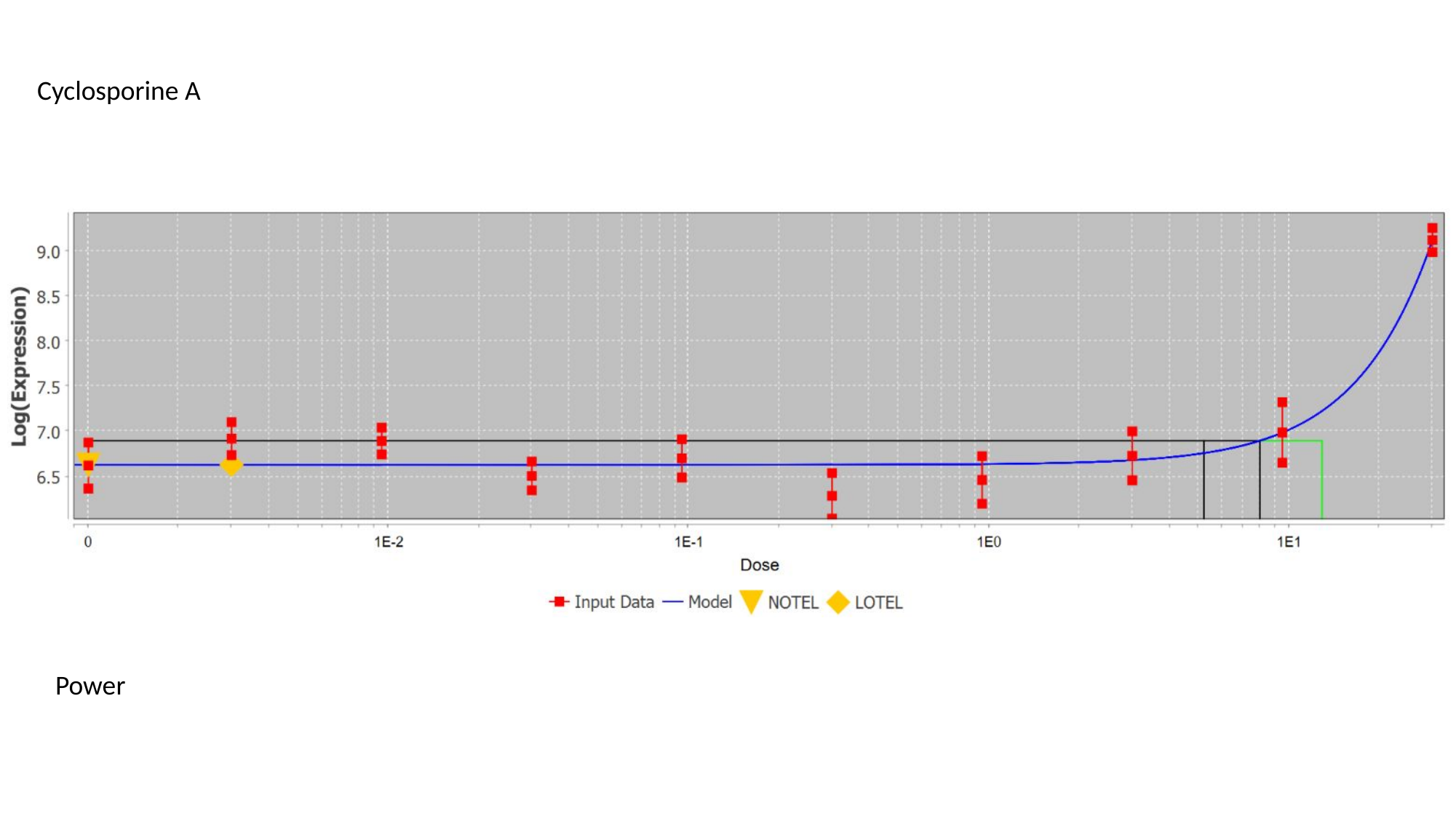

Cyclosporine A
Power

## Slide 10
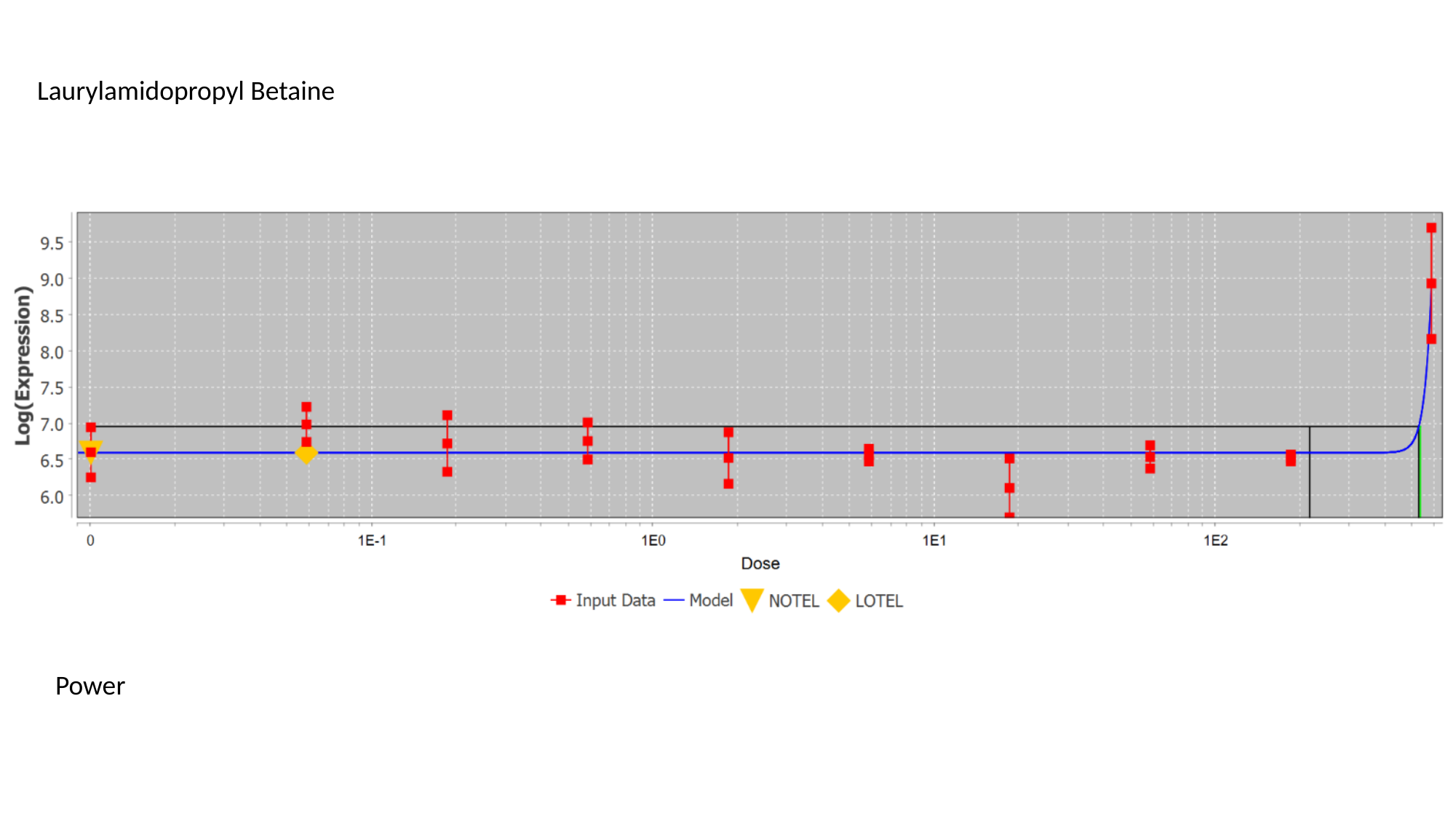

Laurylamidopropyl Betaine
Power

## Slide 11
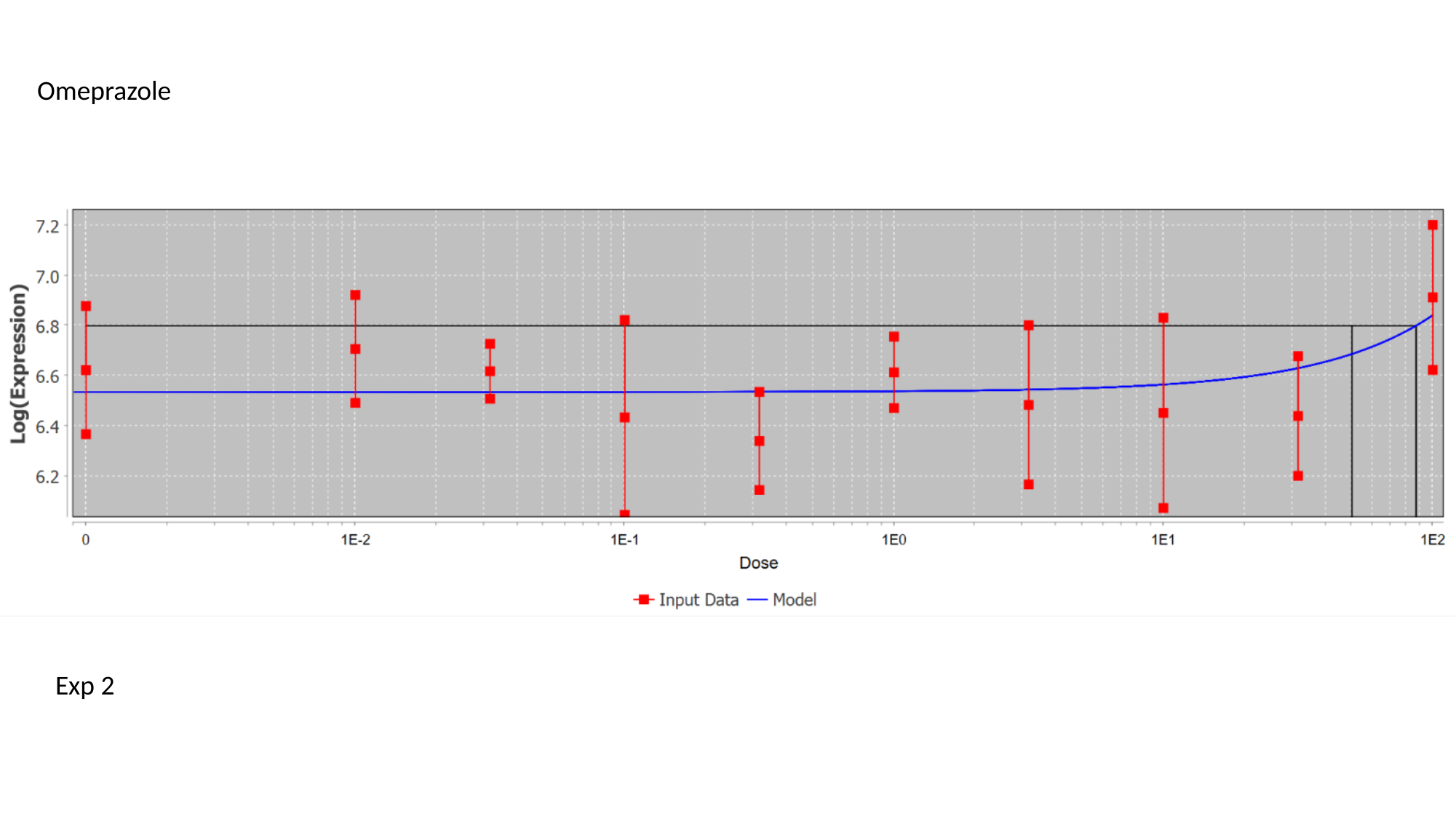

Omeprazole
Exp 2

## Slide 12
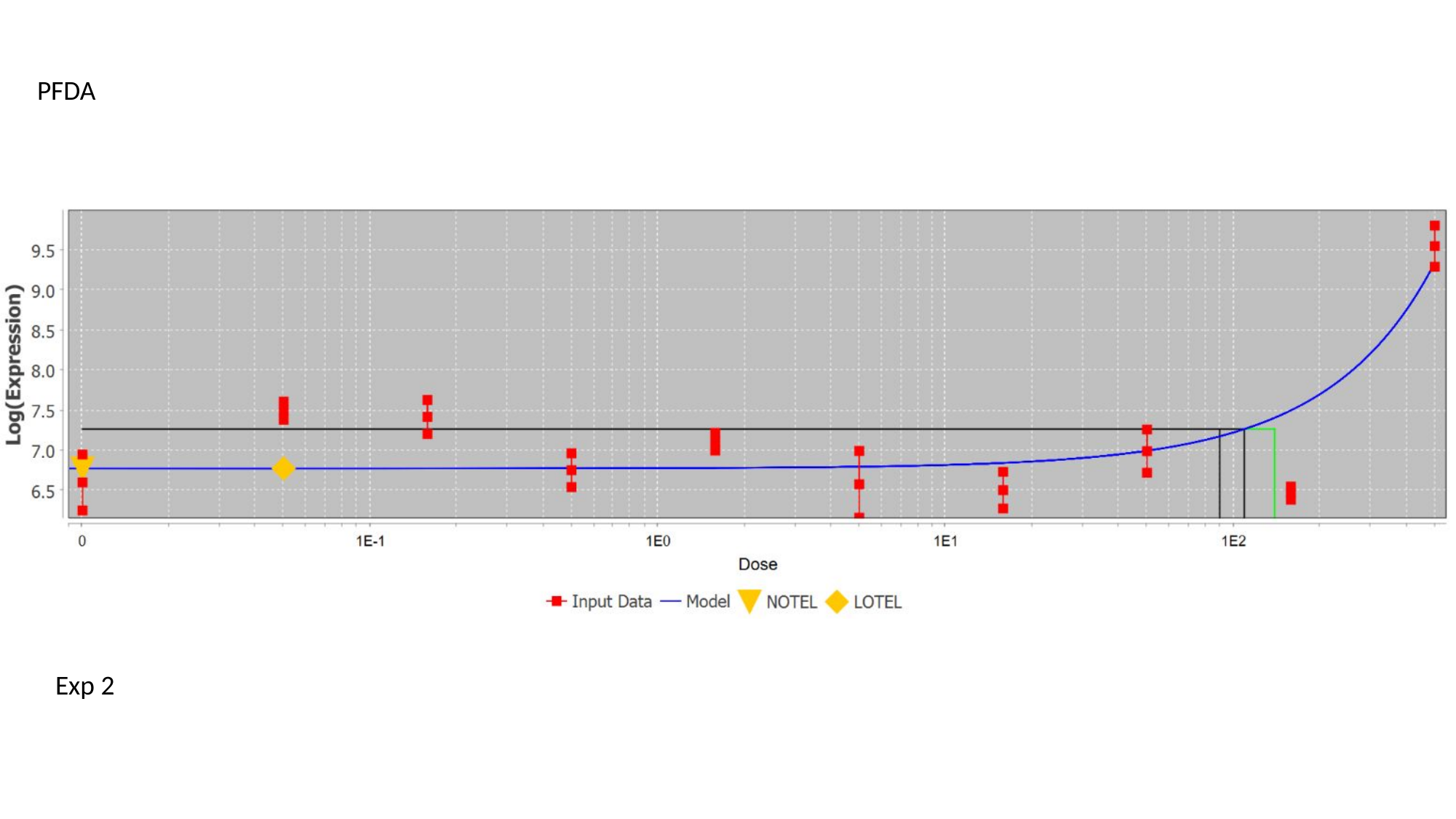

PFDA
Exp 2

## Slide 13
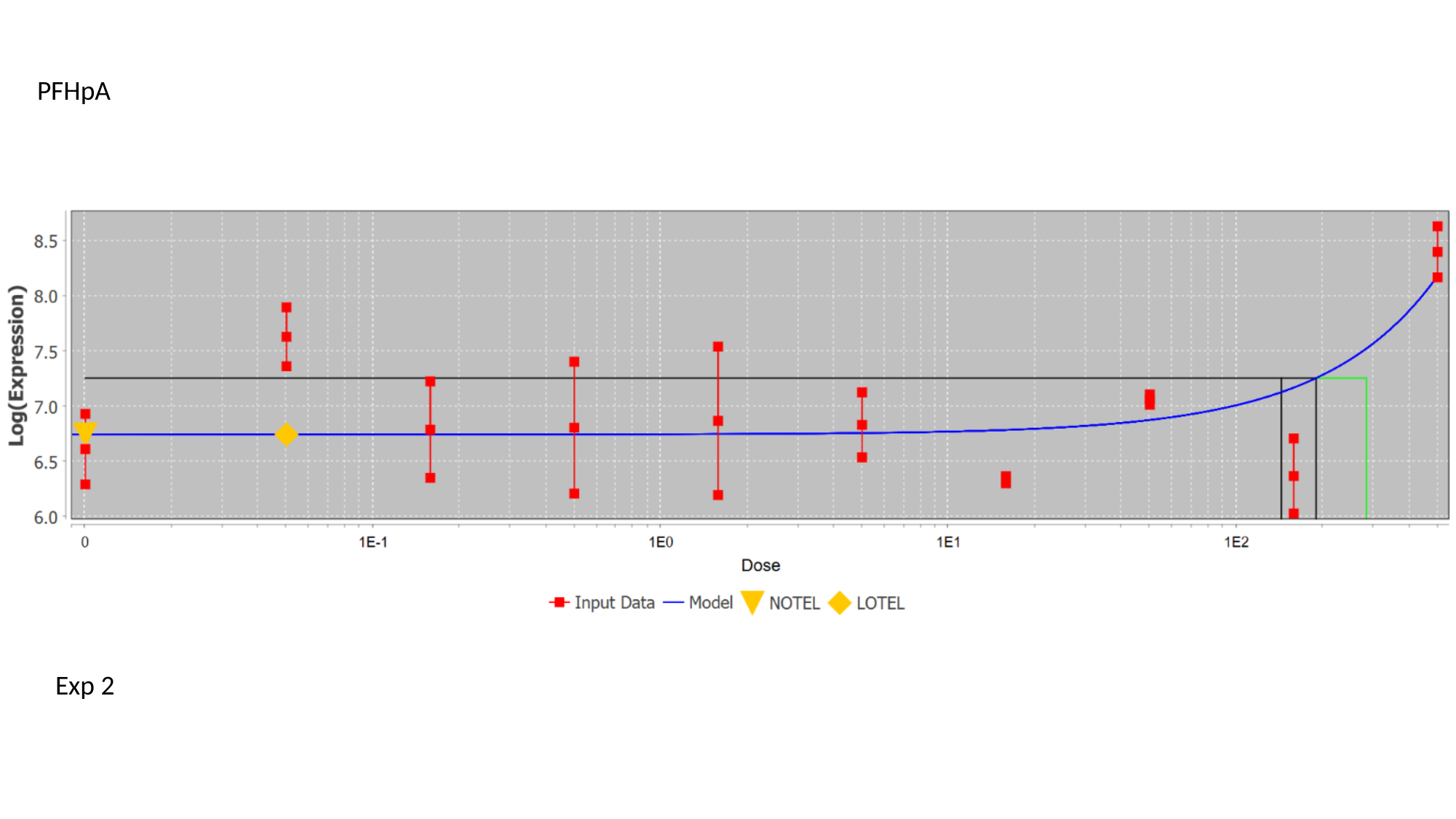

PFHpA
Exp 2

## Slide 14
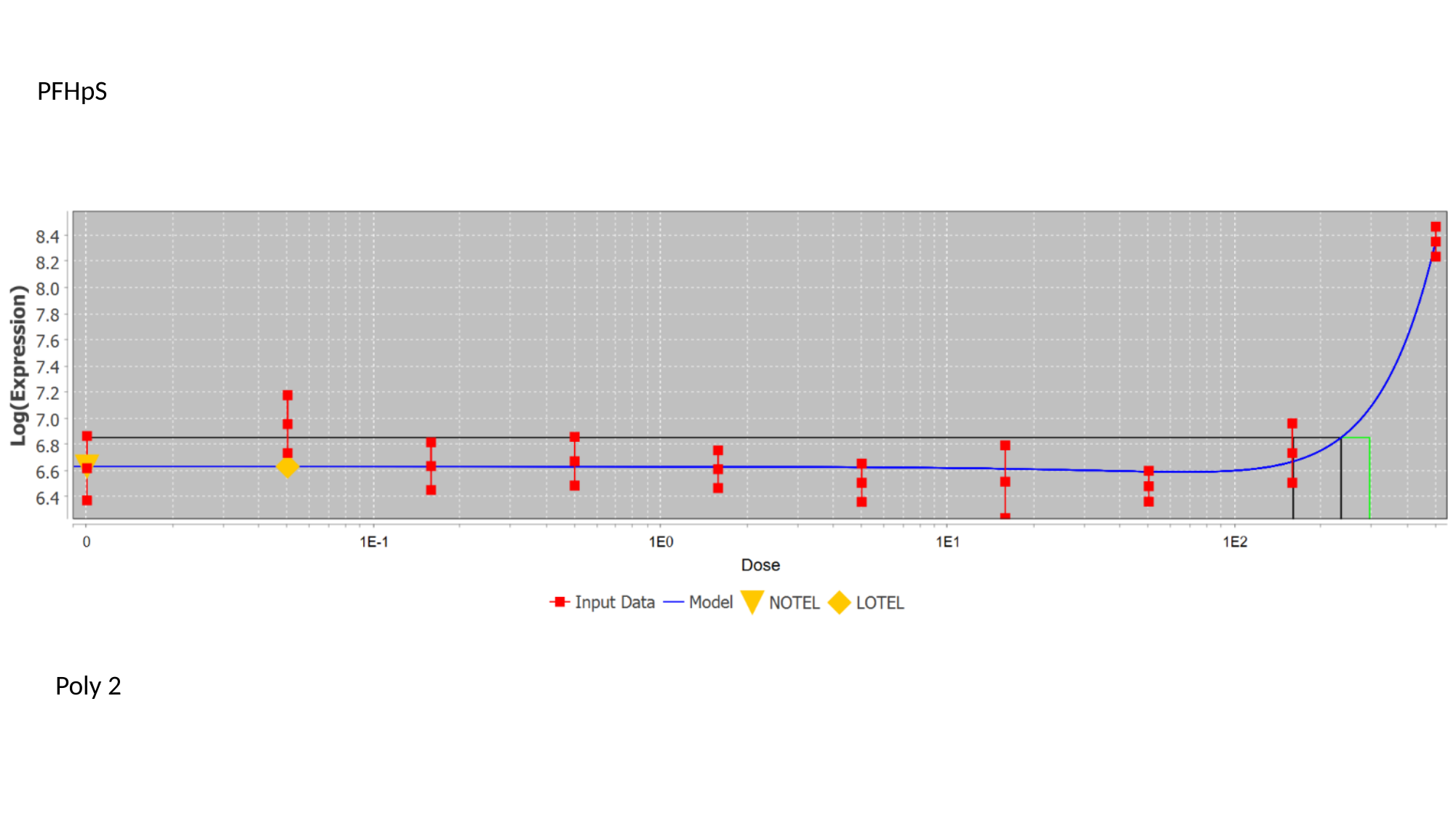

PFHpS
Poly 2

## Slide 15
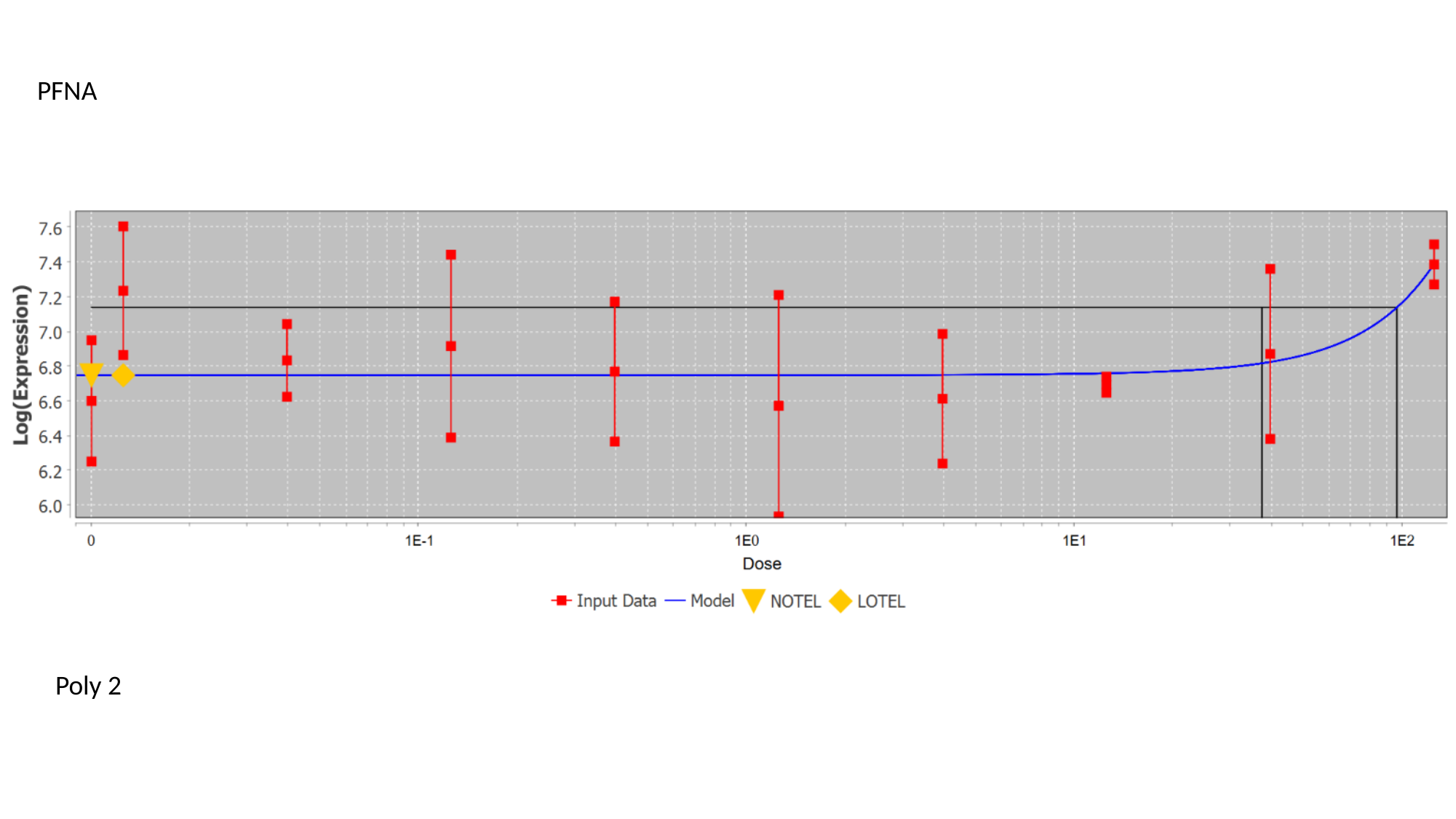

PFNA
Poly 2

## Slide 16
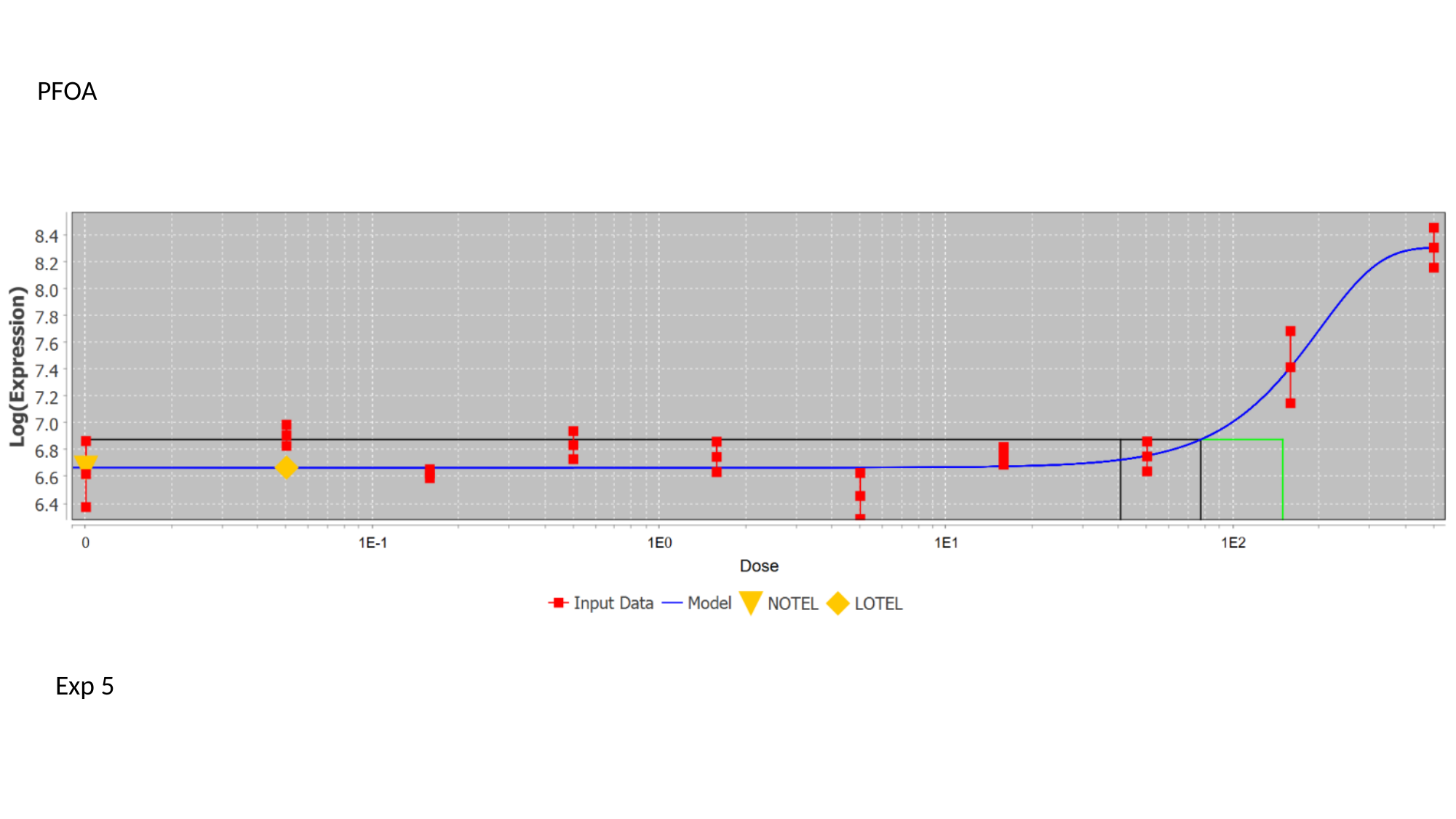

PFOA
Exp 5

## Slide 17
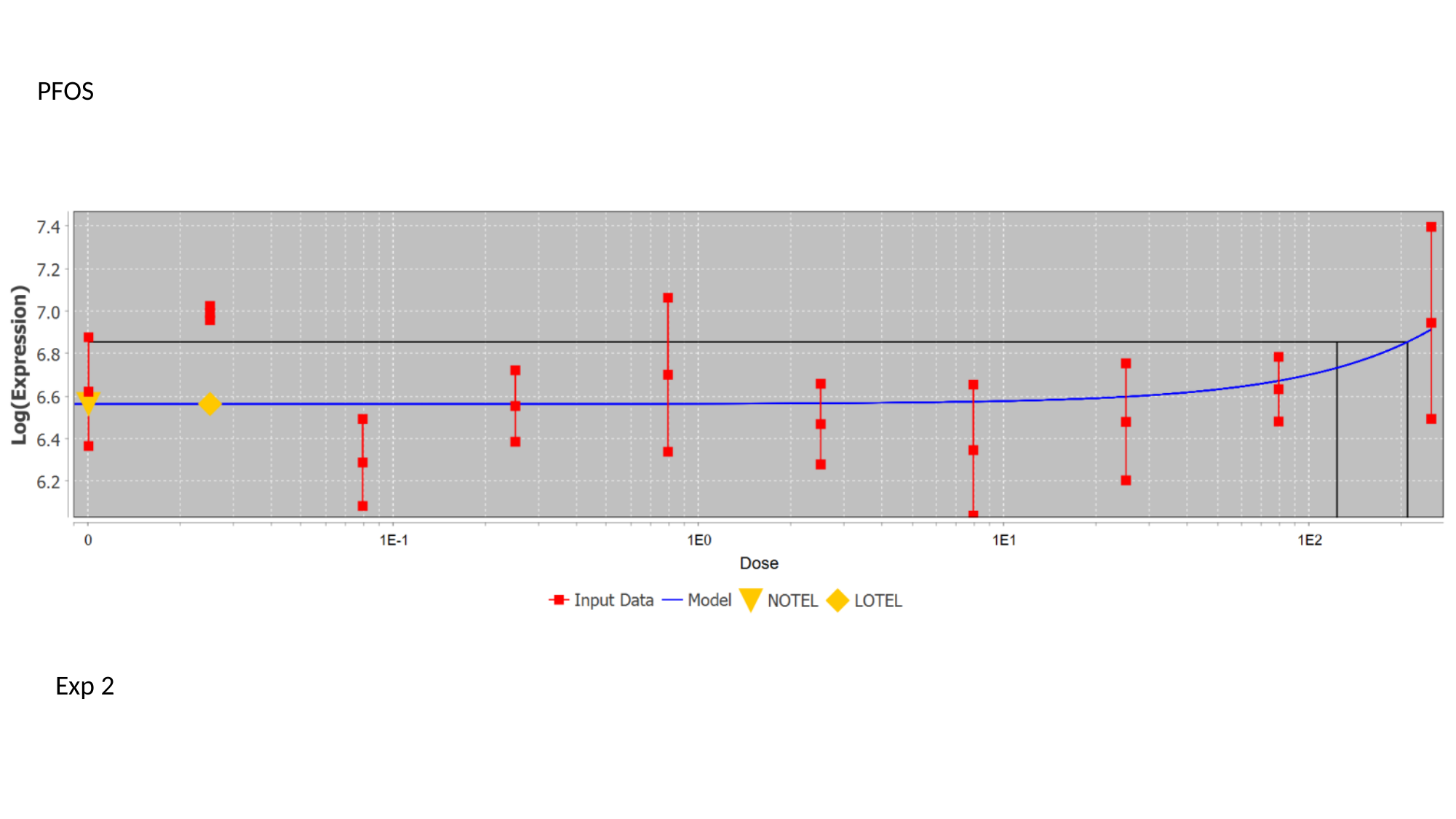

PFOS
Exp 2

## Slide 18
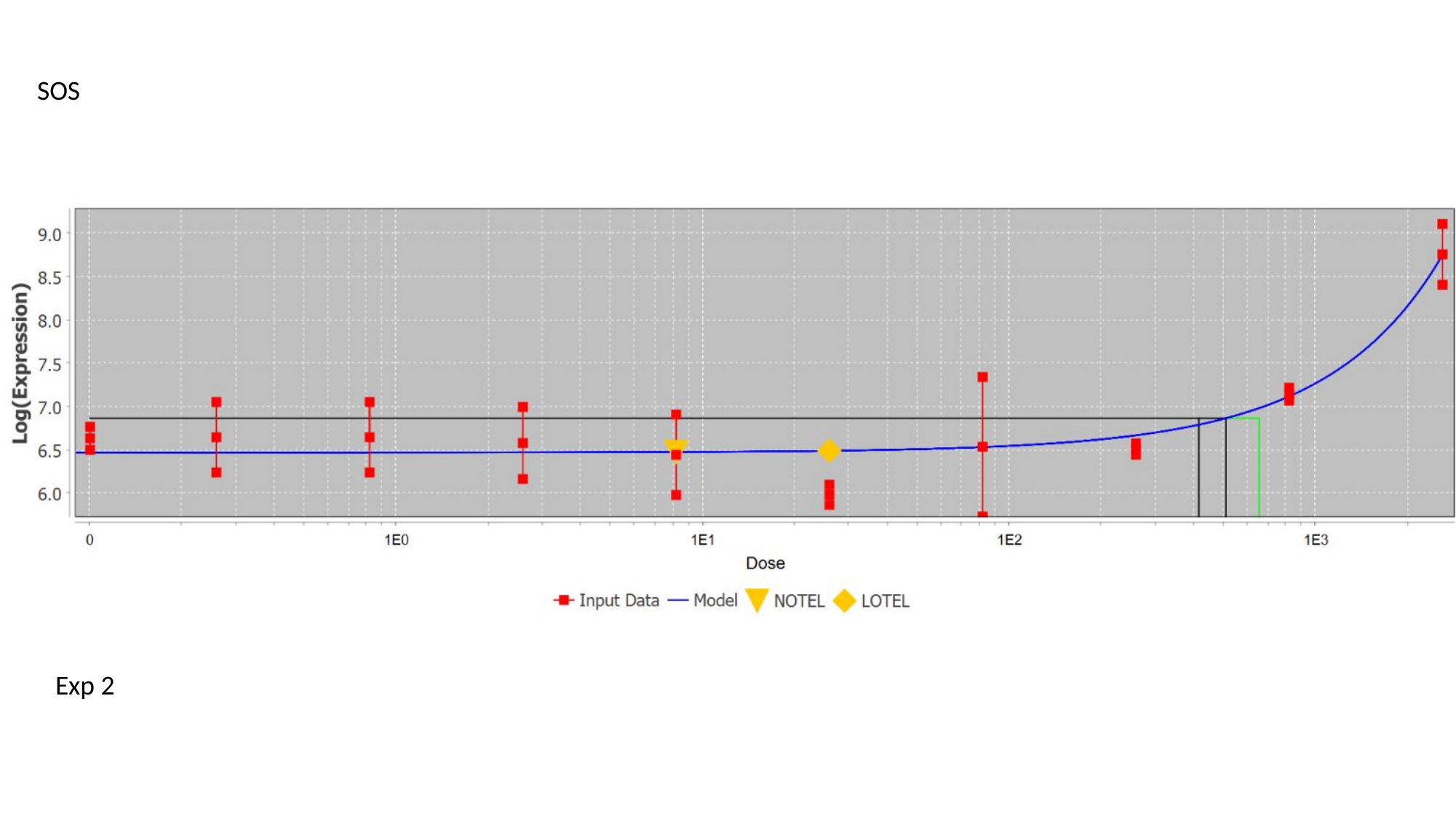

SOS
Exp 2

## Slide 19
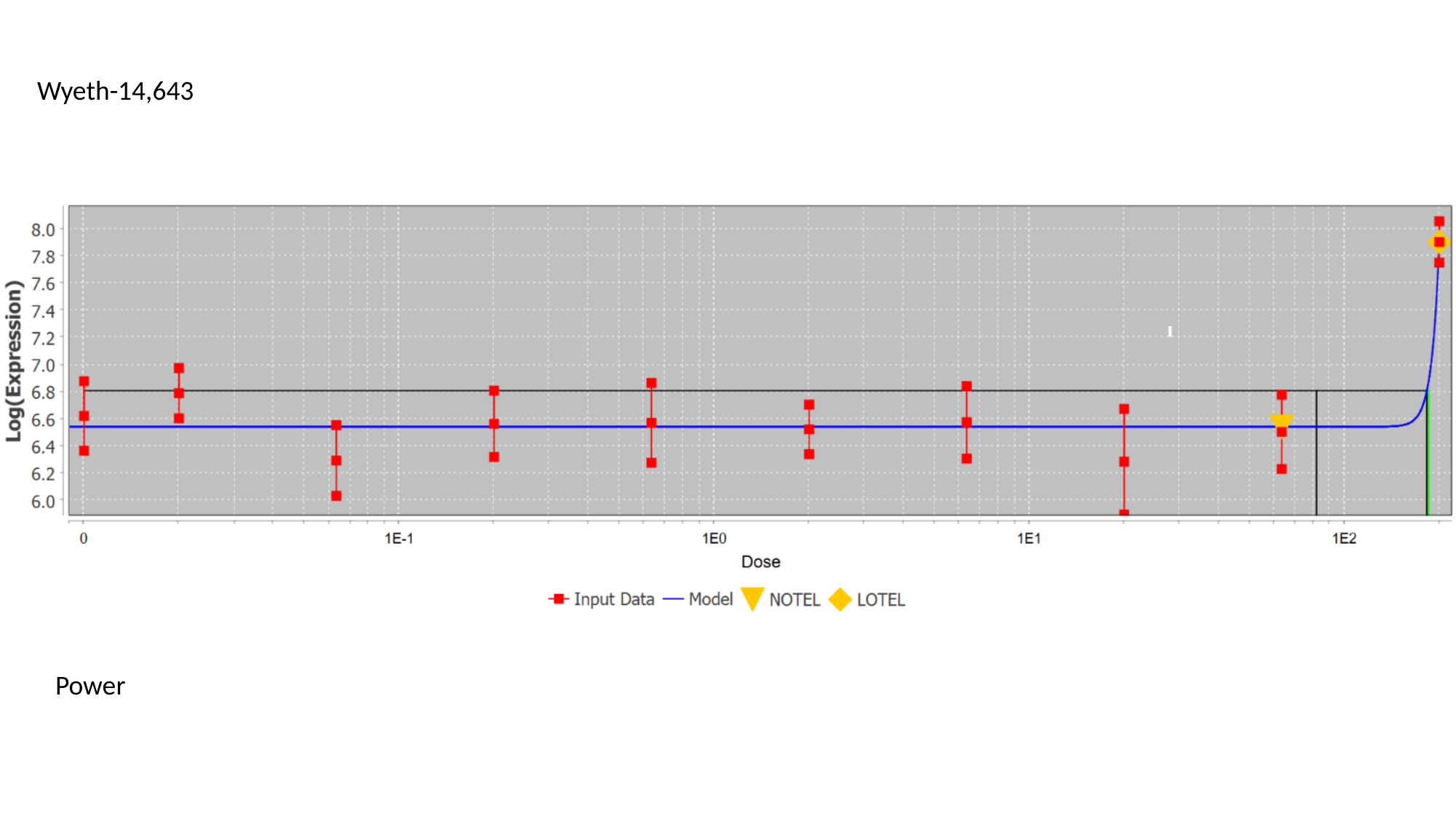

Wyeth-14,643
Power
